# ﻿Systematics of the *Trembleya* sensu stricto clade of *Microlicia* (Melastomataceae, Lavoisiereae)

**DOI:** 10.3897/phytokeys.216.91032

**Published:** 2022-12-20

**Authors:** Ricardo Pacifico, Frank Almeda, Darin S. Penneys, Karina Fidanza

**Affiliations:** 1 Universidade Estadual de Maringá, Programa de Pós-Graduação em Biologia Comparada. Av. Colombo, 5790, 87020-900 Maringá, Paraná, Brazil; 2 California Academy of Sciences, Institute for Biodiversity Science and Sustainability, Department of Botany, 55 Music Concourse Drive, Golden Gate Park, San Francisco, California 94118-4503, USA; 3 Department of Biology and Marine Biology, University of North Carolina Wilmington, Wilmington, North Carolina 28403, USA; 4 Departamento de Biologia, Universidade Estadual de Maringá, Av. Colombo, 5790, Jardim Universitário, CEP 87020-900, Maringá, Paraná, Brazil

**Keywords:** Brazil, Cadeia do Espinhaço, campo rupestre, Endemism, Minas Gerais

## Abstract

A systematic monograph of the *Trembleya* s.s. clade is presented, a Brazilian endemic lineage of Melastomataceae comprising 11 species and currently recognised as part of *Microlicia* s.l. (Melastomataceae). First, we investigate phylogenetic relationships within Lavoisiereae using two nuclear markers and two sampling datasets (102 and 134 terminals). Then, we provide a systematic revision and new circumscription of the *Trembleya* s.s. clade, including line drawings, photos of living specimens, leaves and floral parts, distribution maps, a key to the 11 accepted species, comments on morphology, reproductive biology, richness, endemism, biogeography and recommended conservation assessments. A nomenclatural update of all taxa previously treated in *Trembleya* is also provided, including the designation of 45 lectotypes and the proposal of 38 new synonyms.

## ﻿Introduction

Melastomataceae Juss. comprises about 5,880 species in 173 genera with about two-thirds of this diversity restricted to the neotropics (Ulloa Ulloa et al. 2022). Brazil is the most diverse country with 69 genera and about 1440 species and the family is represented in all Brazilian biomes except for the Caatinga *sensu stricto* (Goldenberg et al. 2020). The most comprehensive treatments of Brazilian Melastomataceae were produced by Cogniaux (1883–1888, 1891) in “Flora Brasiliensis”, which were largely based on tribal concepts proposed by Triana (1872). During the last century, the family has been treated in many regional “Floras” and several genera and numerous new species of Brazilian Melastomataceae were described (Goldenberg et al. 2012). Although the work of Cogniaux (1883–1888) has long been the primary global reference for the family, a new familial treatment for Brazil was recently published, including morphological descriptions and keys to all Brazilian genera and species (Goldenberg et al. 2020), as part of the ambitious Brazilian Flora 2020 (BFG 2022).

As compared to the treatment in “Flora Brasiliensis”, the most recent infrafamilial classifications of Melastomataceae show significant improvements. Based on DNA sequence data, several new Neotropical tribes have been delimited, i.e. Henrietteeae Penneys, Michelang., Judd & Almeda (Penneys et al. 2010), Marcetieae M.J.Rocha, P.J.F.Guim. & Michelang. (Rocha et al. 2018), Pyramieae Naudin (as Cambessedesieae Bochorny, Almeda, Michelang. & R.Goldenb.) (Bochorny et al. 2019), Rupestreeae Penneys & R.Goldenb., Stanmarkieae Penneys & Almeda (Penneys et al. 2022), Trioleneae Bacci, Michelang. & R.Goldenb. (Bacci et al. 2019), Eriocnemeae Penneys & Almeda and Lithobieae Penneys & Almeda (Penneys et al. 2020). Some new genera have also proposed, for example, *Rupestrea* R.Goldenb., Almeda & Michelang. (Goldenberg et al. 2015), *Physeterostemon* R.Goldenb. & Amorim (Goldenberg and Amorim 2006), *Brasilianthus* Almeda & Michelang. (Almeda et al. 2016) and others were relegated to synonymy (for example, see Penneys and Judd 2013 and Bochorny et al. 2019); and there are still unplaced clades and genera that need to be tested for monophyly (Penneys et al. 2022). Meanwhile, many new species are still being described from little-studied genera and/or resulting from fieldwork in areas that remain poorly known or underexplored botanically.

With about 275–300 species, the near-endemic Brazilian tribe Lavoisiereae DC., which has long been known by the later tribal name Microlicieae Naudin, is one of the richest clades of Neotropical Melastomataceae (Fritsch et al. 2004; Versiane et al. 2021; Pacifico and Almeda 2022). Currently, three genera are accepted, i.e. *Rhynchanthera* DC. (15 spp.; Renner 1990), *Poteranthera* Bong. (5 spp.; Kriebel 2012; Almeda and Pacifico 2018) and *Microlicia* D.Don (ca. 250 spp.; Versiane et al. 2021; Pacifico and Almeda 2022). However, this tribe has a complex taxonomic history and its current generic delimitation has been drastically modified in light of molecular evidence (Fritsch et al. 2004; Rocha et al. 2016; Versiane et al. 2021). Based on rpl16 and nrITS data, Fritsch et al. (2004) found support for the restriction of Lavoisiereae to the six genera previously enumerated by Almeda and Martins (2001). In turn, phylogenetic analyses of Rocha et al. (2016) were decisive in assigning *Poteranthera* to the tribe where it had been placed by Triana (1872) and several other early classifications, thus resulting in the recognition of seven genera in Lavoisiereae. Major taxonomic alterations were proposed by Versiane et al. (2021), who included four traditionally recognised genera (*Chaetostoma* DC., *Lavoisiera* DC., *Stenodon* Naudin, *Trembleya* DC.) in a broadly circumscribed *Microlicia* s.l. Versiane et al. (2021) recovered little support for most clades within *Microlicia* s.l., whereas some internal clades were strongly supported and are recognisable morphologically, e.g. the “*Trembleya* s.s.” “*Lavoisiera*” and “*Chaetostoma*” clades (Koschnitzke and Martins 2006; Martins and Almeda 2017; Pacifico et al. 2019; Versiane et al. 2021). For a complete taxonomic history of the tribe, see Martins and Almeda (2017: 32–34) and Pacifico and Almeda (2022).

*Trembleya* s.s. is one of the clades currently recognised as part of *Microlicia* s.l. (Versiane et al. 2021; Pacifico and Almeda 2022). We here re-evaluate the status of this lineage in a phylogenetic context and provide a systematic revision and new circumscription of the *Trembleya* s.s. clade. Line drawings, photos of living specimens, leaves and floral parts, distribution maps, a complete nomenclatural update, comments on morphology, reproductive biology, richness, endemism, biogeography and conservation are also provided.

## ﻿Historical background

*Trembleya* was named by Augustin Pyramus de Candolle (1778–1841) in honour of the members of the family Trembley for their contributions to several fields of science (Candolle 1828: 125). At that time, the genus was represented by six species, all of which were described, based on the collections made by Carl Friedrich Philipp von Martius in Brazil (Spix and Martius 1824). Candolle (1828: 114) placed *Trembleya* along with other 13 genera in the tribe Rhexieae DC. This arrangement changed about two decades later when Charles Victor Naudin (1818–1889) assigned this genus to the tribe Lavoisiereae (as “Microliciales”) (Naudin 1849: 203). *Trembleya* was maintained in the tribe Lavoisiereae (as “Microlicieae”) in subsequent treatments of Melastomataceae like those by Triana (1872) and Cogniaux (1883–1888, 1891).

*Trembleya* was recognised as a heterogeneous group of species since it was first delimited by Candolle (1828), who recognised three morpho-groups. Three sections were proposed, two of which were named in honour of members of the Trembley family (Candolle 1828). The section Jacobia DC. was named after Jacob Trembley and the section Abrahamia DC. was named after Abraham Trembley (Candolle 1828: 125–126). The remaining section, composed of only *Trembleyalychnitis* DC. [= *Microlicialaniflora* (D.Don) Baill.], was named *Erioleuca* DC. in reference to the lanose appearance of this species (Candolle 1828: 126). Candolle (1828b: 38) also claimed that these three sections could one day either be brought together through more nuanced intermediaries or separated into three distinct genera.

Chamisso (1834) followed Candolle’s infrageneric classification and described two new species in the genus: *Trembleyapithyoides* Cham. in section Jacobia and *Trembleyacalycina* Cham. in sect. Abrahamia. Naudin (1849: 264) agreed with Candolle (1828) on the polymorphic aspect of *Trembleya*, considering it a “g*enus habitu specierum omnino heteromorphum*” [genus in which the habit is highly heteromorphic amongst species]. Naudin (1849) reformulated the infra-generic classification of the genus under two new sections, Trembleyasect.Verae Naudin and Trembleyasect.Heterogenae Naudin.

In “Flora Brasiliensis”, Cogniaux (1883–1888) mostly maintained the sections proposed by Naudin (1849). Trembleyasect.Trembleya Cogn. contained the same species of Trembleya attributed tosect. sect. Verae, but included two taxa that were not available for study in Paris by Naudin, *Trembleyachamissoana* Naudin and *Trembleyarosmarinoides* Mart. & Schrank ex DC. In addition, Cogniaux (1883–1888) resurrected Candolle’s monotypic section Erioleuca, which included only *Trembleyalaniflora*. The section Heterogenae was also maintained by Cogniaux (1883–1888), who included three additional species that were described after Naudin’s monographs, *Trembleyaselloana* Cogn., *Trembleyawarmingii* Cogn. and *Trembleyapradosiana* Netto.

Renner (1990: 609) stated that *Trembleya* was “an assemblage of species differing greatly in habit and pubescence”. Martins (1995) described another species, *Trembleyahatschbachii* Wurdack & E.Martins. In the unpublished monograph of *Trembleya* by Martins (1997), 18 species were recognised (several never validly published) and three dubious taxa were noted. Martins (1997) did not recognise any of the infrageneric sections previously proposed in the genus.

Martins (1997) made a special effort to understand the supra-generic delimitation of *Trembleya*. She emphasised that the number of ovary locules was the only feature used by Cogniaux (1883–1888) to differentiate this genus from *Microlicia* D.Don (3-locular in *Microlicia* and 5-locular in *Trembleya*). In this context, a major objective of Martins (1997) was to clarify the limits between *Trembleya* and *Microlicia*. Based on comparative morphology, Martins (1997) proposed an expanded re-circumscription of *Trembleya*, which was based on characters, such as inflorescence structure, vegetative architecture and leaf venation patterns. However, Martins (1997) recognised that none of these features could differentiate *Trembleya* from *Microlicia* in an effective way (Martins 1997). Besides, Martins (1997) mentioned taxa with overlapping features, such as *Microliciapabstii* Brade, that has leaves with evident secondary venation, a feature attributed only to *Trembleya* (sensu Martins 1997) and isolated axillary flowers, a typical feature of *Microlicia* (Martins 1997).

Fritsch et al. (2004) confirmed the placement of *Trembleya* in Lavoisiereae using molecular data. Subsequently, new species were described in *Trembleya* by Fidanza et al. (2013), Pacifico and Fidanza (2015) and Pacifico et al. (2019). Additionally, one species was transferred from *Lavoisiera* DC. to *Trembleya* by Almeda and Martins (2001) [*Trembleyaelegans* (Cogn.) Almeda & A.B.Martins], and another was transferred from *Trembleya* to *Poteranthera* by Almeda and Pacifico (2018) [*Poterantherawarmingii* (Cogn.) Almeda & R.B.Pacifico]. Pacifico et al. (2019) argued that *Trembleya* was polyphyletic and a new circumscription was necessary in order to reduce this genus to a monophyletic group of 11 species (*Trembleya* s.s.).

Based on an expanded phylogenetic analyses using five molecular markers (atpF-atpH, trnS-trnG, nrITS, nrETS and waxy) and 12 morphological characters for 113 taxa in Lavoisiereae, Versiane et al. (2021) transferred all species of *Trembleya* to the enlarged *Microlicia* s.l. In order to accommodate these species in *Microlicia*, Versiane et al. (2021) proposed 13 new combinations [*M.altoparaisensis* (R.B.Pacifico, Fidanza & Almeda) Versiane & R.Romero, *M.calycina* (Cham.) Versiane & R.Romero, *M.chamissoana* (Naudin) Versiane & R.Romero, *M.inversa* (Fidanza, A.B.Martins & Almeda) Versiane & R.Romero, *M.neopyrenaica* (Naudin) Versiane & R.Romero, *M.parviflora* (D.Don) Versiane & R.Romero, *M.pentagona* (Naudin) Versiane & R.Romero, *M.phlogiformis* (Mart. & Schrank ex DC.) Versiane & R.Romero, *M.pithyoides* (Cham.) Versiane & R.Romero, *M.pradosiana* (Netto) Versiane & R.Romero, *M.rosmarinoides* (Mart. & Schrank ex DC.) Versiane & R.Romero, *M.thomazii* (R.B.Pacifico & Fidanza) Versiane & R.Romero, *M.tridentata* (Naudin) Versiane & R.Romero)] and three new names [*M.acuminifolia* Versiane & R.Romero, *M.flaviflora* Versiane & R.Romero, *M.serratifolia* Versiane & R.Romero], whereas *M.laniflora* (D.Don) Baill. was reinstated. The analyses of Versiane et al. (2021) also supported the recognition of *Trembleya* s.s. as a distinct clade, as previously suggested by Pacifico et al. (2019). Although recognisable morphologically, the *Trembleya* s.s. clade was transferred to *Microlicia* by Versiane et al. (2021) because the recognition of that clade as a distinct genus would make *Microlicia* s.l. paraphyletic. This issue is also re-evaluated in the present study.

## ﻿Material and methods

### ﻿Taxa sampling and DNA extraction

To investigate phylogenetic relationships in Lavoisiereae, we used two different datasets including samples from the seven genera traditionally recognised in the tribe (Almeda and Martins 2001; Fritsch et al. 2004). Dataset A comprised samples with complete sequences (i.e. fitting perfectly the alignment on both ends) of both nrITS and nrETS. This dataset had 102 terminals, representing 99 species (ingroup, 91 species; eight species are outgroups). Dataset B comprised samples with complete nrITS and nrETS sequences, but also samples lacking sequences of one of these markers and/or partial sequences, including 134 terminals representing a total of 131 species (ingroup, 127 species; four species are outgroups). Overall, taking the two datasets into consideration, 247 sequences were generated for this study (125 nrITS plus 122 nrETS; total of 131 species). Eight sequences were retrieved from GenBank and added to the alignment (seven nrETS plus one nrITS; total of seven species). For information on specimen vouchers and GenBank accessions, see Suppl. material 1.

Total genomic DNA was extracted from silica-dried leaves or from herbarium specimens. DNA extraction was performed mainly using Qiagen DNeasy plant kits, following the manufacturer’s recommendations (https://www.qiagen.com/) or modified to extend the incubation time in AP1 Buffer and RNAase to 1 hour, followed by 1 hour of precipitation in ice. A few samples were extracted using the modified CTAB (hexadecyltrimethylammonium bromide) protocol of Doyle and Doyle (1987). Leaves were ground in a mixer mill, incubated in 1.2 ml CTAB and 5 µl of Proteinase K for 60–180 minutes at 55 °C. DNA was precipitated overnight in isopropanol and sodium acetate at -20 °C, centrifuged at 13,000 rpm for 20 minutes, washed twice with 70% ethanol and dried at room temperature. The pellets with DNA were then resuspended in 100 µl of 1× TE (tris-EDTA buffer, pH 8.5) and incubated at 55 °C for about 30 minutes.

### ﻿PCR and DNA sequencing

We amplified and sequenced the ribosomal markers nrETS and nrITS. In Melastomataceae, the nrETS region has been used by Kriebel et al. (2015), Rocha et al. (2016), Bacci et al. (2020) and Versiane et al. (2021). The nrITS is widely used in phylogenetic studies of the family (e.g. Ionta et al. 2007; Penneys and Judd 2013; Reginato and Michelangeli 2016; Zhou et al. 2018). Information on the primers used in this study may be found in the aforementioned literature. The PCR recipes were: (1) for nrETS, 1.5 μl of Buffer 10×, 0.9 μl of MgCl_2_ 25 uM, 0.3 μl of each primer (10 uM), 0.3 μl of dNTP (10 uM), 0.3 μl of Hotstart *Taq* polymerase (1.25 uM) and 1 μl of template, reaching 15 μl of final reaction; (2) for nrITS, the recipe was similar to (1), but differed in including 1.2 μl of BSA (10 uM) and using Invitrogen *Taq* polymerase to improve the performance. The thermocycler programmes were: (1) for nrETS initial denaturation at 95 °C for 2 minutes, 40 cycles of denaturation at 94 °C for 30 seconds, annealing at 58 °C for 30 seconds; extension at 72 °C for 1 minute and final extension at 72 °C for 7 minutes; (2) for nrITS, initial denaturation at 94 °C for 5 minutes, 40 cycles of denaturation at 94 °C for 10 seconds; annealing at 61 °C for 45 seconds; extension at 72 °C for 40 seconds and final extension at 72 °C for 10 minutes. The quality of amplifications was verified in agarose electrophoresis gel. The amplified fragments were cleaned using 5 μl of PCR products (7 μl for weak bands) and 2 μl of diluted ExoSAP-IT. The PCR cleaning was performed by incubating the samples at 37 °C for 30 minutes and 80 °C for 15 minutes. Cycle sequencing was performed by using the same amplification primers. The thermocycler programme used for cycle sequencing began at 96 °C for 60 sec, followed by 15 cycles of 10 sec at 96 °C, 5 sec at 50 °C and 75 sec at 60 °C; another 5 cycles of 10 sec at 96 °C, 5 sec at 50 °C, 90 sec at 60 °C; 5 sec at 50 °C and 120 sec at 60 °C. The Sanger Sequencing was performed in an ABI 3130 Genetic Analyzer in the Center for Comparative Genomics at the California Academy of Sciences, San Francisco, CA. GenBank accessions for all sequences used in this study are provided in Suppl. material 1.

### ﻿Phylogenetic analyses

Contigs were assembled and edited using Geneious 11.0 (https://www.geneious.com). The same software was employed to align (using Muscle algorithm), concatenate and export the sequences as fasta files. Low-quality sequences were discarded. Final manual edits on the alignment were performed using Mega 7 (Kumar et al. 2016). Maximum Likelihood (ML) and Bayesian Inference (BI) phylogenetic analyses were performed using the concatenated sequences of dataset A, whereas dataset B was only analysed using ML. All analyses were run through the CIPRES online gateway (http://www.phylo.org/; Miller et al. 2012) as four independent partitions (*nrETS*, *ITS1*, *5.8S* and *ITS2*). The ML analyses were performed using a fast Maximum Likelihood tree search algorithm (RAxML, Stamatakis 2014) with the GTR+Γ+I model and 1,000 bootstrap repetitions. The BI analysis was performed with MrBayes version v.3.2.6 using the same model (GTR+Γ+I), which was employed for being the most parameter-rich (see Abadi et al. 2019). BI analyses consisted of two independent runs, each one with one cold and three heated simultaneous independent Markov chains. A total 15,000,000 generations were carried out, sampling one tree every 1000 generations and discarding 25% of the sampled trees as burn-in. A node was considered to be strongly supported if it showed both a Bayesian Posterior Probability ≥ 0.95 and a Bootstrap Support ≥ 70%.

### ﻿Taxonomic study

Specimens of all species of the *Trembleya* s.s. clade were examined. This study was carried out using collections from the following herbaria: BM, BR, C, CAS, CESJ, ESA, F, G, GH, HB, HUEM, INPA, K, L, M, MBM, MO, NY, P, R, RB, S, SPF, UEC, US and W (acronyms follow Thiers 2022). Specimens of some of the mentioned herbaria were examined only through online images (BM, C, CESJ, ESA, G, GH, INPA, L, M, MO and S). All types and their synonyms were examined directly or through online images available from databases, for example, JSTOR Global Plants (https://plants.jstor.org/) and SpeciesLink (http://www.splink.org.br/). A list with all specimens cited in this study is provided in Appendix 1.

Populations of all but two species (*M.rosmarinoides* and *M.trembleyiformis*) of the *Trembleya* s.s. clade were sampled in the field during several expeditions carried out by the authors, mainly in the following regions of Brazil: Serra do Cipó, Serra do Cabral, Serra do Bota, Serra de Grão Mogol, Serra do Caraça, Serra do Gandarela, Serra de Ouro Preto and on the Diamantina Plateau (in Minas Gerais State), Chapada Diamantina (Bahia State), Chapada dos Veadeiros and Serra dos Pirineus (Goiás State) and Parque Estadual do Guartelá (Paraná State). All specimen identifications were carefully checked by the authors. We considered one specimen as a putative hybrid between *M.pentagona* and *M.laniflora* because it clearly intermixes characters between these two species and was collected in an area where they occur sympatrically.

The morphological diagnosis was based on a comparative study of species in the clade and all remaining taxa within Lavoisiereae. Species descriptions, lists of specimens examined and circular histograms of phenology were prepared using the functions “TabletoDescription”, “Collector List” and “PhenoHist” (respectively), available from the package monographaR implemented in R (Reginato 2016). Herbarium specimens were also the source of all distributional data used in this study. All coordinates obtained from specimen labels were mapped to verify if they matched the locality described.

### SEM images

Scanning Electron Microscope (SEM) photos were taken from herbarium specimens. Sections of leaf blades (all species in the clade), anthers (four species), seeds (five species) and pollen grains (*M.pithyoides*) were affixed using double-stick tape on pin stubs, sputter-coated with gold and examined using a Hitachi SU3500 microscope with coupled digital camera in the SEM Lab of the California Academy of Sciences, San Francisco, CA.

### ﻿Anatomical studies

Dried leaves of *M.altoparaisensis*, *M.chamissoana* and *M.flaviflora* were obtained from exsiccatae (housed in HUEM), rehydrated in sodium hydroxide (NaOH) 5% (Anderson 1963, modified) and stored in 70% methyl alcohol (Johansen 1940). Epidermal dissociation was performed either using acetic acid and hydrogen peroxide (1:1) (Franklin 1945) or 10% nitric acid and 10% chromic acid (1:1) (Johansen 1940). Epidermal sections were stained using Safranine and Astra blue (Bukatsch 1972) and mounted in glycerine 50% (Kraus and Arduin 1997). Samples of the leaf blade were embedded in historesin (2-hydroxyethylmethacrylate). Cross and longitudinal sections of the leaf blade (7 μm) were obtained using a rotary microtome and stained with 0.05% toluidine blue, acetate buffer at pH 4.7 (O’Brien et al. 1965, modified). Light micrographs were taken using a Leica EZ4D digital camera in the Department of Biology of the Universidade Estadual de Maringá.

### ﻿Biogeography and mapping

Species richness and weight endemism analyses were run in R through the Dinamica Ego platform (http://www.dinamicaego.com) using the tools “SR” and “WE” from the toolkit BioDinamica 2.2 (Oliveira et al. 2019). In both cases, the distributional data were saved as a csv file and analysed by employing a grid of hexagons in which the distances amongst centroids were 1°. We delimited the main area of endemism for the *Trembleya* s.s clade based on endemicity analyses implemented in the NDM/VNDM software (Szumik et al. 2002; Szumik and Goloboff 2004). A spreadsheet with the distributional data was saved as a csv file and converted into a xyd file using the GeX online tool (http://gex.mfuhlendorf.com/ (Santos and Fuhlendorf 2018). In NDM/VNDM, we used a grid of 1° cells and searched for Areas of Endemism with 1000 repetitions. Only individual areas of endemism with scores equal to or higher than 2 were considered and overlapping subsets were kept only if they had at least 40% unique species. The consensus area of endemism was obtained using the loose consensus rule and a cut-off value of 40% (see Aagesen et al. 2013). Coordinates of the consensus area were exported from NDM/VNDM as a text file and converted into a shapefile using DIVA-GIS (Hijmans et al. 2001). All maps were prepared using QGIS 3.14 software (QGIS Development Team 2021).

### ﻿Conservation assessments

Following the guidelines of criterion B of IUCN (2019), we provide informal conservation status recommendations for each species, based on georeferenced data from herbarium material. GeoCAT (Bachman et al. 2011) was used to calculate Extent of Occurrence (EOO) and Area of Occupancy (AOO) for each species using a user-defined cell width of 2 km. We are aware that the AOO is often inappropriately used for conservation assessments, as most plant species are represented in herbaria by only a few specimens, thereby underestimating the AOO (Lughadha et al. 2019). For Lavoisiereae, our field experience showed that most species in this tribe are often represented only by a few populations restricted to rocky outcrops on a shallow layer of white quartzitic soil. These populations usually occupy an area smaller than 4 km^2^; thus, we believe that the cell width of 2 km is appropriate for these analyses.

## ﻿Results and discussion

### ﻿Molecular phylogenetics

**Figure 1.**
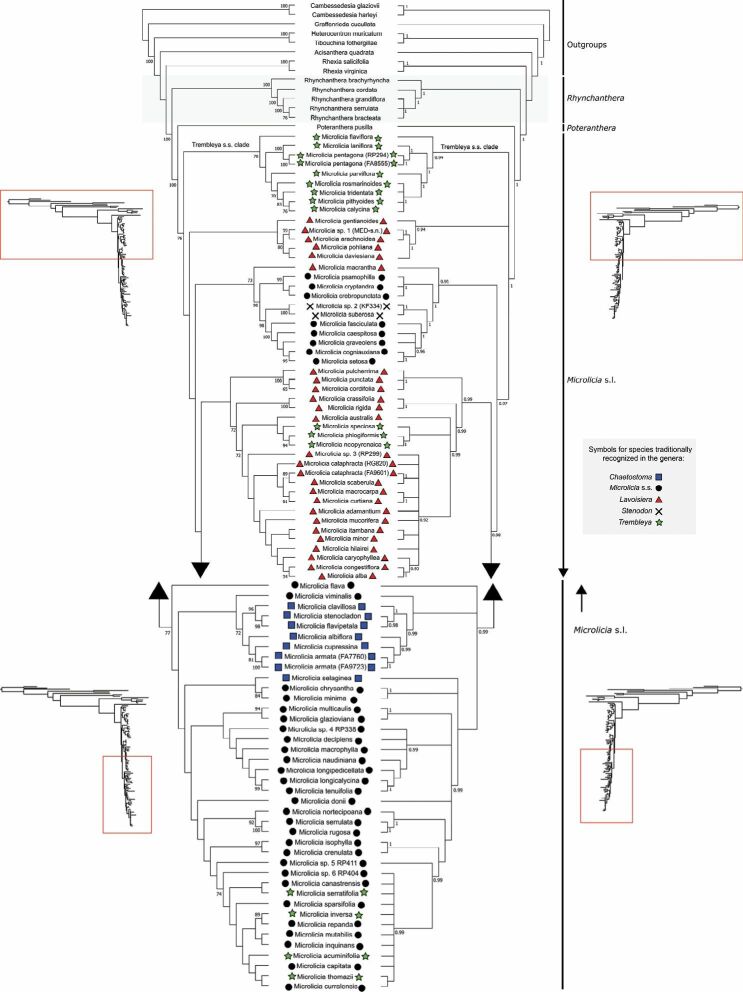
Maximum Likelihood (left) and Bayesian Inference (right) trees of Lavoisiereae, based on analyses of nrITS+nrETS sequences using dataset A. Bootstrap support values ≥ 70% and Bayesian Posterior Probability values ≥ 0.9 are given at each node.

**Figure 2.**
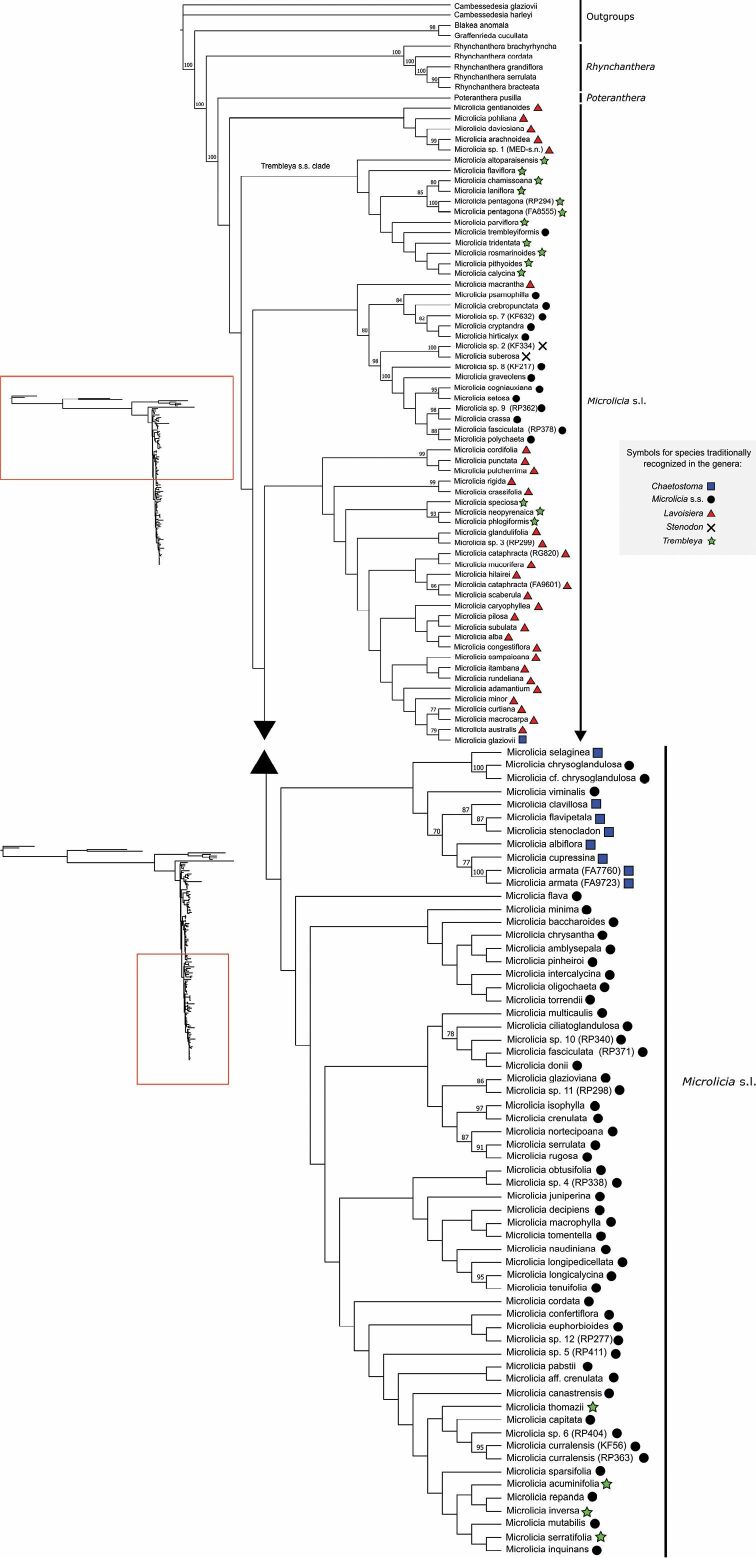
Maximum Likelihood tree of Lavoisiereae, based on analyses of nrITS+nrETS sequences using dataset B. Bootstrap support values ≥ 70% at each node.

The aligned matrix of the combined nrETS+nrITS contains 1435 characters. In all analyses, Lavoisiereae was recovered as monophyletic with strong statistical support, in agreement with the analyses of Fritsch et al. (2004) and Versiane et al. (2021). *Rhynchanthera* was recovered as the first diverging lineage (as reported by Fritsch et al. 2004 and Versiane et al. 2021), followed by *Poteranthera* and *Microlicia* s.l. (in agreement with Versiane et al. 2021; Figs 1, 2). In this context, even with the inclusion of *Poteranthera* by Rocha et al. (2016), the Lavoisiereae can be distinguished amongst other capsular-fruited Melastomataceae by its elongate to reniform seeds with a reticulate-foveolate testa, stamens forming well-developed pedoconnectives and anthers with rostrate thecae (Fritsch et al. 2004; Rocha et al. 2016; Pacifico and Almeda 2022).

Overall, species traditionally recognised in *Trembleya* (for example, by Cogniaux 1883–1888, 1891 and Martins 1997) were recovered in three distinct regions of the Lavoisiereae tree. Using the dataset A, a clade composed exclusively of species of *Trembleya* s.s. was recovered as sister to the rest of *Microlicia* s.l. in the ML tree, with a Bootstrap Support of 70% (Fig. 1). However, this sister relationship was unresolved in the BI tree (Fig. 1). Several clades within *Microlicia* s.l. had low topological resolution in all analyses and especially using dataset B (Fig. 2).

The analyses of nrETS and nrITS resulted in incompletely resolved trees with several weakly-supported clades, making it impossible to evaluate the monophyly of most clades within *Microlicia* s.l. This was expected since these lineages have been recovered with very short branch lengths in family-level phylogenetic analyses, based on the multi-loci approach that used a larger sampling of Melastomataceae (Penneys et al. 2022). However, a clade that is diagnosable morphologically and is a good match to *Trembleya* s.s. of Pacifico et al. (2019) was identified. The recovery of this clade as sister to the remaining *Microlicia* s.l. in the ML tree, based on complete nrETS and nrITS sequences, suggests that this group of species could be treated as a distinct genus. This taxonomic decision was neither confirmed nor refuted by both the BI tree using dataset A and the ML tree using dataset B. Thus, we here tentatively treat the *Trembleya* s.s. clade as part of *Microlicia*. Future studies using enhanced taxonomic characters may provide a definitive assessment of the limits between this clade and *Microlicia* s.l.

### ﻿General characterisation

#### Habit and branching

**Figure 3.**
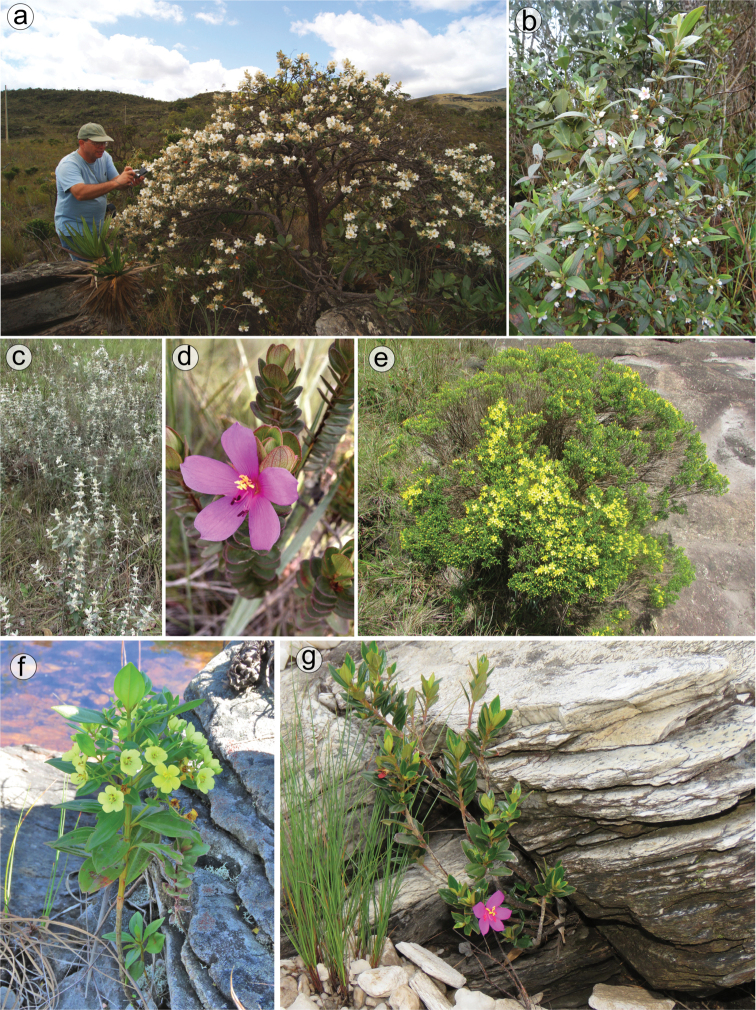
Habit in the *Trembleya* s.s. clade of *Microlicia***A** Celso de Paiva (ICMBio) taking a picture of a small *M.laniflora* tree **B***M.parviflora*, small tree **C***M.laniflora* with divaricate habit **D***M.chamissoana* with divaricate habit **E***M.rosmarinoides*, a small, much-branched shrub **F, G** Saxicolous shrubs: **F***M.flaviflora***G***M.pentagona*. Photos: **A** by O. Graeff **B** by R. Penati **C, E** by L. Pedrosa **D, F, G** by R. Pacifico.

Species of the *Trembleya* s.s. clade are woody and perennial erect shrubs, usually 0.5–2 m tall, small trees 2.5–4 m tall (e.g. *M.laniflora* and *M.parviflora*) or caespitose shrubs (e.g. *M.pithyoides* and *M.rosmarinoides*). Species can be highly variable in habit, like *M.laniflora*, which is typically a small tree (1.7–2.5 m tall), but may also be a shrub up to 0.8 m tall with divaricate branching (Fig. 3). Both forms may occur in the same population. The small trees of *M.laniflora* stand out by having a single trunk with corky bark. Variation in habit is also common in the wide-spread *M.parviflora*, generally from small shrubs ca. 0.5 m tall to small trees to 3–4 m tall. On the Serra do Cipó (Minas Gerais), a “dwarf” specimen of *M.chamissoana* measuring about 10 cm tall when flowering was collected once (*Giulietti et al CFSC12492*). The branching pattern in the *Trembleya* s.s. clade is consistently di-trichotomic, with quadrangular branchlets that are unwinged or have narrow wings ca. 0.2 mm wide. The branchlets are typically glandular-punctate and may be covered by other types of trichomes of variable type and distribution. Unlike many taxa of the *Lavoisiera* clade of *Microlicia* s.l. (Martins and Almeda 2017), all species in the *Trembleya* s.s. clade do not present conspicuous scars where the leaf has fallen away.

#### Root and stem anatomy and development

Information on root and stem anatomy and development is limited to the study of Somavilla and Graciano-Ribeiro (2012) on *M.parviflora*. The roots of *M.parviflora* have a uniseriate epidermis with outer periclinal walls and part of the anticlinal walls thickened and the cuticle thinner than the outer periclinal wall. In more developed roots, the epidermis is replaced by exodermis (Somavilla and Graciano-Ribeiro 2012). Likewise, the stems of *M.parviflora* have a uniseriate epidermis with outer periclinal walls slightly thickened and covered by a cuticle (Somavilla and Graciano-Ribeiro 2012). The cortex and the centre of the stems are separated by an endodermis layer and the central part of the cortex has large intercellular spaces (Somavilla and Graciano-Ribeiro 2012). The pericycle consists of 1–2 layers of parenchymatic cells surrounding the vascular system and the pith is parenchymatic (Somavilla and Graciano-Ribeiro 2012).

*Microliciaparviflora* stands out by having a spiralled aerenchymatous polyderm surrounding the root and stem (Somavilla and Graciano-Ribeiro 2012). In this species, the secondary growth of the stems and roots starts with the development of the vascular cambium, as well as the phellogen from pericycle cells. Derivative cells from phellogen then differentiate externally into two cell types disposed in concentric layers and centrifugally intercalated (Somavilla and Graciano-Ribeiro 2012). The one-layered phelloderm is produced centripetally by the phellogen (Somavilla and Graciano-Ribeiro 2012).

#### Leaf morphology

**Figure 4.**
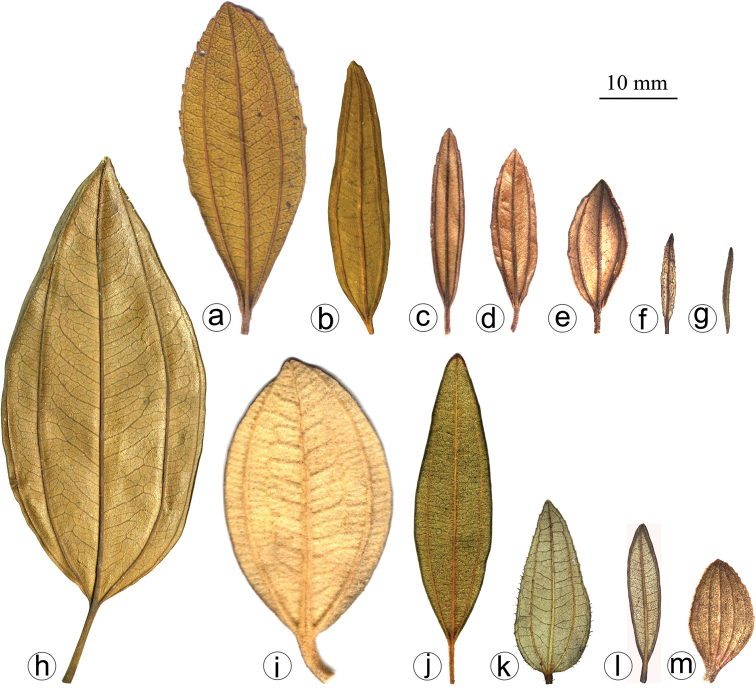
Leaves in the *Trembleya* s.s. clade of *Microlicia* in abaxial view **A***M.tridentata***B***M.altoparaisensis***C–E***M.pentagona***F***M.rosmarinoides***G***M.pithyoides***H***M.flaviflora***I***M.laniflora***J***M.parviflora***K***M.trembleyiformis***L***M.calycina***M***M.chamissoana*. Voucher specimens: **A** Longhi-Wagner et al. CFCR9184 (SPF, UEC, US) **B** Glaziou 21300 (F, P, S) **C** Barreto 10734 (BHCB) **D** Barreto 7025 (BHCB) **E** Rapini et al. 296 (HUEM, SP, SPF) **F** Souza et al. 2584 (BHCB) **G** Pacifico 295 (CAS, HUEM, SPF) **H** Hatschbach et al. 52005 (S) **I** Pacifico 185 (HUEM, SPF) **J** Pacifico 191 (HUEM) **K** Romero & Nakajima 3593 (HUFU, K, UEC) **L** Pacifico 290 (CAS, SPF) **M** Romero et al. 8627 (HUEM, HUFU, RB).

Leaf shape is highly variable and, together with indumentum and venation, it is one of the most taxonomically informative characters in the *Trembleya* s.s. clade (Fig. 4). Leaves are opposite and decussate like most Melastomataceae, highly variable in size along the branches and usually not imbricate. All species of the *Trembleya* s.s. clade have petiole features that are shared in a consistent way only with *Rhynchanthera* (see Renner 1990). The leaf blades vary from linear to oblong, lanceolate, narrowly-elliptical, elliptical, ovate or rarely slightly obovate. Leaf consistency varies from papyraceous to chartaceous or coriaceous. In 10 out of 11 species recognised in the *Trembleya* s.s. clade, the leaf blades become strongly discoloured when dry. The blade is usually plane throughout, although (when dry), the marginal region may become slightly revolute (in three species) or conspicuously revolute in *M.calycina*. The leaf blade is never keeled like it is in species of the *Chaetostoma* clade (Koschnitzke and Martins 2006). The leaf margin is entire, slightly serrulate or entire along the basal half and serrulate at the upper half; it lacks the stout glandular trichomes typical of *Poteranthera* (Almeda and Pacifico 2018). It is also not callose-thickened like in most species of the *Lavoisiera* clade (Martins and Almeda 2017). The leaves always have 1–3 primary basal veins and two or more pairs of secondary basal acrodromous veins (see Carmo et al. 2019). In most species, the most external pair of acrodromal veins is tenuous and positioned close to the leaf margin. Ten out of 11 species have tertiary veins more or less evident on the abaxial leaf surface, which are arranged nearly perpendicular to the mid-vein and may branch randomly or only apically. When mature, the leaf adaxial surface is always glandular-punctate to glabrescent. The abaxial surface is always glandular-punctate and often covered with other types of trichomes.

#### Leaf anatomy and indumentum

**Figure 5.**
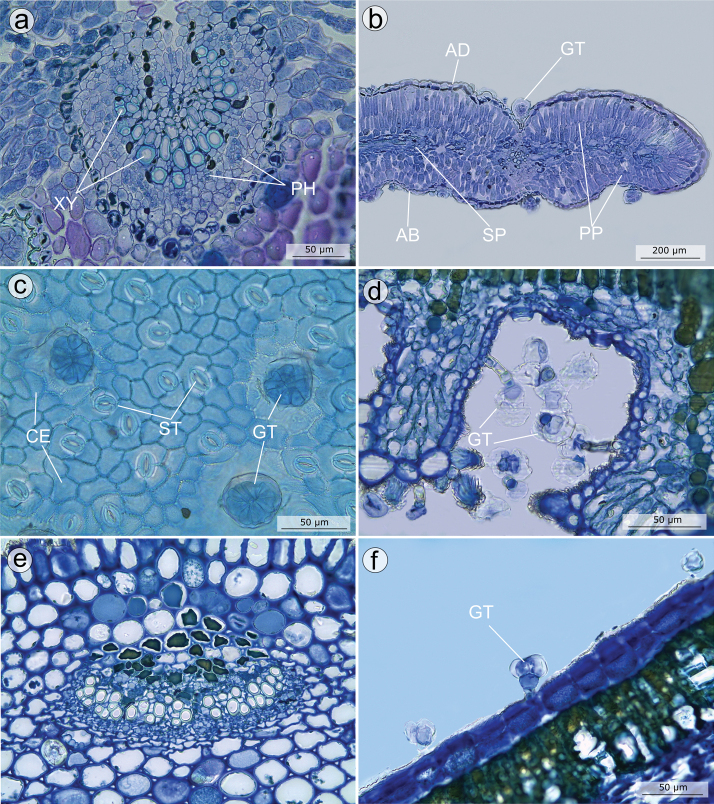
Anatomical features of leaves in the *Trembleya* s.s. clade of *Microlicia***A–C***Microliciaaltoparaisensis*: **A** cross section of the mid-vein, showing an amphicribral arch-shaped vascular bundle **B** cross section of the leaf, showing the isobilateral mesophyll, the wavy epidermis and a glandular trichome in a depression **C** Adaxial surface of the epidermis in frontal view, with stomata and glandular trichomes **D, E***Microliciachamissoana*: **D** leaf stomatal crypt in cross section, with glandular trichomes **E** petiole in cross section, showing an amphicribral vascular bundle **F***Microliciaflaviflora*, cross section of the leaf blade showing a reduced glandular trichome. AB: Abaxial surface; AD: Adaxial surface; CE: Common epidermal cells; GT: Glandular trichome; PP: Palisade parenchyma; SP: Spongy parenchyma; ST: Stomata. All photos by A.A.O. Carmo. Voucher specimens: **A–C** Pacifico & Bressan 380 (CAS, HUEM, SPF) **D, E** Pacifico & Carmo 154 (HUEM, UEC) **F** Mello-Silva et al. 509 (SPF).

Information on leaf anatomy is available for *M.altoparaisensis* (Pacifico et al. 2019), *M.calycina* (Pflaum 1897), *M.chamissoana* (Carmo et al. 2019, 2020), *M.laniflora* (Pflaum 1897; Silva et al. 2018), *M.parviflora* (Pflaum 1897; Somavilla and Graciano-Ribeiro 2011), *M.flaviflora* (Carmo et al. 2019, 2020), *M.pithyoides* (Pflaum 1897), *M.rosmarinoides* (Pflaum 1897) and *M.tridentata* (Pflaum 1897). A plate with photos of leaf anatomical characters is presented in Fig. 5 (A–F). Overall, the leaf epidermis is uniseriate with outer periclinal walls thicker than the other walls. The adaxial epidermal cells have straight to slightly sinuous walls in top view, while the abaxial epidermal cells have walls varying from straight to sinuous (Fig. 5C). *Microliciaaltoparaisensis* has amphistomatic leaves, while the remaining four species have hypostomatic leaves. Cuticle striation was reported for *M.chamissoana* and *M.flaviflora*. In cross-section, the petiole has an ovate or arched shape, 3–5 bicollateral or amphicribal vascular bundles (Fig. 5E) and collenchyma in the cortical region. The mesophyll varies from isobilateral (Fig. 5B) to dorsiventral or mixed, always with palisade parenchyma as the cortical tissue and the mid-rib projected. The shape of the mid-rib varies from arched to open arched, with bicollateral or amphicribral vascular bundles (Fig. 5A) and parenchyma and/or collenchyma at the cortical region. Sclereids and crystalliferous cells generally occur in the petiole, mesophyll and mid-rib. In *M.flaviflora* and *M.chamissoana*, the veinlets have imperfect areoles and terminal sclereids and the apical venation is not ramified (fig. 5C in Carmo et al. 2019). In the marginal region, the venation is incomplete in *M.flaviflora* and arch-shaped in *M.chamissoana* (fig. 5E in Carmo et al. 2019).

**Figure 6.**
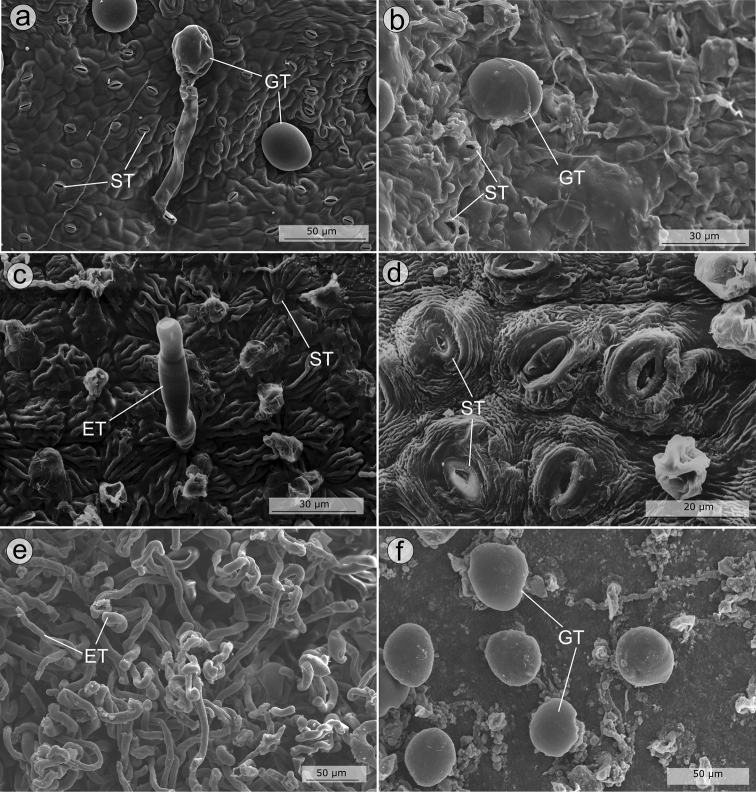
SEM images showing details of the leaf surface in the *Trembleya* s.s. clade of *Microlicia***A***Microliciaaltoparaisensis*, glandular trichomes and stomata on the leaf abaxial surface **B***Microliciacalycina*, glandular trichomes and stomata on the leaf abaxial surface **C***Microliciachamissoana*, eglandular trichomes and stomata on the leaf abaxial surface **D***Microliciaflaviflora*, stomata on the leaf abaxial surface **E, F***Microlicialaniflora*: **E** eglandular woolly trichomes on the leaf abaxial surface **F** glandular trichomes on the leaf adaxial surface. ET: Eglandular trichomes; GT: Glandular trichomes; ST: Stomata. Voucher specimens: **A** Pacifico & Bressan 380 (CAS, HUEM, SPF) **B** Pacifico 290 (CAS, SPF) **C** Pacifico & Carmo 154 (HUEM, UEC) **D** Mello-Silva et al. 509 (SPF) **E, F** Almeda et al. 9179 (CAS, UEC).

**Figure 7.**
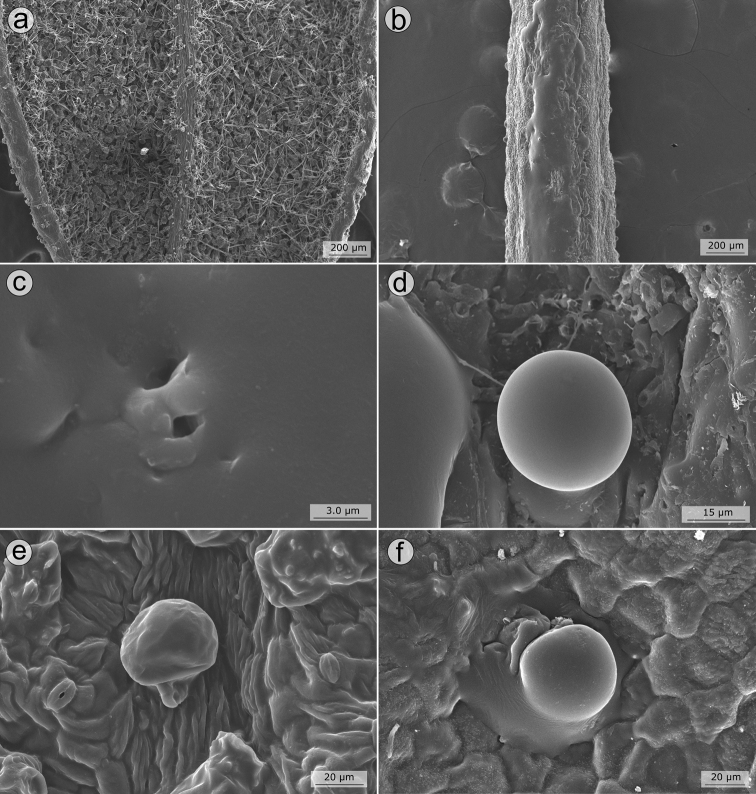
SEM images of leaves in the *Trembleya* s.s. clade of *Microlicia***A***Microliciaparviflora*, abaxial surface in frontal view **B–D***Microliciapithyoides*: **B** abaxial surface in frontal view **C** pore on the leaf abaxial surface **D** glandular trichome on the leaf adaxial surface **E***Microliciatrembleyiformis*, glandular trichome on the abaxial surface **F***Microliciatridentata*, detail of a glandular trichome on the adaxial surface. All photos by R. Pacifico. Voucher specimens: **A** Almeda et al. 8483 (CAS, HUFU, NY) **B, C** Pacifico 295 (CAS, HUEM, SPF) **E** Romero & Nakajima 3593 (HUFU, UEC) **F** Pacifico 290 (CAS, HUEM, SPF).

All species of the *Trembleya* s.s. clade have some type of trichome on the leaves, branchlets and abaxial leaf surfaces. Scanning Electronic Microscopy images showing details of the leaf surface in the clade are presented in Figs 6–7. The common glandular-punctate indumentum consists of a set of glandular trichomes with a multicellular head and a stalk that is too short to be seen with a typical stereomicroscope (Carmo et al. 2019; Figs 5F, 6A, B, F, 7D–F). This type of glandular trichome was initially described by Pflaum (1897), but only illustrated in detail by Mentink and Baas (1992; as “bladder-like glandular hairs”). Similar trichomes were commonly referred to as “sessile glands”, “sessile glandular trichomes” or “unforrowed sessile glands” (for example, by Wurdack 1986) across the taxonomic literature. As these trichomes are not sessile, they have been treated as typical glandular trichomes in anatomical studies (Reis et al. 2005; Cassiano et al. 2010; Romero and Castro 2014; Silva et al. 2018; Carmo et al. 2019, 2020). In a more detailed investigation, Carmo et al. (2019) proposed new names for trichomes and epidermal appendages, based on the structure of the stalk, head and cuticle. Carmo et al. (2019) included two species of the *Trembleya* s.s. clade in their study (*M.chamissoana* and *M.flaviflora*). Together with the anatomical descriptions and figures provided by Somavilla and Graciano-Ribeiro (2011), Silva et al. (2018) and Pacifico et al. (2019), the following types of trichomes were reported for the *Trembleya* s.s. clade:

1. Reduced glandular trichomes with apex consisting of 2–4 globular cells, distended cuticle and pluricellular stalk, in *M.chamissoana* and *M.flaviflora* (Carmo et al. 2019);
2. Uniseriate elongated eglandular trichomes, narrow at the base and truncated at the apex, with circular depressions in *M.chamissoana* (Carmo et al. 2019; Fig. 6C);
3. Short-stalked glandular trichomes with pluricellular clavate apex, with or without a distended cuticle, in *M.parviflora* (Somavilla and Graciano-Ribeiro 2011) and *M.altoparaisensis* (Pacifico et al. 2019; Fig. 6A);
4. Woolly eglandular trichomes in *M.laniflora* (Silva et al. 2018; Fig. 6E).

#### Inflorescence

**Figure 8.**
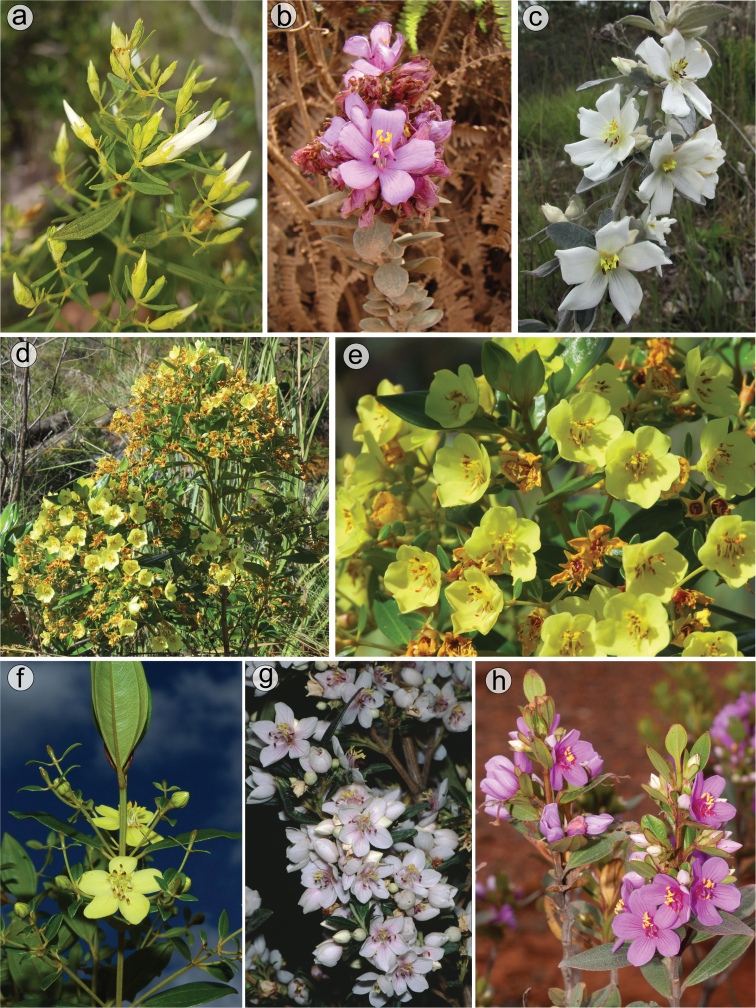
Inflorescences in the *Trembleya* s.s. clade of *Microlicia***A***M.altoparaisensis***B***M.chamissoana***C***M.laniflora***D–F***M.flaviflora***G***M.parviflora***H***M.tridentata*. Photos: **A** by V. E. Bressan **B** by F.A.O. Silveira **C** by L. Pedrosa **D, E** by R. Pacifico **F** by A.V. Scatigna **E** by F. Almeda **F** by F.A. Michelangeli.

Given the diversity of inflorescence types and the presence of many similar structures which are not guaranteed homologous, efforts have been made to establish natural systems for the flower-bearing parts of the Angiosperms (e.g. Briggs and Johnson 1979; Weberling 1988; Prusinkiewicz et al. 2007; Kirchoff and Claßen-Bockhoff 2013). Inflorescences found in Lavoisiereae always have a terminal flower, which means that they are determinate, i.e. monotelic *sensu*Weberling (1988) and anthotelic *sensu*Briggs and Johnson (1979). Overall, inflorescences in the *Trembleya* s.s. clade are similar in structure to those of the *Lavoisiera* clade, which were described in detail by Martins and Almeda (2017). In both clades, the inflorescences always consist of a proliferation or reduction, based on a 3-flowered dichasial unit, with opposite branching like that of sterile branches (Fig. 8). The dichasial unit consists of a terminal flower subtended by a pair of modified leaves (bracts). Two lateral branches originate from the axils of the bracts to produce lateral flowers, which are, in turn, subtended by a pair of bracteoles. The proliferation of this pattern produces biparous cymes in a compound inflorescence. In some cases, only one lateral branch develops from the axil of a bract. This pattern is associated with production of uniparous cymes at the distal portion of the inflorescence and is here reported for a few species.

In the *Trembleya* s.s. clade, seven species have simple or compound dichasia (*M.altoparaisensis*, *M.calycina*, *M.chamissoana*, *M.flaviflora*, *M.laniflora*, *M.parviflora* and *M.tridentata*) and the remaining four have inflorescences reduced to solitary flowers at the apical region of the branches (*M.pentagona*, *M.pithyoides*, *M.rosmarinoides* and *M.trembleyiformis*). Floral pedicels are usually evident and measure 0.3–4 mm long. These are inconspicuous to 0.2 mm long only in *M.altoparaisensis*. Like some species of the *Lavoisiera* clade (Martins and Almeda 2017), a tendency of inflorescence reduction to congested clusters occurs in *M.chamissoana* and *M.laniflora* (Fig. 8B–C). The degree of inflorescence development, as well as the shape, size and venation of the bracteoles, are characters of taxonomic significance at the species level.

#### Hypanthium and calyx

The hypanthium that envelops the superior ovaries is campanulate to urceolate and its length varies between 1.7 and 6.5 mm. The external surface is light green, reddish or golden and has (8)10(12) longitudinal vascular ribs that terminate apically in a circular ring where the hypanthium and calyx lobes meet. In Melastomataceae, this region is usually referred to as the torus (Wurdack 1953; Almeda 1978; Martins and Almeda 2017). In two species (*M.tridentata* and *M.pentagona*), the hypanthium could be described as glabrous, although it is minutely granulose and covered by a glutinous substance. In four species, the external surface is only glandular-punctate (*M.altoparaisensis*, *M.calycina*, *M.pithyoides* and *M.rosmarinoides*), while, in the other three, eglandular or gland-tipped trichomes usually occur associated with the glandular-punctate indumentum (*M.chamissoana*, *M.parviflora* and *M.trembleyiformis*). An atypical indumentum is found in *M.laniflora*, which has the external surface of the hypanthium totally hidden by a dense lanose indumentum of eglandular trichomes and in *M.parviflora*, which has a hypanthium that is usually pruinose, eventually sparsely to densely covered with gland-tipped trichomes or rarely covered with what appear to be rigid hyaline eglandular trichomes.

The calyx lobes that develop above the torus are united at the base and that union forms the calyx tube, which, in the *Trembleya* s.s. clade, varies in length between 0.1 and 0.7 mm. The calyx lobes are either oblong, triangular, narrowly triangular or subulate, with the apex acute or acuminate and between 0.7 and 9.7 mm long. The indumentum of both calyx tube and calyx lobes is usually similar to that of the hypanthium. Differences in the shape, size and indumentum of the calyx lobes are generally useful diagnostic characters at the species level in this clade.

#### Corolla

**Figure 9.**
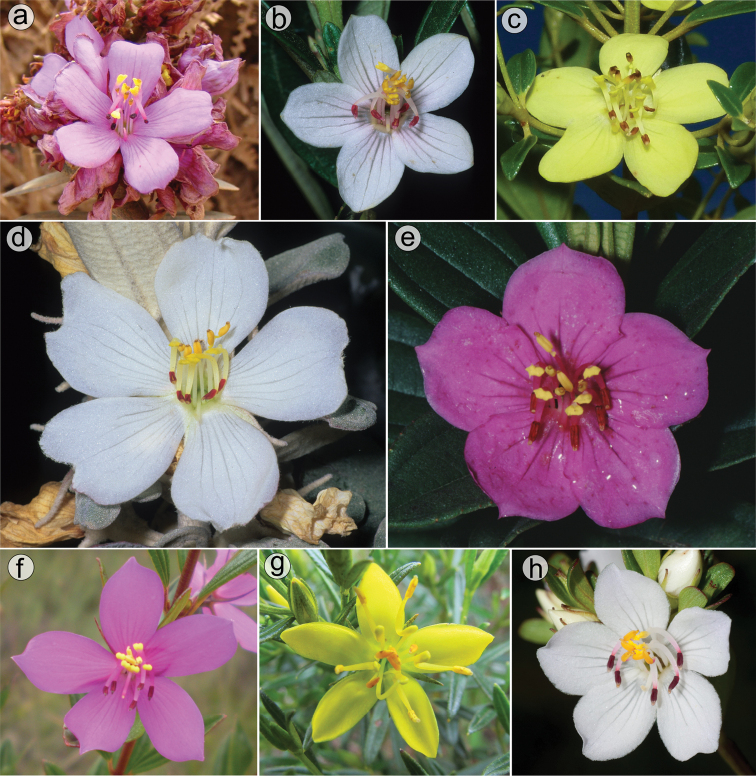
Diversity of flowers in the *Trembleya* s.s. clade of *Microlicia***A***M.chamissoana***B***M.parviflora***C***M.flaviflora***D***M.laniflora***E, F***M.pentagona***G***M.rosmarinoides***H***M.tridentata*. Photos: **A** by F.A.O. Silveira **B** and **D–F** by F. Almeda **C** by A.V. Scatigna **G** by L. Pedrosa **H** by F.A. Michelangeli.

Flowers are generally 5-merous (Fig. 9) with petals convolute in bud. Rarely, one or a few 4-merous or 6-merous flowers are found in inflorescences predominantly with 5-merous flowers. The corolla is always radially symmetrical at anthesis. The petals are free, attached to the torus alternating with calyx lobes and opposing the smaller set of stamens. They vary between 4.5 and 26.0 mm in length, exceeding 20 mm only in *M.laniflora*. The shape is obovate with the apex rounded or acute, varying in colour from white (*M.altoparaisensis*, *M.laniflora*) to magenta (*M.calycina*, *M.chamissoana*, *M.pentagona*, *M.pithyoides*, *M.trembleyiformis*, *M.tridentata*) or yellow (*M.flaviflora*, *M.rosmarinoides*) (Fig. 9). In *M.parviflora*, both magenta and white petals occur in distinct populations. These petals are more commonly described as white with pink stains. A rare form of *M.laniflora* with pink-flushed petals was once reported from Serra do Cipó, Minas Gerais. Both surfaces of the petals are generally glabrous and the margin varies from entire to glandular-punctate (*M.chamissoana*) or ciliate (*M.laniflora*, sometimes in *M.parviflora*).

#### Androecium

**Figure 10.**
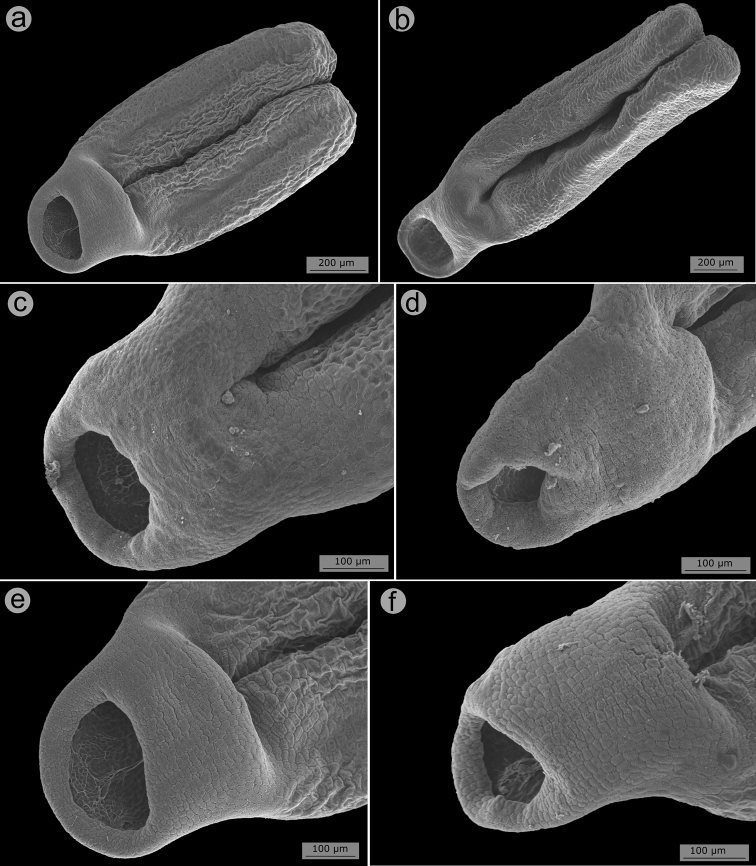
SEM images of anthers and anther rostra in the *Trembleya* s.s. clade of *Microlicia***A***Microliciaparviflora*, anther of an antesepalous stamen **B***Microliciatridentata*, anther of antesepalous stamen **C***Microliciaflaviflora*, anther rostrum of an antesepalous stamen **D***Microliciaflaviflora*, anther rostrum of antepetalous stamen **E***Microliciaparviflora*, anther rostrum of an antesepalous stamen **F***Microliciaparviflora*, anther rostrum of an antepetalous stamen. Voucher specimens: **A, E, F** Pirani et al. CFSC 12361 (SPF) **B** Pacifico & Bressan 290 (CAS, HUEM) **C, D** Hatschbach et al. 54239 (CAS, INPA, MBM).

**Figure 11.**
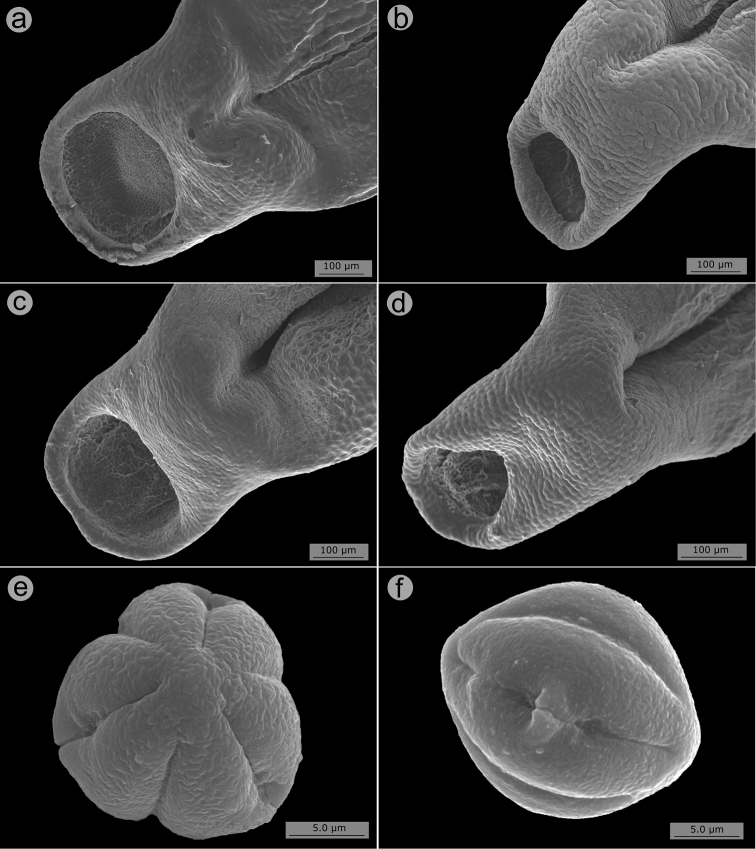
SEM images of anther rostra and pollen grains in the *Trembleya* s.s. clade of *Microlicia***A***Microliciapentagona*, anther rostrum of an antesepalous stamen **B***Microliciapentagona*, anther rostrum of an antepetalous stamen **C***Microliciatridentata*, anther rostrum of an antesepalous stamen **D***Microliciatridentata*, anther rostrum of an antepetalous stamen **E, F***Microliciapithyoides*, pollen grains. Voucher specimens: **A, B** Irwin et al. 20486 (CAS, MO, NY, US) **C, D** Pacifico & Bressan 290 (CAS, HUEM) **E, F** Pacifico 295 (CAS, HUEM, SPF).

Flowers are diplostemonous and, therefore, generally have ten stamens, which are organised in an antesepalous whorl of larger stamens and an antepetalous whorl of smaller stamens. In all but one species of the clade (*M.altoparaisensis*), the pedoconnectives of the antepetalous stamens are much shorter than those of the antepetalous stamens, forming a yellow ventral appendage that measures about 0.1(–0.5) mm and is often inconspicuous. The pedoconnectives of the antesepalous stamens are generally well-developed, forming yellow ventral appendages (0.1–)0.7–3.0 mm long that are usually emarginate at the apex. In the majority of species, the antesepalous stamens have purple to vinaceous anthers in strong contrast to the yellow anthers of the antepetalous whorl. The exception is *M.altoparaisensis*, which has subisomorphic stamens with all anthers yellow. The filaments are filiform, glabrous, measuring 1.5–6.3 mm long, usually similar in colour to the petals. The anthers are oblong (Fig. 10A, B), 0.7–3.8 mm in length (excluding rostra), with a smooth external surface and 2-celled tetrasporangiate thecae. Each anther tapers to a rostrum measuring 0.2–0.5(–0.7) mm long with a ventrally inclined apical pore (Figs 10C–F, 11A–D). A remarkable difference between the two sets of stamens is that the anther pores of the antepetalous whorl are always narrower than those of the antesepalous set. This distinction is usually only evident when anthers are examined with Scanning Electron Microscopy (Figs 10C–F, 11A–D). Overall, in this clade, the androecium is radially symmetrical at bud and bilaterally symmetrical at anthesis (see Fig. 9). For notes on reproductive strategies, see the topic “Reproductive Biology, Pollination and Phenology.”

#### Pollen

Pollen grains in Melastomataceae are small, generally tricolporate, radially symmetrical and isopolar (Patel et al. 1984; Renner 1993). Apparently of little taxonomic use, these characters have rarely been described in taxonomic studies of the family (Almeda and Robinson 2011; Alvear and Almeda 2019). Based on SEM photos of four species of the *Trembleya* s.s. clade taken during this study (*T.altoparaisensis*, *M.calycina*, *M.chamissoana* and *M.pithyoides*), the pollen grain structure of the clade is apparently not informative taxonomically and matches the descriptions provided in the above-cited references. SEM images of pollen grains of *M.pithyoides* are presented in Fig. 11E, F.

#### Gynoecium and capsule

The ovary is always superior, glabrous and completely enclosed by the hypanthium. Ovaries are 5-locular in most species, 3(–4)-locular only in *M.altoparaisensis* and vary from 3–5-locular only in *M.trembleyiformis*. These differences in ovary locule number are additional characters that can be used for species recognition. The ovaries are ovoid to globose or cylindrical, measuring between 0.9 and 4.1 mm in length. Placentation is axile with numerous tiny anatropous ovules attached to subpeltate placental intrusions. The style is filiform (3–10 mm long) and incurved distally to sigmoid at anthesis, glabrous, similar in colour to the petals and the stigma is punctiform to truncate.

**Figure 12.**
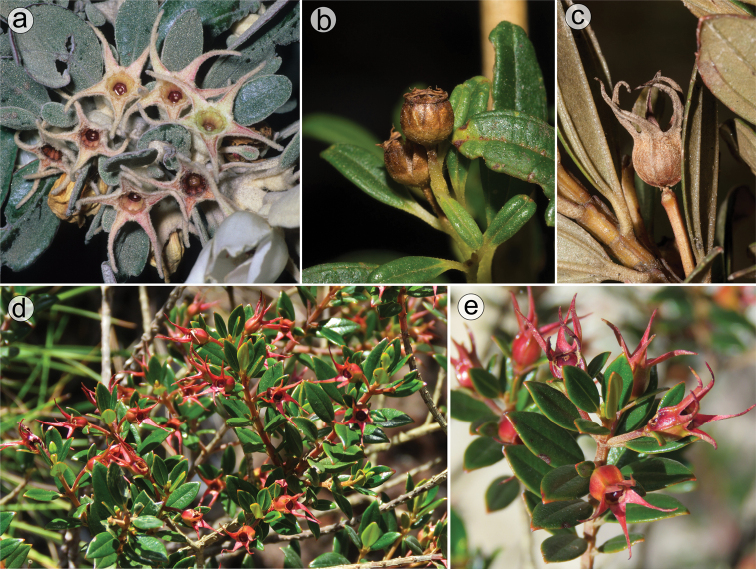
Capsules and infructescences in the *Trembleya* s.s. clade of *Microlicia***A***Microlicialaniflora*, infructescence **B***Microliciaparviflora*, capsule enveloped by the hypanthium **C–E***Microliciapentagona*: **C** capsule enveloped by the hypanthium **D** infructescence **E** capsules enveloped by reddish mature hypanthia. Photos: **A** by F. Almeda **B, C** by F.A. Michelangeli **E, F** by R. Pacifico.

The brownish capsules are loculicidal measuring 2.3–8.0 mm in length, initially enveloped by the hypanthium and calyx that rupture and flake away tardily with age (Fig. 12). The shape of the capsule is ovoid to globose. The enveloping hypanthium is little or markedly constricted at the apex (torus). The capsules dehisce from the apex to the base (basipetal) into 3–5 valves with a deciduous columella. Both calyx tubes (0.2–3.1 mm long) and calyx lobes (1.2–11.5 mm long) may become elongated in fruit. The apex of the mature capsule never exceeds the torus, like it does in several species of *Microlicia* s.s. In fruit, the calyx lobes become sharply thicker only in *M.pentagona* (Fig. 12C–E).

#### Seed structure and germination

**Figure 13.**
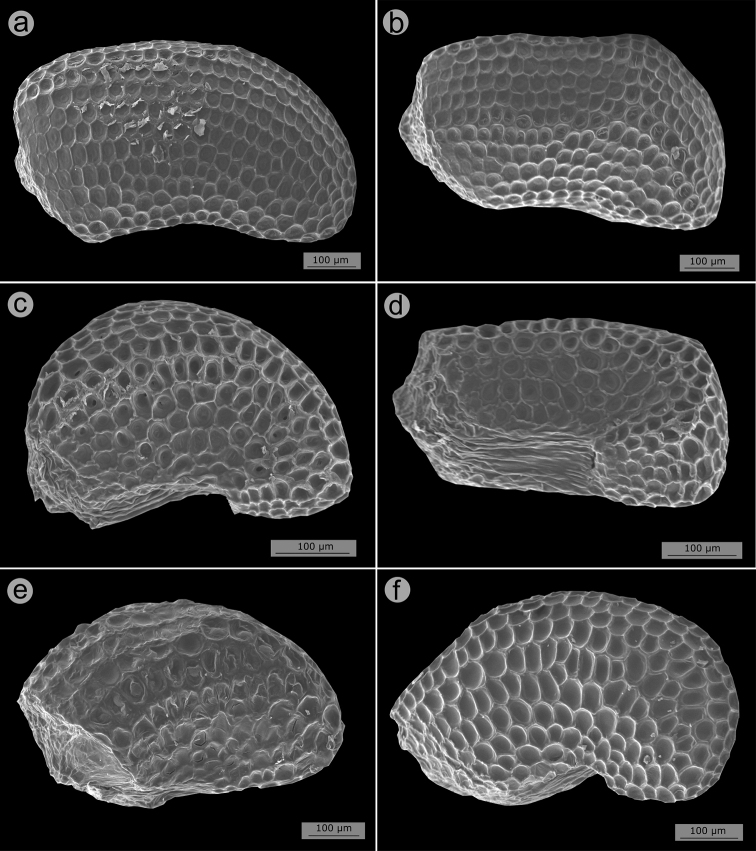
SEM images of seeds in the *Trembleya* s.s. clade of *Microlicia***A***Microliciacalycina***B***Microlicialaniflora***C, D***Microliciaparviflora***E***Microliciapentagona***F***Microliciarosmarinoides*. All photos by R. Pacifico. Voucher specimens: **A** Pacifico & Bressan 296 (CAS, HUEM, SPF) **B** Almeda et al. 7726 (CAS, UEC) **C, D** Almeda et al. 8483 (CAS, HUFU, NY) **E** Irwin et al. 20178 (CAS, MO, NY, UEC, US) **F** Occhioni et al. s.n. (US [US001900109]).

Seed micromorphological characters have been traditionally used in the tribal and generic classification of Melastomataceae (Cogniaux 1883–1888; Baumgratz 1983; Fritsch et al. 2004; Ocampo et al. 2014; Goldenberg et al. 2015; Penneys et al. 2022). In fact, one of the synapomorphies attributed to the Lavoisiereae is the presence of ellipsoid to reniform seeds with a reticulate-foveolate testa (Almeda and Martins 2001; Fritsch et al. 2004; Rocha et al. 2016; Martins and Almeda 2017; Pacifico and Almeda 2022). The use of seed morphology to recognise species from closely-related species groups is limited in Melastomataceae, although some investigations have revealed promising seed characters that could be used together with macromorphology for species circumscriptions (Ocampo and Almeda 2013; Martins and Almeda 2017; Ocampo et al. 2022). In the *Trembleya* s.s. clade, the six species for which mature seeds were available are similar in shape and testa sculpturing and typical of Lavoisiereae (Fig. 13). Overall, they are slightly curved to reniform with the raphal zone oblong, occupying ca. 30–70% of the total length of the seed (total length = 0.2–0.8 mm) and the testa cells are arranged in an aligned pattern.

Information on seed germination in the clade is limited to studies of *M.parviflora* and *M.laniflora*. According to Silveira et al. (2012), seeds of these two species are non-dormant. For *M.laniflora*, the optimum temperature range for germination is 20–25 °C, which matches the onset of the rainy season (Rodrigues and Silveira 2013). Seeds of *M.laniflora* are viable after 42 months of storage (Rodrigues and Silveira 2013) and for being easily buried, they are likely easily incorporated into soil seed banks (Rodrigues and Silveira 2013). According to the investigations by Giotto (2015), seeds of *M.parviflora* usually take 5–14 days to germinate and germination occurs more quickly when the seeds are exposed to variations in temperature. Germination is higher for seeds that are stored for more than a month after collection, and they continue to be viable for at least 15 months after being collected (Giotto 2015).

#### Reproductive biology and pollination

Flowers are hermaphroditic with a strongly dimorphic (rarely subisomorphic) androecium and poricidal anthers. Plants of this clade provide pollen as a primary resource to reward pollinators and apparently developed floral traits to protect the pollen, select pollinating agents and precisely deposit pollen on the body of the pollinator. These characteristics are usually understood as strategies to deal with the “pollen dilemma”, in which pollen grains fulfil two main functions, i.e. to transport the male gamete and to reward flower visitors with resource supply (Westerkamp 1996; Luo et al. 2008). In Melastomataceae, nectar production is apparently restricted to a few genera (see Varassin et al. 2008) and has never been reported in Lavoisiereae. During anthesis, the large anthers are generally positioned almost parallel to each other, serving as a “landing platform”, while the smaller anthers are more erect and positioned to serve as the feeding set; a clustering of the yellow anthers of the antepetalous stamens, together with the yellow connective appendages of the antesepalous stamens, apparently acts as a visual sign to pollinators in most species. As usual in Melastomataceae (Renner 1989; Dellinger et al. 2022), the pollen is collected by bees that perform buzz pollination. The bees position themselves over the androecium and perform rapid distortions of the thoracic box and produce a characteristic buzzing sound (Renner 1989).

By having flowers with vibrant colours and diurnal anthesis, most species of Lavoisiereae seem to meet the requirements of the classic melittophily syndrome (Faegri and van Der Pijl 1979). The exception is *M.laniflora*, whose white flowers are the largest in the clade and open at night and dusk (Soares and Morellato 2018). The reproductive success of *M.laniflora* seems to be highly dependent upon large bees of the genus *Xylocopa* that perform crepuscular foraging (Soares and Morellato 2018). Flowers of *M.laniflora* are self-incompatible and depend on cross-pollination; this behaviour suggests that pollinator-mediated interbreeding is a strategy to promote genetic variability of populations naturally isolated on rocky outcrops (Soares and Morellato 2018).

There is no evidence of apomixis in *M.laniflora* (Soares and Morellato 2018). The widely-distributed *M.parviflora* has been reported as a facultative apomict (Silva 2001) or non-apomictic (Maia et al. 2016) and self-compatible (Silva 2001; Maia et al. 2016). Still, Maia et al. (2016) reported reduced speed of pollen tube growth in self-pollination treatments. Both staminal whorls have fertile pollen (Santos et al. 2012). Functional dimorphism of stamens in *M.parviflora* is still possible because germination for each type of pollen has not been tested (Santos et al. 2012). Natural vegetative reproduction, probably from rhizomes, was reported by Campos (2005) and Baumgratz et al. (2007). The occasional apomictic behaviour of *M.parviflora* is consistent with the general pattern found in Melastomaceae, a family in which apomictic species generally have wider distributions than non-apomictic taxa (Santos et al. 2012; Caetano and Oliveira 2022). Silva et al. (2012) reported that *M.parviflora* is one of the native plant species most visited by the pollinating bee *Eulaemanigrita* in an agroecosystem in Uberlândia (Minas Gerais), indicating that this species performs significant ecosystem services in that region.

**Figure 14.**
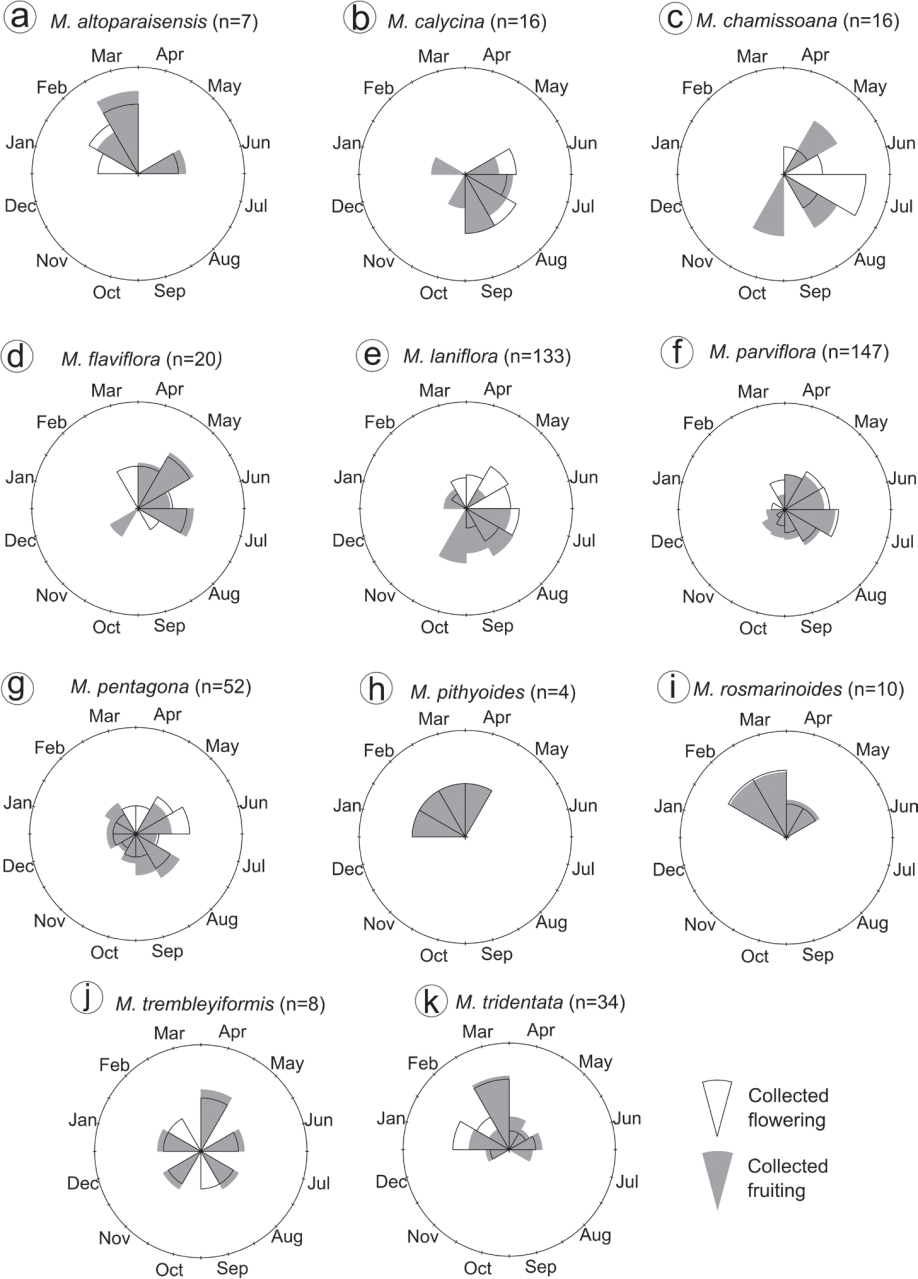
Circular histograms of phenology for species of the *Trembleya* s.s. clade of *Microlicia*. Information on flowering and fruiting periods were obtained from herbarium specimens.

Populations of *Microliciaparviflora* from different localities may be distinct in phenological behaviour, with annual or episodic flowering and continuous or annual fruiting; this species is commonly reported from both typical campo rupestre and gallery forests (Brito et al. 2017). Overall, a tendency to produce flowers during the dry season and seed dispersal at the beginning of the rainy season has been reported in phenological studies for shrubby Melastomataceae from campo rupestre (Le Stradic 2012) and especially in *M.laniflora* (Rodrigues and Silveira 2013; Soares and Morellato 2018). This tendency is believed to be an adaptation for effective pollination and seed dispersal (Salazar et al. 2011; Silveira et al. 2012; Soares and Morellato 2018). Our histograms of flowering and fruiting periods, based on herbarium specimens (Fig. 14), agree with what has been described for *M.laniflora* and suggest a similar tendency for *M.calycina*, *M.chamissoana* and *M.flaviflora*. Three other species (*M.altoparaisensis*, *M.pithyoides* and *M.rosmarinoides*) could have a distinct phenological pattern, flowering and fruiting mainly during the wet season (Fig. 14A, H, I), although field observations are necessary to confirm this hypothesis. *Microliciapentagona* and *M.trembleyiformis* apparently produce flowers and fruits more evenly throughout the year (Fig. 14G, J). Additional phenological studies of this clade and the Lavoisiereae, in general, are desirable.

#### Chemical characters

Knowledge of secondary metabolites of Lavoisiereae is largely restricted to the work by Bomfim-Patrício et al. (2001), who provided leaf flavonoid profiles for 33 species of the tribe, including four species from the *Trembleya* s.s. clade (*M.chamissoana*, *M.laniflora*, *M.parviflora* and *M.pentagona*). They reported 116 flavonoids for the tribe with a predominance of flavonol glycosides. Species of the *Trembleya* s.s. clade do not form a coherent group with respect to a flavonoid profile. A predominance of flavonols in *M.laniflora* and *M.parviflora* resembles *Microlicia* s.s., while 6-methoxylation found in *M.chamissoana* and *M.pentagona* resembles species of the *Lavoisiera* clade (Bomfim-Patrício et al. 2001).

Information on histochemical tests is available only for *M.parviflora* and *M.laniflora*. Regarding the first, phenolic compounds were reported from the endodermis and parenchyma (Somavilla and Graciano-Ribeiro 2011), histochemical tests performed on the exodermis cells of the roots were positive for suberin and negative for lignin (Somavilla and Graciano-Ribeiro 2012), oil droplets were found in the mesophyl of the leaves (Somavilla and Graciano-Ribeiro 2011), coumarins, steroids, triterpenes, flavonoids and tannins were also reported, with the following major compounds present in the volatile oils: α-terpineol, α-pinene, β-pinene, sabinene, acetoxyeudesman-4-α-ol and 2,4a-8,8-tetramethyldecahydrocyclopropanaphtalene (Farias et al. 2018). Alkaloids probably occur in *M.parviflora* as well (Raffauf 1996). In *M.laniflora*, phenolic compounds were reported from the leaf palisade parenchyma, collenchyma, vascular bundle sheath (Silva et al. 2018), as well as saponins, triterpenes, tannins, flavonoids (Ventura et al. 2007a) and a flavanone (Ventura et al. 2007b).

#### Chromosome numbers

Only about 10% of the species of Melastomataceae are known cytologically, but some patterns of chromosomal evolution are evident. Chromosome number stasis at the diploid level and recurrent cycles of polyploidy and dysploidy are common (Almeda 2013; Almeda and Penneys 2022). The base number attributed to the family is *x* = 12 (Almeda 2013). Lavoisiereae is quite uniform with *x* = 12 and tetraploidy based on that number, while *Rhynchanthera* differs in having *x* = 10 and tetraploidy on that base number (Almeda and Penneys 2022). In the *Trembleya* s.s. clade, chromosome counts are available only for two species. The diploid *M.parviflora* has *n* = 12 and the tetraploid *M.pentagona* has *n* = 24 (Almeda and Penneys 2022).

#### Geographic distribution, biogeography and endemism

**Figure 15.**
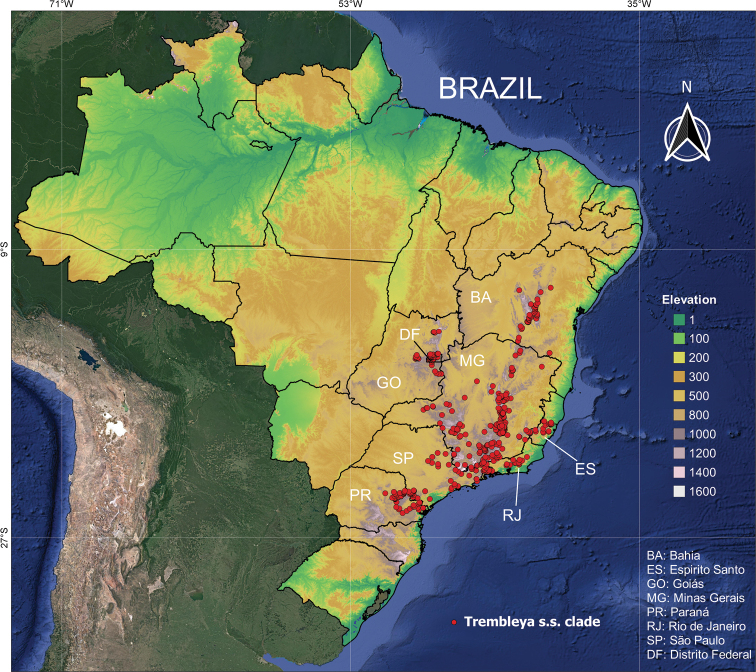
Geographic distribution of the *Trembleya* s.s. clade of *Microlicia* in Brazil.

**Figure 16.**
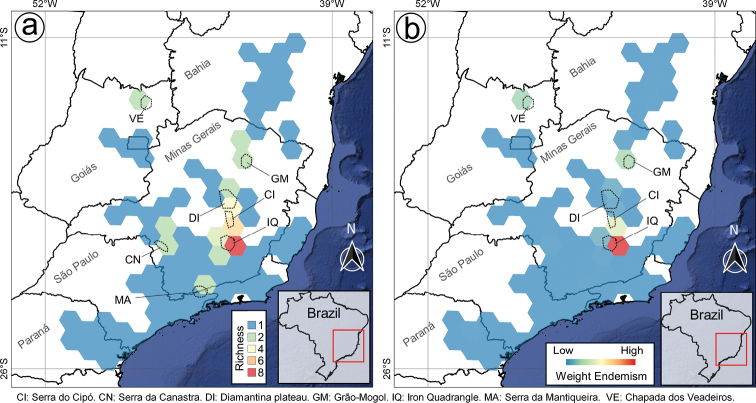
Richness and weight endemism maps of the *Trembleya* s.s. clade of *Microlicia***A** species richness map **B** species weight endemism map.

The *Trembleya* s.s. clade is restricted to Brazil (Fig. 15). Both richness and weight endemism are concentrated in the Minas Gerais State (southern Cadeia do Espinhaço), where 10 species occur (Fig. 16). The only two other States with more than one species are Goiás (*M.altoparaisensis* and *M.parviflora*) and Rio de Janeiro (*M.parviflora* and *M.trembleyiformis*). *Microliciaparviflora* has the widest distribution in the clade and is reported for the states of Bahia, Minas Gerais, Goiás, Distrito Federal, Espírito Santo, Rio de Janeiro, São Paulo and Paraná.

**Figure 17.**
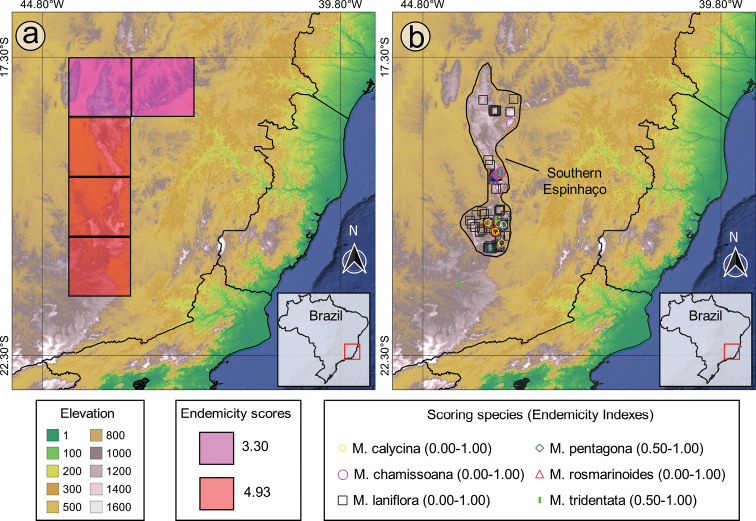
The area of endemism of the *Trembleya* s.s. clade of *Microlicia*, based on Endemicity Analysis **A** grid cells corresponding to the Southern Espinhaço area of endemism **B** the Southern Espinhaço area of endemism manually drawn, based on distribution of species of *Trembleya* s.s. clade of *Microlicia*.

Endemicity analyses detected one consensus area of endemism for the *Trembleya* s.s. clade, which resulted from the merging of four individual areas of endemism (Fig. 17). The consensus area consisted of five cells and is a good match for the Southern Espinhaço biogeographic Province (Colli‐Silva et al. 2019), which is also a major area of endemism for the Lavoisiereae (Pacifico et al. 2020). The highest endemicity score of the consensus area is 4.93 (at the southern region) and the lowest endemicity score 3.30 (at the northern region). *Microliciacalycina*, *M.chamissoana*, *M.laniflora*, *M.pentagona*, *M.rosmarinoides* and *M.tridentata* were recovered as endemic species and their endemicity scores are presented in Fig. 17.

The region with highest richness and weight endemism of the clade is the Iron Quadrangle (Quadrilátero Ferrífero), located in the Municipalities of Barão dos Cocais, Belo Horizonte, Brumadinho, Caeté, Catas Altas, Congonhas, Itabirito, Mariana, Nova Lima, Ouro Branco, Ouro Preto, Raposos, Rio Acima, Sabará and Santa Bárbara (Minas Gerais State). The Iron Quadrangle is one of the world’s largest mineral provinces, consisting of rocks associated with the Archean and Paleoproterozoic periods and includes mountain ranges, such as the Serra de Ouro Branco, Serra de Ouro Preto, Serra da Moeda, Serra da Piedade, Serra do Gandarela, Sera do Garimpo, Serra do Rola-Moça and Serra do Caraça (Ruchkys and Machado 2013). It has been recognised as a distinct biogeographic district associated with campo rupestre in the Southern Espinhaço Province (Colli‐Silva et al. 2019) and an area of endemism for the tribe Lavoisiereae (Pacifico et al. 2020). Eight species of the clade have been reported for the Iron Quadrangle region: *M.calycina*, *M.laniflora*, *M.parviflora*, *M.pentagona*, *M.pithyoides*, *M.rosmarinoides*, *M.tridentata* and *M.trembleyiformis*. Another relevant endemism centre of the clade is the Serra do Cipó, where four narrow endemics can be found (*M.chamissoana*, *M.laniflora*, *M.pentagona* and *M.tridentata*). Only two narrow endemics are not associated to the regions described above: *Microliciaflaviflora*, which is restricted to northern Minas Gerais, in Grão Mogol, Botumirim and Rio Pardo de Minas and *M.altoparaisensis*, restricted to Chapada dos Veadeiros, Goiás.

The richness and endemism of the *Trembleya* s.s. clade are concentrated in the campo rupestre, a biodiversity-rich mosaic of mountaintop vegetation where about 15% of Brazilian vascular plant species occur in an area smaller than 1% of its territory (Vasconcelos 2011; Alves et al. 2014; Fernandes 2016; Silveira et al. 2016). In Brazil, this azonal peinobiome is found mainly on the Cadeia do Espinhaço (also referred to as “Espinhaço Range”), along the States of Bahia and Minas Gerais and in the Brazilian Central Plateau in the State of Goiás (Giulietti et al. 1997; Vasconcelos 2011; Alves et al. 2014). Its vegetation occurs mostly on mountains from about 900–2000 m, on lithosols associated with outcrops of Precambrian quartzites, sandstones and ironstones (Giulietti et al. 1997; Vasconcelos 2011; Alves et al. 2014; Silveira et al. 2016). All species of the *Trembleya* s.s. clade occur on quartzitic soils (for example, at the Serra do Caraça). Three species were also reported from ironstone outcrops known as cangas (*M.laniflora*, *M.rosmarinoides* and *M.tridentata*). Some species have non-overlapping elevational ranges, suggesting an elevated specificity in their habitat preferences, for example, *M.chamissoana* (1154–1462 m), *M.rosmarinoides* (1609–1807 m) and *M.pithyoides* (1827–2072 m). The most widely distributed species, *M.parviflora*, also has the widest elevational range in the clade (560–2223 m), extending into the Cerrado Biome in savannahs and gallery forests.

The traditional hypothesis for plant diversification in campo rupestre postulates that repeated retraction-expansion events driven by Pleistocene climatic changes would have worked as an evolutionary pump, creating a scenario where mountaintops functioned as long-term refuges and maintained lineages despite climatic alterations (Conceição et al. 2016). In this context, populations isolated on mountaintops would have been susceptible to genetic drift and inbreeding depression, which would have favoured speciation (Rapini et al. 2008). The evolutionary pump hypothesis has been recently challenged by Rapini et al. (2020), who argue that the long-term fragmentation, combined with recurrent extinctions and sporadic events of adaptive radiation, may provide a better explanation for the current diversity and endemism in campo rupestre, in accordance with the “escape-to-radiate model”. Another non-excluded hypothesis is that mountains with campo rupestre are old climatically-buffered infertile landscapes (OCBILs) (Hopper 2009; Silveira et al. 2016; Zappi et al. 2017), consisting of both a cradle of continuing diversification and a museum of ancient lineages (Silveira et al. 2016; Zappi et al. 2017; Vasconcelos et al. 2020).

*In situ* diversification apparently played an important role during the evolutionary history of the *Trembleya* s.s. clade, along with several other plant lineages (Fritsch et al. 2004; Pacifico et al. 2020; Vasconcelos et al. 2020). Major diversification of *Microlicia* s.l. may have occurred during the Pliocene (Fritsch et al. 2004) associated with the acquisition of xeromorphic features, for example, fire-resistant woody xylopodia (Simon et al. 2009), physiologically dormant seeds (Silveira et al. 2012), petiole shortening, stomatal crypts (Fig. 5D), reduction in leaf blade area, densification of palisade parenchyma, thickening of the outer periclinal wall of the epidermal cells (Carmo et al. 2020) and aluminium accumulation mechanisms (Jansen et al. 2002). These are all possible innovations which could have favoured a shift from mesophytic to xerophytic environments and the occupation of the diverse microhabitats found in campo rupestre (Fritsch et al. 2004; Silveira et al. 2012; Carmo et al. 2020).

#### Conservation

Overall, vascular plant lineages restricted to campo rupestre are highly vulnerable to disturbances and their conservation deserves special attention (Conceição et al. 2016; Silveira et al. 2016). Given their high phylogenetic conservatism, these lineages are not expected to adapt quickly to new conditions and are usually unable to colonise new habitats (Conceição et al. 2016; Silveira et al. 2016). Additionally, most of them would be very unlikely to reach distant suitable areas because of their low dispersal capability (Conceição et al. 2016; Silveira et al. 2016).

The historical stability of campo rupestre vegetation in the face of climatic changes is still under debate (Antonelli et al. 2010; Bitencourt et al. 2016; Conceição et al. 2016; Rapini et al. 2020). According to the OCBIL theory, mountaintops with campo rupestre have been buffered for a long time from climatic alterations and rapid changes could greatly reduce their biodiversity (Hopper 2009; Silveira et al. 2016). In global warming scenarios, Bitencourt et al. (2016) estimated that the Espinhaço Range may lose up to 56% of its plant diversity and 97% of microendemic species by 2080.

Most species of the *Trembleya* s.s. clade have narrow distributions limited to one or a few mountains and several of them also have narrow elevational ranges. The Brazilian Government already recognises three species of this clade as Endangered (EN), i.e. *M.calycina*, *M.chamissoana* and *M.flaviflora* and one as Critically Endangered (CR), *M.pithyoides* (Brasília 2014). In this study, based on AOO and EOO values and criterion B of IUCN (2019), our informal conservation status recommendations agree with those assigned to these species by the Brazilian Government. We also recommend that four additional species should be recognised as Endangered (EN), *M.altoparaisensis*, *M.pentagona*, *M.rosmarinoides* and *M.tridentata*, *M.laniflora* should be considered as Vulnerable (VU) and *M.parviflora* and *M.trembleyiformis* as Least Concern (LC).

## ﻿Systematic treatment

### ﻿ Trembleya s.s. clade of Microlicia

Taxon classificationPlantaeMyrtalesMelastomataceae

DA97B095-DD7D-579E-82DE-BA918CD6AB37

#### Diagnosis.

Perennial shrubs or treelets. Leaves petiolate, not imbricate, not keeled, the adaxial surface glandular-punctate to glabrescent, venation basal acrodromous, impressed on the adaxial surface and prominent on the abaxial surface, consisting of amphicribral or bicollateral vascular bundles. Flowers usually 5-merous, diplostemonous, pedicellate, subtended by a pair of bracteoles. Hypanthia not fused to the ovary, lacking a crown of trichomes at the apex. Stamens strongly dimorphic or subisomorphic, anthers 2-celled, tetrasporagiate. Ovaries superior, (3–4–)5-locular. Capsules dehiscent from the apex to the base, columella deciduous.

#### Description.

Perennial erect shrubs or small trees (0.1–)0.3–4 m tall, woody, sometimes densely branched. Distal branches quadrangular, usually light green (when fresh) and glutinous, glandular-punctate, sometimes granulose or pruinose, eventually covered with eglandular or gland-tipped trichomes, internodes 0.1–4.5 cm long, angles unwinged or narrowly winged, nodes thickened. Old branches terete, brownish and defoliating towards the base. Leaves decussate, petiolate, not imbricate, not keeled, papyraceous, chartaceaous or coriaceous, usually discoloured when dry. Petioles 0.3–17 mm long. Blades 0.4–11.7 cm long, 0.05–5 cm wide, oblong, lanceolate, elliptic, narrowly elliptic, ovate or linear, entire to slightly serrulate, sometimes entire along the basal half and serrulate on the upper half, rarely ciliate, lacking support tissue on the leaf margin. Adaxial surface green (when fresh), becoming pale green, pale brown, or darkened (when dry), glutinuous, glandular-punctate to glabrescent, glandular trichomes (when present) appearing sessile (i.e. on peduncles too short to be seen with a 40× magnification stereoscope). Abaxial surface usually green (when fresh), becoming pale green (when dry), always lighter than the adaxial surface, glandular-punctate to covered with eglandular or gland-tipped trichomes, or totally concealed by a lanose indumentum. Venation composed of 1–7 basal acrodromous veins, mid-vein stout, lateral veins becoming faint towards the leaf margin, impressed on the adaxial surface and prominent on the abaxial surface, consisting of amphicribral or bicollateral vascular bundles, tertiaries usually evident, nearly perpendicular to the mid-vein and branching towards the leaf margin. Inflorescences simple or compound dichasia, consisting of biparous cymes throughout or proximally biparous and distally uniparous cymes or reduced to solitary flowers on the apical region of the branches. Inflorescence bracts 0.7–5.0 cm long, 0.1–2.0 cm wide, petiolate, usually similar to the principal leaves in shape and indumentum, 1–5-nerved from the base. Bracteoles sessile or with petioles up to 6 mm long, blades 2.2–11 mm long, 0.5–5.5 mm wide, linear, elliptic, lanceolate, ovate, narrowly elliptic, oblong or oblanceolate, 1–3(–5)-nerved from the base, entire to slightly serrulate, rarely ciliate, usually differing in shape, but similar in indumentum to the principal leaves. Flowers (4–)5(–6)-merous, diplostemonous, pedicellate, subtended by a pair of bracteoles. Hypanthia 1.7–6.5 mm long, 1.5–5.2 mm wide at the torus, campanulate to urceolate, not fused to the ovary, externally glandular-punctate, sometimes covered with eglandular or gland-tipped trichomes, rarely completely concealed by a lanose indumentum, lacking a crown of trichomes at the apex. Calyx tubes 0.1–1.2 mm long, externally like the hypanthia. Calyx lobes 0.7–9.7 mm long, 0.4–3.2 mm wide at the base, oblong, triangular, narrowly triangular or subulate, entire or rarely sparsely ciliate, apex acute to acuminate, rarely terminating in an apical eglandular trichome, similar to the hypanthia in indumentum. Petals 4.5–26 mm long, 2.4–15 mm wide, obovate, entire, eventually ciliate, white, magenta or yellow (when fresh), apex acute, rounded, acuminate or emarginate, both surfaces glabrous or rarely sparsely glandular-punctate on the adaxial surface. Stamens (8)10(12), strongly dimorphic or subisomorphic, glabrous throughout, filaments linear, white, pink or yellow, pedoconnectives well-developed, anthers oblong, 2-celled (tetrasporangiate), rostrate, apical pores circular and ventrally inclined. Larger (antesepalous) stamens (4–)5(–6), filaments 1.5–6.3 mm long, pedoconnectives 1.3–7.3 mm long, ventral appendages 0.1–3.0 mm long, the apex usually emarginate to bilobate, thecae (excluding rostra) 0.8–3.8 mm long, purple, red, vinaceous or rarely yellow, rostra 0.2–0.7 mm long, pores 0.1–0.3 mm wide. Smaller (antepetalous) stamens (4–)5(–6), filaments 1.5–5.4 mm long, pedoconnectives 0.2–1.9 mm long, ventral appendages usually up to 0.1 mm long, apex truncate to emarginate, thecae (excluding rostra) 0.8–3.2 mm long, yellow to orange, rostra 0.2–0.6 mm long, pores ca. 0.2 mm wide. Ovaries 0.9–4.1 mm long, 0.7–3.1 mm wide, ovoid, cylindrical or globose, superior, (3–4–)5-locular, glabrous. Styles 3–10 mm long, filiform, sigmoid to incurved, white, pink or yellow, glabrous, stigmas punctiform. Capsules loculicidal, 2.3–8.0 mm long, 2.3–6.0 mm wide, ovoid or globose, the torus initially constricted at the apex, dehiscent from the apex to the base, columella deciduous. Fruiting calyx tubes 0.2–3.1 mm long. Fruiting calyx lobes 1.2–11.5 mm long, rarely thickened. Seeds 0.3–0.9 mm long, reniform, the testa foveolate-reticulate.

##### ﻿Nomenclatural notes

Based on collections housed in the herbarium at Paris (P), Martin and Cremers (2007) selected types for Melastomataceae described by Charles Victor Naudin (1815–1889), including six names for taxa that are part of the *Trembleya* s.s. clade (*Microliciacalycina*, *M.paniculata*, *M.pentagona*, *M.stenophylla*, *M.trembleyiformis* and *M.tridentata*). More than one specimen corresponding to the types cited by Martin and Cremers (2007) were found at P for the six names. These type citations were here treated as first-step lectotypifications. Thus, second-step lectotypes were designated in these cases following Article 9.17 of the Shenzhen Code (Turland et al. 2018). Overall, our nomenclatural update of this clade includes the designation of 34 lectotypes for names at specific and infraspecific ranks and 25 new synonyms.

### ﻿Key to the species of *Trembleya* s.s. clade of *Microlicia*

**Table:** 

1	Leaves 0.5–2 mm wide, 1-nerved from the base	**2**
–	Leaves 3–50 mm wide, 3–7-nerved from the base	**3**
2	Leaf blades linear; petals magenta; larger (antesepalous) stamens with anthers magenta to purple and smaller (antepetalous) stamens with yellow anthers	***M.pithyoides***
–	Leaf blades narrowly-lanceolate to narrowly-elliptic; petals yellow; all stamens with anthers yellow to pale brown	***M.rosmarinoides***
3	Plants with a dense whitish lanose indumentum covering branchlets, leaf abaxial surfaces and hypanthia; petals 19–26 mm long	***M.laniflora***
–	Plants lacking a whitish lanose indumentum; petals 4.5–13 mm long	**4**
4	Leaves with the abaxial surface entirely concealed by the indumentum, tertiary venation “foveolate-like” (Fig. 4M)	***M.chamissoana***
–	Leaves with the abaxial surface exposed, tertiary venation (if present) not “foveolate-like”	**5**
5	Leaves 3-nerved from the base (including a tenuous inframarginal pair of veins)	***M.calycina***
–	Leaves 5–7-nerved from the base (including a tenuous inframarginal pair of veins)	**6**
6	Abaxial leaf venation with tertiaries absent or little evident (Fig. 4B–E)	**7**
–	Abaxial leaf venation with tertiaries evident (Fig. 4A, H–M)	**8**
7	Calyx lobes subulate, 6.2–8.5 mm long in flower, becoming stout and thick in fruit (Fig. 12C–E); petals magenta; stamens strongly dimorphic (Minas Gerais)	***M.pentagona***
–	Calyx lobes oblong to triangular, 2.8–3.4 mm long in flower, tenuous in fruit; petals white; stamens subisomorphic (Chapada dos Veadeiros, Goiás)	***M.altoparaisensis***
8	Leaf margins entire along the basal half, sharply serrulate on the upper half (Fig. 4A), tertiary veins surrounding stout depressions on the abaxial leaf surface; bracteoles 8.1–11.0 mm long	***M.tridentata***
–	Leaf margins entire or slightly serrulate throughout, tertiary veins not surrounding stout depressions on the abaxial surface; bracteoles 2.2–6.1 mm long	**9**
9	Calyx lobes 4–4.9 mm long; petals yellow; all anthers yellow to pale brown	***M.flaviflora***
–	Calyx lobes 0.7–3 mm long; petals white, light pink or magenta; anthers of larger (antesepalous) stamens pink to magenta	**10**
10	Branchlets with narrow wings ca. 0.2 mm wide; flowers solitary; calyx lobes narrowly-triangular; ovaries 3–5-locular	***M.trembleyiformis***
–	Branchlets unwinged; flowers disposed in compound or simple dichasia; calyx lobes triangular; ovaries 5-locular	***M.parviflora***

### ﻿1. Microlicia altoparaisensis

Taxon classificationPlantaeMyrtalesMelastomataceae

(R.B.Pacifico, Almeda & Fidanza) Versiane & R.Romero, Bot. J. Linn. Soc. 197: 52. 2021.

F87374E3-E593-5DBF-9E74-297730868476

Fig. 18

Trembleya altoparaisensis R.B.Pacifico, Almeda & Fidanza, Phytotaxa 391(5): 291. 2019. **basionym**. Type: Brazil. Goiás, Alto Paraíso de Goiás, Parque Nacional Chapada dos Veadeiros, próximo da cachoeira do Rio Preto, perto do povoado de São Jorge, 6 February 1987, *J.R. Pirani 1663, R.M. Harley, B.L. Stannard, A. Furlan & C. Kameyama* (holotype: SPF!; isotypes: HUEM!, K!, UEC!, US!).

#### Description.

Erect shrubs 0.8–1.8 m tall. Branchlets quadrangular, glandular-punctate, light green to golden (when fresh). Internodes 1.0–2.1 cm long, angles unwinged. Petioles 2.0–5.5 mm long. Leaf blades 14–45 mm long, 3–9 mm wide, papyraceous (when dry), oblong to lanceolate, both surfaces green (when fresh), pale green (when dry), concolorous (when dry), base attenuate, apex rounded to acute, margin flat or slightly revolute, slightly serrulate and glandular-punctate, 5-nerved from the base, one pair of acrodromous veins and one pair of tenuous veins close to the margin, tertiaries little or not evident on the abaxial surface, adaxial surface densely glandular-punctate, abaxial surface densely glandular-punctate. Inflorescences compound dichasia consisting of proximally biparous, distally uniparous cymes, not congested. Bracts (including petioles) 1.0–4.0 cm long, 0.1–0.7 cm wide, 1-nerved, oblong to lanceolate, indumentum like that of the principal leaves. Bracteoles (at anthesis) sessile or with petioles up to 0.1–0.8 mm long, blades 5.0–6.7 mm long, 0.9–1.8 mm wide, oblong to oblanceolate, base acute, apex rounded to emarginate, margin entire and glandular-punctate, 1-nerved, indumentum like that of the principal leaves. Flowers (4–)5-merous, pedicels (at anthesis) inconspicuous up to 0.2 mm long. Hypanthia (at anthesis) 2.5–5.5 mm long, 3.1–3.2 mm wide at the torus, campanulate, light green to golden (when fresh), externally glandular-punctate. Calyx tubes 0.1–0.3 mm long. Calyx lobes (at anthesis) 2.8–3.4 mm long, 3.1–3.2 mm wide at the base, oblong to triangular, apex acute, eventually terminating in an eglandular trichome 0.1–0.4 mm long, margin entire, (when fresh) light green to golden, externally glandular-punctate. Petals 8.1–10.2 mm long, 3.2–4.5 mm wide, white, obovate, apex acuminate, margin entire and glabrous, both surfaces glabrous. Stamens (8)10, subisomorphic. Larger (antesepalous) stamens (4–)5, filaments 3.3–3.5 mm long, white, pedoconnectives 1.3–1.5 mm long, white, appendages ca. 0.1 mm long, white, apex emarginate, thecae (excluding rostra) 2.7–3.8 mm long, yellow, oblong, rostra 0.3–0.5 mm long, the circular pores ca. 0.2 mm wide. Smaller (antepetalous) stamens (4–)5, filaments 2.7–2.8 mm long, white, pedoconnectives 0.7–1.0 mm long, white, inconspicuous appendages ca. 0.1 mm long, white, apex truncate, thecae (excluding rostra) 2.5–3.2 mm long, yellow, oblong, rostra 0.3–0.5 mm long, the circular pores ca. 0.2 mm wide. Ovary 2.4–2.5 mm long, 1.4–1.6 mm wide, cylindrical, 3(–4)-locular. Style ca. 10 mm long, white. Capsules (at maturity) 4.5–5.3 mm long, 2.8–3.2 mm wide, ovoid, initially enveloped by the hypanthium, torus constricted at the apex, fruiting calyx tubes 0.4–0.5 mm long, fruiting calyx lobes 3.2–3.4 mm long, not thickened. Seeds not seen.

**Figure 18.**
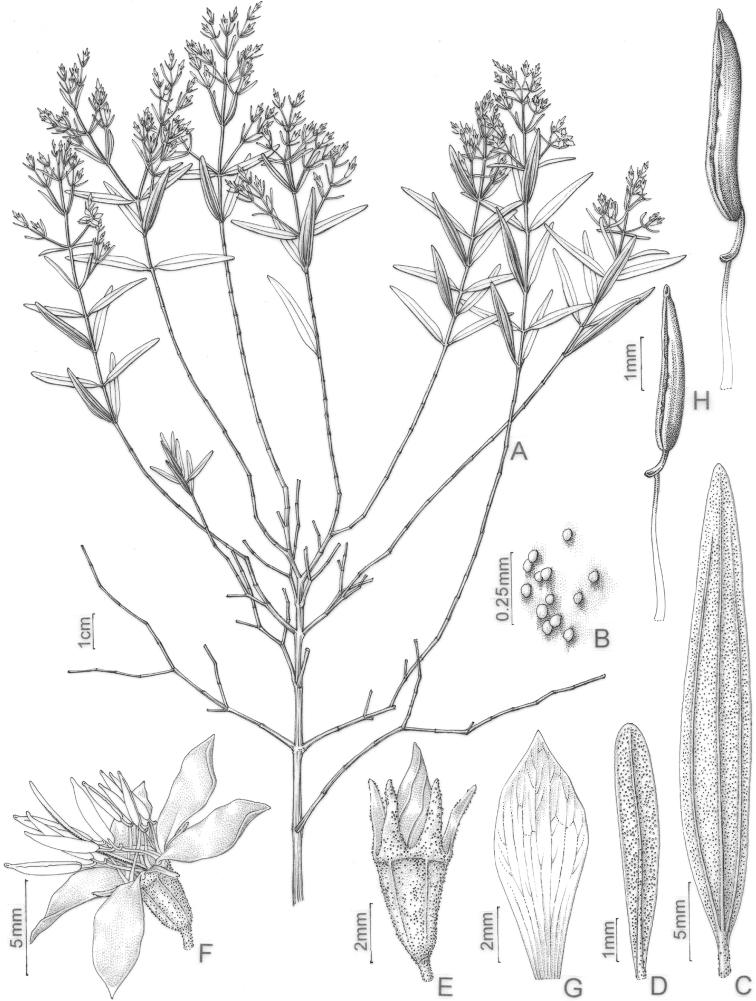
*Microliciaaltoparaisensis***A** habit **B** detail of the glandular-punctate indumentum on the branches **C** leaf abaxial surface **D** bracteole adaxial surface **E** floral bud **F** flower in lateral view **G** petal adaxial surface **H** antepetalous (left) and antesepalous (right) stamens. Drawn from Pirani et al. 1663 (UEC). Copyright Magnolia Press. Reproduced with permission from copyright holder.

#### Recognition and affinities.

*Microliciaaltoparaisensis* can be recognised by its oblong-lanceolate leaf blades (1.4–4.5 × 0.3–0.9 cm), papyraceous, 3–5-nerved from the base, glandular-punctate on both surfaces, tertiary veins not evident, inflorescences composed of proximally biparous and distally uniparous cymes, white petals, yellow subisomorphic stamens and 3(–4)-locular ovaries. The subisomorphic androecium is the most distinctive feature of *M.altoparaisensis* as it is unique in the clade. *Microliciaaltoparaisensis* is also the only species with amphistomatic leaves, attenuate thecae rostra and a distribution restricted to Goiás (Pacifico et al. 2019). Ovaries with 3–4 locules occur only in the apparently distantly related *M.trembleyiformis*, which differs in having leaves with tertiaries evident (vs. not evident), discoloured when dry (vs. concolorous), solitary flowers (vs. developed inflorescences), petals magenta (vs. white) and dimorphic stamens (vs. subisomorphic). Another possible relative is the widespread *M.parviflora* with a range that extends from Paraná to Bahia and Goiás and the only representative of the clade that grows with *M.altoparaisensis* at the Chapada dos Veadeiros. *Microliciaaltoparaisensis* differs from *M.parviflora* by the leaves with tertiaries not evident (vs. evident), concolorous when dry (vs. discoloured), amphistomatic (vs. hipostomatic), inflorescences with distally uniparous cymes (vs. biparous cymes), subisomorphic stamens (vs. dimorphic) and 5-locular ovaries (vs. 3–4-locular).

#### Distribution, habitat, and elevation range.

**Figure 19.**
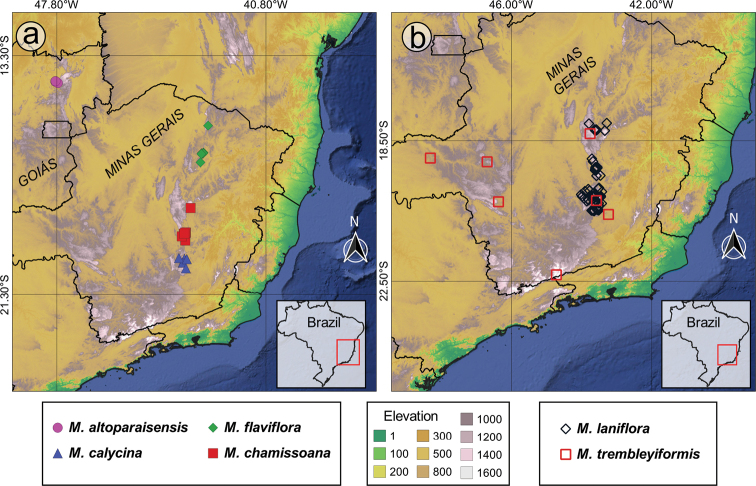
Geographic distribution of species of the *Trembleya* s.s. clade of *Microlicia***A** distributions of *M.altoparaisensis*, *M.calycina*, *M.chamissoana* and *M.flaviflora***B** distributions of *M.laniflora* and *M.trembleyiformis*.

Probably endemic to Chapada dos Veadeiros in Alto Paraíso de Goiás Municipality, Goiás, Brazil (Fig. 19A). It occurs in transitional formations between Cerrado and campo rupestre, on rocky quartzitic soils partially exposed to sun, at elevations of 1043–1086 m.

#### Conservation.

*Microliciaaltoparaisensis* is known from less than 10 collections. The EOO is 7.025 km^2^ and the AOO is 16 km^2^. Based on IUCN (2019) recommendations and criteria, we believe that this species should be classified as Endangered (EN): B1ab(iii). The majority of the populations of *M.altoparaisensis* occur inside Parque Nacional da Chapada dos Veadeiros, where this species is afforded some protection.

#### Notes.

The collection *Glaziou 21300* was listed under the name *Trembleyadebilis* by Glaziou (1908: 250). Following the proposal by Mansano and Pederneiras (2016), the catalogue of collections by Glaziou (1908) was included in the List of Suppressed Works for all taxonomic ranks in the Shenzhen Code (Turland et al. 2018). New names at specified ranks included in publications listed as suppressed works are not validly published according to Article 34 of the Code.

#### Specimens examined.

**Brazil. Goiás**: Alto Paraíso de Goiás Municipality, Chapada dos Veadeiros, Drummond et al. 321 (MBM, NY, RB), Klein et al. 2465 (HUFU, UFG), Machado et al. 153 (HUFU), Meyer 1171 (UEC, UPCB), Pacifico & Bressan 380 (CAS, HUEM, SPF), Pirani et al. 1663 (holotype: SPF; isotypes: HUEM, K, UEC, US), Pirani et al. 1694 (K, SPF, UEC); Unknown municipality, Fazenda da Boa-Vista, près Morro do Salto, Glaziou 21300 (F[photo], P, S).

### ﻿2. Microlicia calycina

Taxon classificationPlantaeMyrtalesMelastomataceae

(Cham.) Versiane & R.Romero, Bot. J. Linn. Soc. 197: 52. 2021.

70DE2883-7B9C-588E-8B8C-BFF777DBA5F0

Fig. 20

Trembleya calycina Cham., Linnaea 9(4): 430. 1835. **basionym**. Type: Brazil. “Brasilia, Itacolumi” [Minas Gerais, Ouro Preto], *F. Sellow s.n.* (lectotype, designated here: K [K00530658]!; isolectotypes: BR [BR0000005227020]!, F [neg. 16634]!, K [K00530659]!; image of lectotype is available at http://specimens.kew.org/herbarium/K000530659).
Trembleya revoluta Naudin, Ann. Sci. Nat., Bot. Sér. 3, 2: 155. 1844. Type: Brazil. “Minas Gerais, Capanema” [Minas Gerais, Santa Bárbara], 1841, *P. Claussen 10* (lectotype, first-step designated by Martin and Cremers (2007), second-step designated here: P [P00723384]!; isolectotypes: G [G00368001]!, P [P00723507]!; image of lectotype is available at http://coldb.mnhn.fr/catalognumber/mnhn/p/p00723384).
Trembleya stenophylla Naudin, Ann. Sci. Nat., Bot. Sér. 3, 12: 265. 1849. **syn. nov.** Type: Brazil. “Minas Gerais, Capanema” [Minas Gerais, Santa Bárbara], 1843, *P. Claussen 368* (lectotype, first-step designated by Martin and Cremers (2007), second-step designated here: P [P00723385]!; isolectotype: P [P00723386]!; image of lectotype is available at http://coldb.mnhn.fr/catalognumber/mnhn/p/p00723385).

#### Description.

Erect shrubs 0.65–1.5 m tall. Branchlets quadrangular, glandular-punctate and sparsely covered with glandular trichomes 0.1–0.2 mm long, light green (when fresh). Internodes 1.5–3.0 cm long, angles with narrow wings 0.2–0.4 mm wide. Petioles 0.8–1.8 mm long. Leaf blades 10–26 mm long, 2–9 mm wide, chartaceous (when dry), elliptic to narrowly elliptic, both surfaces green (when fresh), adaxial surface blackened and abaxial surface pale green (when dry), discoloured (when dry), base attenuate, apex rounded to acute, margin revolute, entire along the basal half, appearing entire to slightly serrulate on the upper half and minutely granulose and becoming glabrescent with age, 3-nerved from the base, one tenuous pair of acrodromal veins, tertiaries evident on the abaxial surface, nearly perpendicular to the mid-vein, little reticulate and branching apically, foveolate-like, adaxial surface sparsely glandular-punctate, appearing glabrous when dry, abaxial surface densely glandular-punctate. Inflorescences simple dichasia or reduced to solitary flowers, not congested. Bracts (including petioles) 1.0–1.3 cm long, 0.3–0.4 cm wide, 3-nerved, elliptic to narrowly elliptic, indumentum like that of the principal leaves. Bracteoles (at anthesis) sessile or with petioles up to 1.0 mm long, blades 4.0–5.0 mm long, 1.3–1.8 mm wide, elliptic, base attenuate, apex rounded to acute, margin entire and glandular-punctate, 1-nerved, indumentum like that of the principal leaves. Flowers 5-merous, pedicels (at anthesis) 0.5–0.7 mm long. Hypanthia (at anthesis) 2.6–3.5 mm long, 1.9–2.2 mm wide at the torus, campanulate, light green or reddish (when fresh), externally glandular-punctate. Calyx tubes 0.4–0.7 mm long. Calyx lobes (at anthesis) 3.5–5.2 mm long, 0.5–0.7 mm wide at the base, subulate, apex acuminate, margin entire, (when fresh) light green or reddish, externally glandular-punctate. Petals 7.8–10 mm long, 5.0–6.2 mm wide, magenta, obovate, apex acuminate, margin entire and glabrous, both surfaces glabrous. Stamens 10, strongly dimorphic. Larger (antesepalous) stamens 5, filaments 4.7–4.9 mm long, pink, pedoconnectives 5.9–6.2 mm long, pink, appendages 1.4–1.6 mm long, yellow, apex truncate to slightly emarginate, thecae (excluding rostra) 2.3–2.6 mm long, purple, oblong, rostra 0.4–0.7 mm long, the circular pores ca. 0.2 mm wide. Smaller (antepetalous) stamens 5, filaments 3.8–4.1 mm long, pink, pedoconnectives 0.6–0.8 mm long, yellow, inconspicuous appendages ca. 0.1 mm long, yellow, apex truncate, thecae (excluding rostra) 2.1–2.3 mm long, yellow, oblong, rostra 0.4–0.6 mm long, the circular pores ca. 0.2 mm wide. Ovary 2.0–2.2 mm long, 1.9–2.1 mm wide, globose, 5-locular. Style ca. 6.5 mm long, pink. Capsules (at maturity) 2.3–2.7 mm long, 2.3–2.7 mm wide, globose, initially enveloped by the hypanthium, torus constricted at the apex, fruiting calyx tubes 0.7–0.8 mm long, fruiting calyx lobes 5.5–6.0 mm long, not thickened. Seeds ca. 0.8 mm long, reniform.

**Figure 20.**
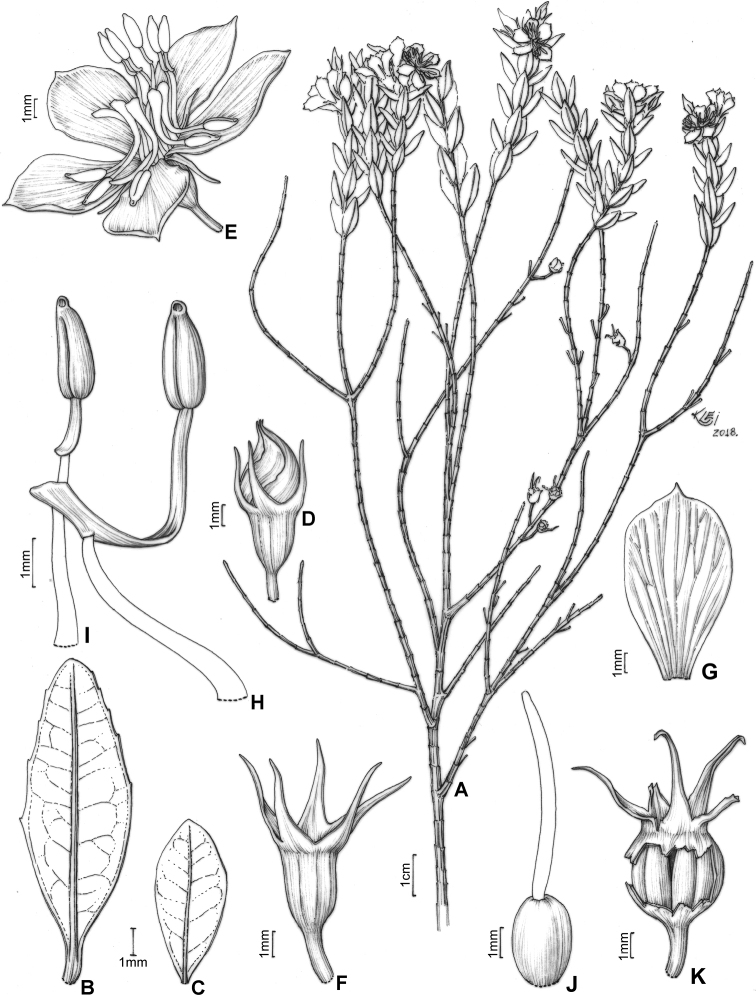
*Microliciacalycina***A** habit **B** leaf abaxial surface **C** bracteole abaxial surface **D** floral bud **E** flower in lateral view **F** flowering hypanthium **G** petal adaxial surface **H** antesepalous stamen **I** antepetalous stamen **J** gynoecium **K** capsule. Drawn from Barreto 9019 (UEC).

#### Recognition and affinities.

*Microliciacalycina* may be recognised by its elliptic to narrowly elliptic leaf blades with revolute margins, 3-nerved from the base, simple dichasia or solitary flowers and subulate calyx lobes 3.5–5.2 mm long. In morphology, *M.calycina* resembles narrow-leaved forms of *M.pentagona* (see notes under this species). Both species share the glandular-punctate indumentum, inflorescences reduced to solitary flowers (sometimes perfect dichasia only in *M.calycina*), subulate calyx lobes, magenta petals, and bicoloured anthers. *Microliciacalycina* differs by the leaf blades that are 3-nerved from the base (vs. 5-nerved) and calyx lobes 3.5–5.2 mm long (vs. 6.2–8.5 mm long) that become thick in fruit (vs. tenuous). These two species may occur sympatrically in the seasonally dry grasslands of Parque Estadual do Itacolomi (Rolim 2011) and at Serra do Caraça. *Microliciaparviflora* is also morphologically similar to *M.calycina*, which may be distinguished by leaves 3-nerved from the base (vs. 5-nerved) and subulate calyx lobes (vs. triangular). Additionally, *M.parviflora* is distinct in having openly ramified inflorescences that are sometimes reduced to simple dichasia. In turn, the inflorescences of *M.calycina* consist of simple dichasia that are frequently reduced to solitary flowers. The distributions of *Microliciacalycina* and *M.parviflora* overlap in Caeté, Ouro Preto and Catas Altas, where sympatry is likely to occur. *Microliciacalycina* is also similar to *M.pithyoides* (see notes under this species).

#### Distribution, habitat and elevation range.

Endemic to central and southern Minas Gerais (Fig. 19A), at Serra do Caraça, Serra do Itacolomi and Serra da Piedade. It occurs on quartzitic campo rupestre exposed to full sun at elevations between 1692 and 1920 m.

#### Conservation.

*Microliciacalycina* is known from about 20 collections. The EOO is 481 km^2^ and the AOO is 20 km^2^. This species is currently recognised as endangered (EN) by the Brazilian Government (Brasília 2014). Based on IUCN (2019) recommendations and criteria, we recommend a similar assessment: (EN): B1ab(iii). The conservation units of Parque Estadual do Itacolomi and RPPN Serra do Caraça (Natural Heritage Private Reserve) are of major importance for the long-term conservation of *M.calycina*.

#### Notes.

We agree with Cogniaux (1883–1888) and treat *Trembleyarevoluta* as a synonym of *M.calycina*. The type of *Trembleyarevoluta* (*P. Claussen 10*) differs from typical *M.calycina* collections only by its revolute leaf margins, a feature that appears to be an artefact of drying. Likewise, we consider *Trembleyastenophylla* a narrow-leaved form of *M.calycina*. Specimens of *M.calycina* with leaf blades conspicuously revolute have also been confused with *M.pithyoides*. For a comparison of *M.calycina* and *M.pithyoides*, see the notes under the latter.

#### Specimens examined.

**Brazil. Minas Gerais**: Caeté Municipality, Serra da Piedade, Grandi et al. 6593 (BHCB, HUFU); Catas Altas Municipality, Serra do Caraça, Castro et al. 283 (HUFU), Oliveira & Giacomin 47 (BHCB), Oliveira & Giacomin 84 (BHCB), Oliveira et al. 480 (BHCB), Pacifico & Bressan 296 (CAS, HUEM, SPF); Ouro Preto Municipality, Serra do Itacolomi, Barreto 9019 (BHCB, ESA, FUEL, HUFU, SP, SPF, UEC, UPCB), Damazio s.n. (RB [48391]), Glaziou 14745 (P), Glaziou 18232 (K, P, R), Pacifico & Bressan 291 (CAS, HUEM, SPF), Peron 220 (RB), Peron 268 (RB), Peron 269 (RB), Riedel s.n. (K [K00530657], NY [NY00941982], W [18890019737]), Rolim 366 (HUFU, NY, RB, VIC), Schwacke 9368 (RB, W); Unknown municipality, Claussen 10 (P [P00723384, P00723507]), “capanema”, Claussen 368 (P [P00723385, P00723386]), Sellow s.n. (lectotype: K [K00530659]; isolectotypes: BR [BR0000005227020], F [neg. 16634], K [K00530658]).

### ﻿3. Microlicia chamissoana

Taxon classificationPlantaeMyrtalesMelastomataceae

(Naudin) Versiane & R.Romero, Bot. J. Linn. Soc. 197: 53. 2021.

70EFD5E7-308A-5BA5-B539-DBD9FC1EC173

Fig. 21

Trembleya chamissoana Naudin, Ann. Sci. Nat., Bot. Sér. 3, 12: 270. 1849. **basionym**. Type: Brazil. “Brasilia, Itambé” [Minas Gerais, probably Santo Antônio do Itambé], *F. Sellow s.n.* [b. 1171 c. 1156] (lectotype, designated here: K [K00530656]!; isolectotype: P [P00723508]!; image of lectotype is available at http://specimens.kew.org/herbarium/K000530656).

#### Description.

Erect shrubs (0.1–)0.3–1.5 m tall. Branchlets quadrangular, glandular-punctate and covered with gland-tipped trichomes 0.2–0.4 mm long, light green (when fresh). Internodes 0.3–1.5 cm long, angles unwinged. Petioles 1.0–4.9 mm long. Leaf blades 10–28 mm long, 4–18 mm wide, chartaceous (when dry), elliptical, both surfaces green (when fresh), adaxial surface blackened and abaxial surface pale green (when dry), discoloured (when dry), base attenuate, apex obtuse to acute, margin flat or slightly revolute, entire throughout or slightly serrulate on the upper half and glandular-punctate, 7-nerved from the base, two pairs of acrodromous veins and one tenuous pair of veins close to the margin, tertiaries evident on the abaxial surface, nearly perpendicular to the mid-vein, reticulate and randomly branching, adaxial surface sparsely glandular-punctate, appearing glabrous when dry, abaxial surface densely glandular-punctate and covered with gland-tipped trichomes 0.2–0.4 mm long. Inflorescences simple or compound congested dichasia consisting of biparous cymes, or reduced to solitary flowers. Bracts (including petioles) 0.8–1.0 cm long, 0.6–0.7 cm wide, 5-nerved, elliptical, indumentum like that of the principal leaves. Bracteoles (at anthesis) with petioles 1.6–2.0 mm long, blades 4.2–4.9 mm long, 1.4–2.1 mm wide, narrowly elliptic, base attenuate, apex acuminate, margin entire along the basal half, sparsely serrulate on the upper half, 3–5-nerved, indumentum like that of the principal leaves. Flowers 5-merous, pedicels (at anthesis) 2–4 mm long. Hypanthia (at anthesis) 2.5–3.7 mm long, 1.9–2.1 mm wide at the torus, campanulate, reddish (when fresh), externally glandular-punctate and sparsely to densely covered with gland-tipped trichomes 0.2–0.4 mm long. Calyx tubes 0.9–1.2 mm long. Calyx lobes (at anthesis) 4.5–6.7 mm long, 1.9–2.1 mm wide at the base, narrowly triangular, apex acuminate, margin entire and sparsely ciliate with gland-tipped trichomes 0.2–0.4 mm long, (when fresh) reddish, externally glandular-punctate and covered with gland-tipped trichomes 0.2–0.4 mm long. Petals 11.5–13 mm long, 5.8–6.2 mm wide, magenta, obovate, apex acuminate, margin entire and glandular-punctate, adaxial surface sparsely glandular-punctate, abaxial surface glabrous. Stamens 10, strongly dimorphic. Larger (antesepalous) stamens 5, filaments 5.2–6.0 mm long, pink, pedoconnectives 5.5–6.5 mm long, pink, appendages 1.2–1.8 mm long, yellow, apex bilobate, thecae (excluding rostra) 1.7–2.6 mm long, purple, oblong, rostra 0.3–0.6 mm long, the circular pores ca. 0.3 mm wide. Smaller (antepetalous) stamens 5, filaments 4.7–5.0 mm long, pink, pedoconnectives 1.0–1.2 mm long, yellow, inconspicuous appendages ca. 0.1 mm long, yellow, apex truncate, thecae (excluding rostra) 1.5–2.0 mm long, yellow, oblong, rostra 0.4–0.6 mm long, the circular pores ca. 0.2 mm wide. Ovary 2.2–2.8 mm long, 1.6–1.8 mm wide, cylindrical, 5-locular. Style ca. 6.3 mm long, pink. Capsules (at maturity) 2.5–3.2 mm long, 2.3–3.0 mm wide, ovoid, initially enveloped by the hypanthium, torus constricted at the apex, fruiting calyx tubes 1.5–1.7 mm long, fruiting calyx lobes 5.2–6 mm long, not thickened. Seeds ca. 0.5 mm long, reniform.

**Figure 21.**
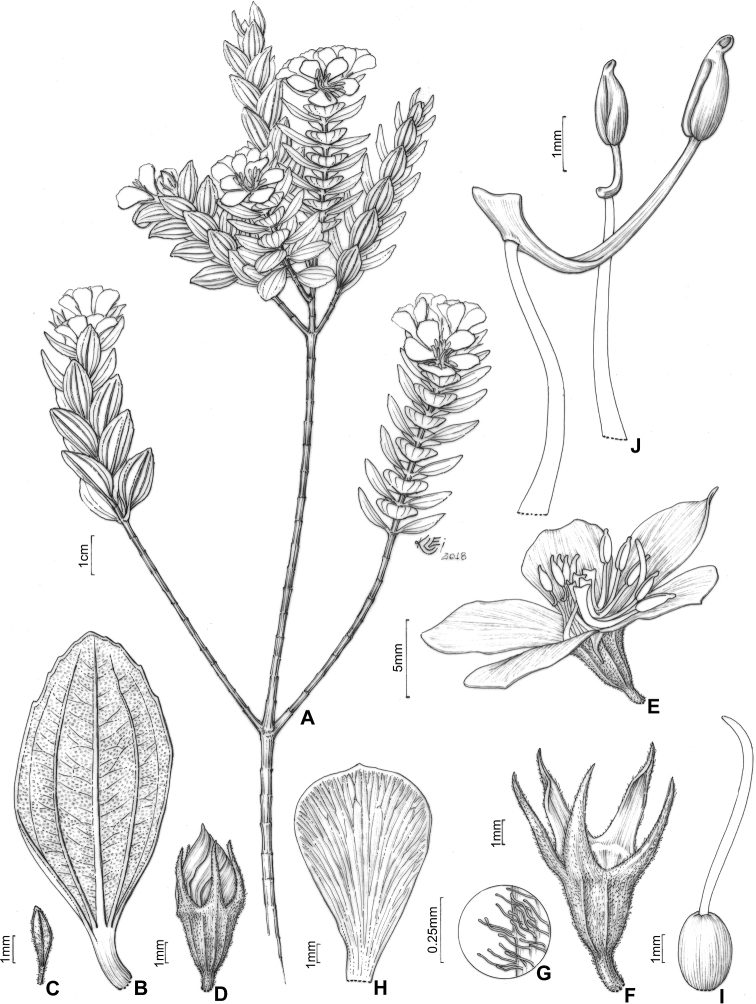
*Microliciachamissoana***A** habit **B** leaf abaxial surface **C** bracteole abaxial surface **D** floral bud **E** Flower in lateral view **F** flowering hypanthium **G** detail of the indumentum of the hypanthium **H** petal adaxial surface **I** gynoecium **J** antepetalous (behind) and antesepalous (in front) stamens. Drawn from Barreto 6745 (SPF, UEC).

#### Distribution, habitat and elevation range.

Largely restricted to the Serra do Cipó in central Minas Gerais (Fig. 19A), but extending to the Serra dos Alves and probably to Pico do Itambé. Most of the recent collections were made at Serra do Cipó; only one collection studied came from Serra dos Alves, *Souza & Miranda 1639* (BHCB). Considering Sellow’s itinerary in Brazil (see Rego et al. 2013), the type series was probably collected at the Pico do Itambé region. It occurs on quartzitic campo rupestre exposed to full sun at elevations between 1154 and 1462 m.

#### Conservation.

*Microliciachamissoana* is known from fewer than 20 collections. The EOO is 885.927 km^2^ and the AOO is 32 km^2^. This species was collected on Pico do Itambé only in the 19^th^ century and the local extinction of that population is a possibility. Several populations found more recently are protected in the Parque Nacional da Serra do Cipó. This species is already recognised as endangered (EN) by the Brazilian Government (Brasília 2014). Our study based on IUCN (2019) recommendations and criteria reached a similar conclusion regarding its conservation status (EN): B1ab(iii).

#### Recognition and affinities.

*Microliciachamissoana* may be recognised by its elliptic leaf blades with tertiaries densely reticulate and randomly branching, congested inflorescences or solitary flowers and narrowly triangular calyx lobes 4.5–5.7 mm long. It is probably more closely related to *M.laniflora* and *M.pentagona*, both of which may occur sympatrically with *M.chamissoana* at Serra do Cipó. *Microliciachamissoana* differs from *M.laniflora* by the shorter height (0.1–)0.5–0.8 m tall (vs. 0.5–3.5 m tall), absence of lanose indumentum on branchlets, abaxial leaf surfaces and hypanthia (vs. present), leaves with shorter petioles 1.0–4.9 mm long (vs. 6–11 mm long), blades with tertaries densely reticulate and randomly branching (vs. little reticulate and branching apically), bracteoles with apices acuminate (vs. rounded), shorter hypanthia 2.5–3.7 mm long (vs. 5.0–6.5 mm long), shorter calyx lobes 4.5–6.7 mm long (vs. 7.9–9.7 mm long) and petals magenta (vs. white) 11.5–13.0 mm long (vs. 19.0–26.0). In turn, *Microliciachamissoana* differs from *M.pentagona* by the branchlets, abaxial surfaces of the leaves and hypanthia that are densely glandular-punctate and covered with gland-tipped trichomes (vs. appearing glabrous, vernicose and minutely granulose), leaf blades with tertiaries densely reticulate and randomly branching (vs. parallel or little reticulate and branching apically) and calyx lobes tenuous (vs. thickened) 5.2–6.0 mm long (vs. 6.5–11.0 mm long).

#### Notes.

Major variation in *M.chamissoana* involves habit and degree of inflorescence development. This species is usually a shrub about 1 m tall, although an atypical specimen from Serra do Cipó is about 10 cm tall (*A.M. Giulietti et al. CFSC12492*). This specimen was described as a herb, but it has woody branches. Most of the specimens examined have congested, many-flowered inflorescences (e.g. *Barreto 6745*), although in some of the inflorescences, these are reduced to solitary flowers (e.g. *Pacifico & Carmo 154*, *Almeda et al. 8580*).

Based on *F. Sellow s.n.*, Chamisso (1834: 396–397) provided a detailed description for *M.chamissoana* under *Microlicia* sp., indicating his uncertainty of its generic position, especially because of its 5-valvate capsules. Naudin (1849: 270) proposed the epithet *chamissoana* for this species mentioning the description of Chamisso (1834: 396–397). Even preceded by a short description that is not diagnostic (5-valvate capsules), the name *Trembleyachamissoana* Naudin is still valid because Naudin (1849) made reference to Chamisso’s decription.

The type specimens of *M.chamissoana*, as cited by Cogniaux (1883–1888), have two collection numbers indicated on each of their labels (*F. Sellow 1171* and *1156*). Both collection numbers were cited by Cogniaux in “Flora brasiliensis”. At least one set of Sellow’s duplicates of *M.chamissoana* was housed at B and probably destroyed during World War II. As the *Sellow* duplicate at K is in good shape, it is here designated as the lectotype for this species.

#### Specimens examined.

**Brazil. Minas Gerais**: “Itambé” [probably Santo Antônio do Itambé Municipality], Sellow s.n. (lectotype: K [K00530656]; isolectotype: P [P00723508]); Conceição do Mato Dentro Municipality, Serra do Cipó, Kameyama et al. CFSC10403 (SPF), Sheperd & Kirzenzaft 10214 (SP); Itabira Municipality, Serra dos Alves, Souza & Miranda 1639 (BHCB); Jaboticatubas Municipality, Serra do Cipó, Giulietti et al. CFSC12560 (SPF); Morro do Pilar Municipality, Serra do Cipó, Silveira s.n. (HUFU [56533]); Santana do Riacho Municipality, Serra do Cipó, Almeda et al. 8580 (CAS, HUEM, UEC), Escaramai et al. 52 (SPF), Giulietti et al. CFSC12492 (HUEM, SPF), Pacifico & Carmo 154 (HUEM), Pena & Viana 417 (SPF), Rocha 694 (BHCB), Romero et al. 8627 (HUEM, HUFU, RB), Semir CFSC5607 (SP); Unknown municipality, Serra do Cipó, Barreto 6745 (BHCB, HUFU, NY, SP, SPF, UEC, UPCB), Damazio 2026 (RB), Sena s.n. (W [W19110004181]).

### ﻿4. Microlicia flaviflora

Taxon classificationPlantaeMyrtalesMelastomataceae

Versiane & R.Romero, Bot. J. Linn. Soc. 197: 53. 2021.

1E659219-B757-51EC-AD15-FFAF85F0EC03

Fig. 22

Trembleya hatschbachii Wurdack & E.Martins, Bol. Bot. Univ. São Paulo 14: 40. 1995. **original name**. Type: Brazil. Minas Gerais, Grão Mogol, Rio das Mortes, 15 May 1988, *G. Hatschbach, M. Hatschbach & O. Ribas 52005* (holotype: MBM!; isotypes: BHCB!, C!, CAS!, CTES, ESA!, G!, HUFSJ, K!, MO!, RB!, S, SPF!, US!, VIC, VIES).

#### Description.

Erect shrubs 0.8–2.5 m tall. Branchlets quadrangular, appearing glabrous, vernicose and minutely granulose, light green (when fresh). Internodes 0.6–3.0 cm long, angles with narrow wings 0.2–0.4 mm wide. Petioles 3.9–17 mm long. Leaf blades 37–90 mm long, 17–50 mm wide, coriaceous (when dry), elliptic to slightly ovate, both surfaces green (when fresh), adaxial surface blackened and abaxial surface pale brown (when dry), discoloured (when dry), base cuneate to attenuate, apex acute, margin flat, entire and minutely granulose and becoming glabrescent with age, 5-nerved from the base, one pair of acrodromous veins and one pair of tenuous veins close to the margin, tertiaries evident on the abaxial surface, nearly perpendicular to acute to the mid-vein, little reticulate and branching apically, adaxial surface glabrous to minutely granulose, vernicose, abaxial surface glabrous to minutely granulose. Inflorescences compound dichasia consisting of biparous cymes, not congested. Bracts (including petioles) 3.2–5.0 cm long, 1.4–2.0 cm wide, 5-nerved, elliptical, appearing glabrous, vernicose. Bracteoles (at anthesis) with petioles 1.6–1.9 mm long, blades 3.5–6.0 mm long, 1.3–1.9 mm wide, narrowly elliptic, base attenuate, apex acute, margin entire, 1–3-nerved, indumentum appearing glabrous, vernicose. Flowers 5-merous, pedicels (at anthesis) 1.8–2.2 mm long. Hypanthia (at anthesis) 3.3–4.1 mm long, 3.0–3.2 mm wide at the torus, campanulate, light green (when fresh), externally glabrous, minutely granulose, vernicose. Calyx tubes inconspicuous, 0.1–0.2 mm long. Calyx lobes (at anthesis) 4.0–4.9 mm long, 1.3–1.9 mm wide at the base, narrowly triangular, apex acute, margin entire, (when fresh) light green, externally glabrous, minutely granulose, vernicose. Petals 6.0–8.8 mm long, 5.2–7 mm wide, yellow, obovate, apex rounded, margin entire and glabrous, both surfaces glabrous. Stamens 10, strongly dimorphic. Larger (antesepalous) stamens 5, filaments 3.4–4.0 mm long, yellow, pedoconnectives 3.7–4.0 mm long, yellow, appendages 1.0–1.2 mm long, yellow, apex truncate to slightly emarginate, thecae (excluding rostra) 1.4–1.6 mm long, brownish, oblong, rostra 0.3–0.4 mm long, the circular pores ca. 0.2 mm wide. Smaller (antepetalous) stamens 5, filaments 2.7–2.9 mm long, yellow, pedoconnectives 1.2–1.4 mm long, yellow, short appendages ca. 0.5 mm long, yellow, apex truncate, thecae (excluding rostra) 1.4–1.6 mm long, yellow-brownish, oblong, rostra 0.3–0.5 mm long, the circular pores ca. 0.2 mm wide. Ovary 3.5–4.1 mm long, 2.9–3.1 mm wide, globose, 5-locular. Style 4–4.2 mm long, yellow. Capsules (at maturity) 3.4–3.6 mm long, 3.5–4.2 mm wide, globose, initially enveloped by the hypanthium, torus constricted at the apex, fruiting calyx tubes 0.2–0.4 mm long, fruiting calyx lobes 3.7–4.0 mm long, not thickened. Seeds ca. 0.6 mm long, reniform.

**Figure 22.**
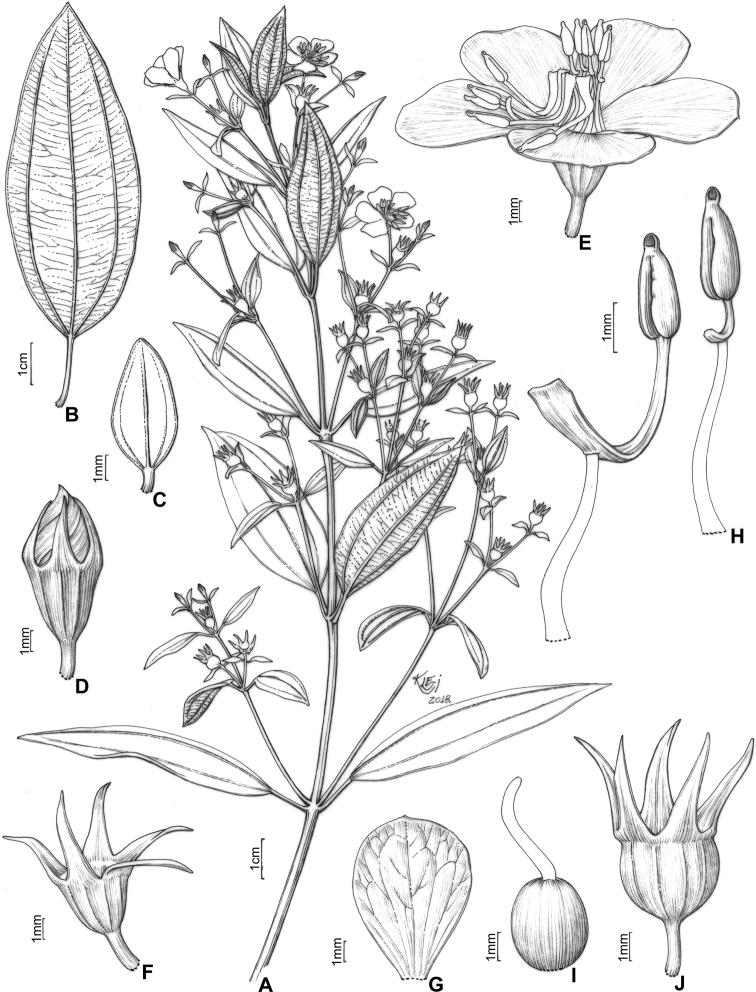
*Microliciaflaviflora***A** habit **B** leaf abaxial surface **C** bracteole abaxial surface **D** floral bud **E** flower in lateral view **F** flowering hypanthium **G** petal adaxial surface **H** antesepalous (left) and antepetalous (right) stamens **I** gynoecium **J** capsule enveloped by the hypathium. Drawn from Meireles et al. 1124 (UEC).

#### Distribution, habitat and elevation range.

Endemic to northern Minas Gerais (Fig. 19A), at Serra de Grão Mogol, Serra de Botumirim and Serra Nova. It occurs on quartzitic campo rupestre exposed to full sun at elevations between 760 and 1243 m. The distribution of *M.flaviflora* is a good match to the Grão Mogol biogeographic district (Colli-Silva et al. 2019).

#### Conservation.

This species is known from about 20 collections. The EOO is 468,668 km^2^ and the AOO is 32 km^2^. Most of the populations of *M.flaviflora* occur within the following conservation units: Parque Estadual de Grão Mogol, Parque Estadual de Botumirim and Parque Estadual da Serra Nova, where this species is afforded protection. The Brazilian Government assigned a conservation status of Endangered (EN) to this species (Brasília 2014). Based on IUCN (2019) recommendations and criteria, we concur with that conclusion (EN): B1ab(iii).

#### Recognition and affinities.

*Microliciaflaviflora* may be recognised by its leaves and hypanthia that appear to be glabrous, but are vernicose and minutely granulose, leaf blades 3.7–9.0 cm long, elliptic to slightly ovate, compound dichasia and yellow petals. In overall vegetative morphology, *M.flaviflora* resembles *M.tridentata*. In turn, its yellow petals, staminal filaments and styles are shared only with *M.rosmarinoides*. *Microliciaflaviflora* differs from *M.tridentata* by its leaves that have entire margins throughout (vs. serrulate along the upper half), abaxial surfaces appearing glabrous (vs. glandular-punctate), shorter bracteoles with blades 3.5–6.0 mm long (vs. 8.1–11.0 mm long) and apex acute (vs. rounded to obtuse) and yellow petals (vs. magenta or rarely white) that are 6.0–8.8 mm long (vs. 11.5–13.0 mm long). *Microliciaflaviflora* differs from *M.rosmarinoides* by its taller habit 0.8–2.0 m tall (vs. 0.3–0.6 m tall), branchlets, abaxial surfaces of the leaves and hypanthia appearing glabrous (vs. glandular-punctate), leaf blades 3.7–9.0 cm long (vs. 0.4–1.0 cm long) that are elliptic to slightly ovate (vs. linear to lanceolate) and have 5 basal acrodromous veins (vs. 1-nerved from the base), compound dichasia (vs. solitary flowers), longer calyx lobes 4.0–4.9 mm long (vs. 2.2–2.8 mm long) and longer petals 6.0–8.8 mm long (vs. 5.0–5.3 mm long).

#### Specimens examined.

**Brazil. Minas Gerais**: Botumirim Municipality, Estrada para o Rio do Peixe, Forzza et al. 4897 (NY, RB, SPF), Serra da Canastra, Mello-Silva et al. 509 (HUEM, SPF, UEC), Nakajima et al. 4764 (HUFU), Scatigna & Galvão 376 (UEC); Grão Mogol Municipality, Serra de Grão Mogol, Bidá et al. CFCR11951 (SPF, US), Cerati et al. 246 (K, SP, UEC), Furlan et al. CFCR771 (SPF, UEC, US), Hatschbach & Hatschbach 52005 (holotype: MBM; isotypes: BHCB, C, CAS, CTES, ESA, G, HUFSJ, K, MBM, MO, S, SPF, US, UPCB, VIC, VIES), Hatschbach 41337 (ESA, FLOR, HCF, HUEFS, MBM, NY, RB, SPF, UPCB, US), Hatschbach et al. 54239 (CAS, INPA, MBM), Hatschbach et al. 68067 (MBM), Hensold et al. CFCR3525 (SPF, US), Kral et al. 72723 (SP, SPF), Leitão Filho et al. 7893 (MBM, UEC), Meireles et al. 1124 (CAS, HUEM, UEC), Oliveira et al. CFCR12997 (SPF, US), Pacifico & Simoes 353 (CAS, HUEM), Pacifico 565 (CAS, HUEM, RB); Pirani & Mello-Silva CFCR10814 (HUEM, SPF, UEC, US), Zappi et al. CFCR9903 (SPF, UEC); Rio Pardo de Minas Municipality, Serra Nova, Araújo et al. 2043 (BHCB), Rocha et al. 497 (BHCB, NY).

### ﻿5. Microlicia laniflora

Taxon classificationPlantaeMyrtalesMelastomataceae

(D.Don) Baill., Adansonia 12: 95. 1877.

8BAA1DD6-BFC6-5991-90E5-FE9B2BD70381

Fig. 23

Melastoma laniflora D.Don, Mem. Wern. Nat. Hist. Soc. 4: 292. 1823. **basionym**. Type: Brazil. “in Brazilia”, *F. Sellow s.n.* (lectotype, designated here: K [K00957812]!; probable isolectotypes: K [K00957818]!, P [P005317063]!, P [P005317064]!, P [P005317070]!; image of lectotype is available at http://specimens.kew.org/herbarium/K000957812).
Trembleya lychnitis Schrank & Mart. ex DC., Prod. 3: 126. 1828. Type: Brazil. “In Brasiliae lapidosis apricis ad latera montium prov. Min. gener.” [Minas Gerais], *C.F.P. Martius s.n.* (lectotype, designated here: G [G00368004]!; isolectotype: M [M0165875]!).
Trembleya laniflora (D.Don) Cogn. in Martius et al., Fl. Bras. 14(3): 130. 1883.Type: Based on Melastomalaniflora D.Don.
Trembleya laniflora var. acutifolia Cogn. in Martius et al., Fl. Bras. 14(3): 131. 1883. **syn. nov.** Type: Brazil. “Brasilia meridionalis, ad Serra da S. Antonio” [Minas Gerais], 1878, *F. Sellow 1727* (lectotype, designated here: K [K00530655]!; isolectotype: P [P00723419]!; image of lectotype available at http://specimens.kew.org/herbarium/K000530655).
Trembleya laniflora var. genuina Cogn. in Martius et al., Fl. Bras. 14(3): 130. 1883. **syn. nov.** Type: Brazil. “Minas Geraes” [Minas Gerais], 1840, *P. Claussen 332A* (lectotype, designated here: BR [BR0000005228225]!, isolectotype: BR [BR0000005227891]!).
Trembleya laniflora var. grandifolia Cogn. in Martius et al. Fl. Bras. 14(3):131. 1883. **syn. nov.** Type: Brazil. “ad Pico d’Itabira et ad Caxoeira do Campo” [Minas Gerais, Itabira and Cachoeira do Campo], *C.F.P. Martius 930* (lectotype, designated here: P [P005317085]!; isolectotypes: BM [BM00516949]!, G [G00368005]!, G [G00318589]!, GH [GH00053137]!, L [L00056323]!, L [L00056324]!, M [M0165876]!, M [M0165877]!, M [M0165878]!, P [P005317086]!, S [S09-12961]!; image of lectotype is available at http://coldb.mnhn.fr/catalognumber/mnhn/p/p05317085).
Trembleya laniflora var. intermedia Cogn. in Martius et al., Fl. Bras. 14(3):130. 1883. **syn. nov.** Type: Brazil. “In prov. Minas Geraës loco haud indicato” [Minas Gerais], *G. Gardner 4601* (lectotype, designated here: BM [BM00525899]!; isolectotypes: G [G00318586]!, GH [GH00053136]!, NY [NY00245856]!, P [P005317081]!, R [R000168426]! US [US00623967]!; image of isolectotype at P is available at http://coldb.mnhn.fr/catalognumber/mnhn/p/p05317081).

#### Description.

Erect shrubs or treelets 0.5–3.5 m tall. Branchlet surfaces concealed by a lanose indumentum of eglandular trichomes 0.1–0.5 mm long, whitish (when fresh). Internodes 0.7–4.5 cm long, angles unwinged. Petioles 6–11 mm long. Leaf blades 20–39 mm long, 9–25 mm wide, coriaceous (when dry), ovate, elliptic or narrowly elliptic, adaxial surface green and partially covered by a thin layer of whitish indumentum, abaxial surface totally concealed by the white lanose indumentum (when fresh), adaxial surface blackened, abaxial surface hidden by the white to pale brown lanose indumentum (when dry), discoloured (when dry), base cuneate to rounded, apex acute to rounded, margin flat, entire and minutely granulose and becoming glabrescent with age, 5-nerved from the base, one pair of acrodromous veins and one pair of tenuous veins close to the margin, tertiaries evident on the abaxial surface, nearly perpendicular to the mid-vein, little reticulate and branching apically, adaxial surface glandular-punctate, usually pruinose and becoming glabrescent with age, abaxial surface densely covered with lanose eglandular trichomes 0.1–0.5 mm long. Inflorescences simple dichasia or reduced to solitary flowers, usually congested. Bracts (including petioles) 1–1.9 cm long, 0.3–0.8 cm wide, 3-nerved, ovate, elliptical or narrowly elliptic, indumentum like that of the major leaves. Bracteoles (at anthesis) with petioles 4–6 mm long and blades eventually linear and rudimentary, blades (when well-developed) 2.2–4.4 mm long, 1.5–2 mm wide, linear to elliptic, base attenuate, apex rounded, margin entire, 1-nerved, indumentum like that of the principal leaves. Flowers 5(–6)-merous, pedicels (at anthesis) 0.7–2.0 mm long. Hypanthia (at anthesis) 5.0–6.5 mm long, 4.5–5.2 mm wide at the torus, campanulate to urceolate, surface hidden by the whitish lanose indumentum composed of eglandular trichomes 0.1–0.5 mm long. Calyx tubes 1.0–2.0 mm long. Calyx lobes (at anthesis) 7.9–9.7 mm long, 2.0–2.7 mm wide at the base, subulate, apex acute, margin entire, (when fresh) surface hidden by the whitish lanose indumentum composed of eglandular trichomes 0.1–0.5 mm long. Petals 19–26 mm long, 10–15 mm wide, white, rarely with pink stains at the apical region, obovate, apex emarginate, margin entire and ciliate with eglandular trichomes 0.1–0.4 mm long at the apical region, both surfaces glabrous. Stamens 10(–12), strongly dimorphic. Larger (antesepalous) stamens 5(–6), filaments 5.2–6.3 mm long, white, pedoconnectives 6.1–7.3 mm long, white to light yellow, appendages 1.9–3.0 mm long, yellow, apex emarginate, thecae (excluding rostra) 2.3–2.7 mm long, vinaceous, oblong, rostra 0.4–0.6 mm long, the circular pores ca. 0.2 mm wide. Smaller (antepetalous) stamens 5(6), filaments 4.0–5.4 mm long, white, pedoconnectives 0.8–1.1 mm long, yellow, inconspicuous appendages ca. 0.1 mm long, yellow, apex emarginate, thecae (excluding rostra) 2.4–2.6 mm long, yellow, oblong, rostra 0.3–0.6 mm long, the circular pores ca. 0.2 mm wide. Ovary 2.5–4.0 mm long, 1.7–2.0 mm wide, globose to cyclindrical, 5-locular. Style 6.5–8.0 mm long, white. Capsules (at maturity) 5.0–8.0 mm long, 5.0–6.0 mm wide, globose, initially enveloped by the hypanthium, torus constricted at the apex, fruiting calyx tubes 2.0–3.1 mm long, fruiting calyx lobes 9.0–11.5 mm long, not thickened. Seeds ca. 0.8 mm long, reniform.

**Figure 23.**
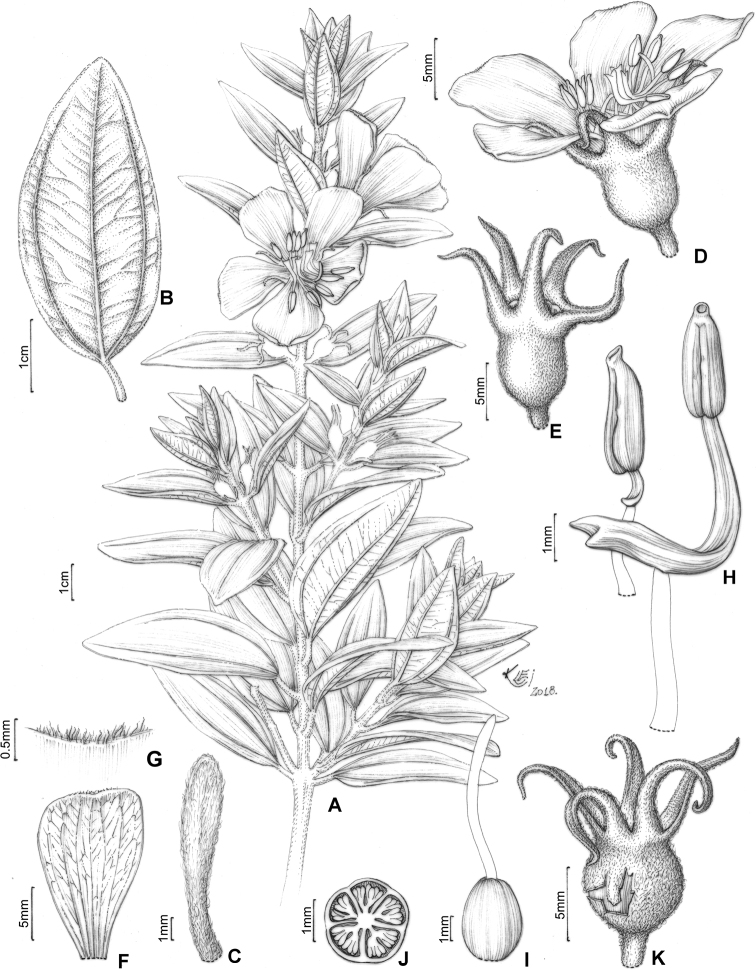
*Microlicialaniflora***A** habit **B** leaf abaxial surface **C** bracteole abaxial surface **D** flower in lateral view **E** flowering hypanthium **F** petal adaxial surface **G** detail of indumentum on the apex of the petal **H** antepetalous (behind) and antesepalous (in front) stamens **I** gynoecium **J** ovary in cross-section **K** capsule enveloped by the hypanthium. Drawn from Almeda et al. 7726 (UEC) and Almeda et al. 9197 (UEC).

#### Distribution, habitat and elevation range.

Endemic to central and southern Minas Gerais (Fig. 19B), mainly at Serra de Ouro Branco, Serra do Itacolomi, Serra do Cipó, Serra da Piedade, Serra da Moeda, Serra do Caraça, Serra do Gandarela, Serra de Lavras Novas, Serra dos Alves, Serra de Itabirito, Serra do Rola-Moça, Serra do Curral, Serra de Taquaril, Serra do Garimpo, Serra do Belo Vale, Diamantina Plateau and mountains in the Municipalities of Brumadinho and Mariana. It occurs on quartzitic or ferrugineous campo rupestre exposed to full sun at elevations between 868 and 1540 m.

#### Ecology.

*Microlicialaniflora* has a symbiotic relationship with arbuscular mycorrhizae (Abrahão et al. 2019). This is likely related to phosphorus and nitrogen acquisition strategies (Abrahão et al. 2019). This species easily colonises degraded campo rupestre areas and has been recommended for ecological restoration (Amaral et al. 2013). According to Pereira (2011), its leaves develop increased asymmetry when damaged by insects. Vasconcelos et al. (2001) reported the use of *M.laniflora* for nesting by the endemic hummingbird, Hyacinth Visorbearer (*Augastesscutatus*), in campo rupestre at the Serra do Cipó.

#### Uses.

Leaf and stem extracts from *Microlicialaniflora* have antimicrobial activity against *Staphylococcusaureus* and *Micrococcusluteus* (Cota et al. 2002; Ventura et al. 2007a).

#### Conservation.

This species is known from more than 100 herbarium specimens making it one of the better sampled species in the *Trembleya* s.s. clade. However, this sampling is geographically biased towards the surroundings of the MG-010 highway, where more than a half of these collections were made. The MG-010 highway is the main road crossing the mountains at south-eastern Serra do Cipó and provides easy access to large populations of *M.laniflora*. The EOO is 19,113.356 km^2^ and the AOO is 248 km^2^. Based on IUCN (2019) recommendations and criteria, we suspect that this species would be classified as Vulnerable (VU): B2ab(iii). Several populations of *M.laniflora* occur within the following conservation units: Parque Estadual Serra do Ouro Branco, Parque Estadual do Biribiri, Parque Estadual de Itacolomi, Monumento Natural Estadual Serra da Piedade, Monumento Natural Estadual Serra da Moeda, Parque Estadual da Serra do Rola-Moça, Parque Nacional da Serra do Gandarela, Parque Nacional da Serra do Cipó and RPPN Serra do Caraça (Natural Heritage Private Reserve), where *M.laniflora* is afforded protection.

#### Recognition and affinities.

*Microlicialaniflora* may be recognised by its branches, abaxial surfaces of the leaves and hypanthia that are densely covered by a lanose indumentum, white petals (rarely flushed with pink) that are 19.0–26.0 mm long and subulate calyx lobes 7.9–9.7 mm long. It appears to be most closely related to *M.pentagona* and *M.chamissoana*. *Microlicialaniflora* differs from *M.pentagona* by the branches, abaxial foliar surfaces and hypanthia densely covered by the lanose indumentum (vs. appearing glabrous, vernicose and minutely granulose), leaves with longer petioles 6.0–11.0 mm long (vs. 0.4–2.5 mm long) and margins entire throughout (vs. serrulate on the upper half), longer hypanthia 5.0–6.5 mm long (vs. 2.5–3.5 mm long) and longer petals 19.0–26.0 mm long (vs. 11.8–13.8 mm long), that are white, rarely flushed with pink (vs. magenta). For additional comparisons, see comments under *M.chamissoana*.

#### Notes.

The four varieties proposed by Cogniaux (1883–1888) were based mainly on differences in petiole length, leaf size and leaf shape. An examination of Cogniuax’s varieties and many additional collections showed these purported differences to be inconsistent with much size overlap in the characters he used to delimit his infraspecific taxa. The ovate leaf shape that Cogniaux (1883–1888) attributed only to var.genuina is present in most of the individuals examined, along with a great deal of variation in leaf and petioles sizes. *Cavalcanti et al. CFSC10628* (UEC), for example, has ovate leaves (a feature attributed only to var.genuina) and well-developed petioles (a feature attributed to the varieties *intermedia*, *grandifolia* and *acutifolia*). This specimen could be identified as var.intermedia since its leaf measurements match those of the protologue (3–4 cm long). However, when comparing the Cavalcanti specimen with the type of var. intermedia (*Gardner 4601*), it is clear that this variety has lanceolate leaves that are very distinct from the ovate leaves of *Cavalcanti et al. CFSC10628*. Characters, such as leaf shape and size, also vary along branches of a single individual and, thus, do not constitute good diagnostic features to delimit varieties of this species.

According to Cogniaux (1883–1888), some of Glaziou’s collections of *M.laniflora* (his numbers *14740* and *14741*) were made “in prov. Rio de Janeiro” [Rio de Janeiro State]. On the other hand, Glaziou (1908: 251) asserts that his number *14740* came from “Serra do Caraça, au Morro do Inficionado” and the number *14741* came from “Campo de São Sebastião, prés Ouro Preto”, both sites located in Minas Gerais State. The labels of *Glaziou 14740* and *14741* from P had annotations of collections’ sites similar to those shown in Glaziou (1908), while a duplicate of *Glaziou 14740* from K has a label that says “14741 the same” and the location site is cited as “Environs de Rio de Janeiro et Ouro Preto”. Due to these contradictions, the cited collections were not used for the geographical distribution summary of *M.laniflora*. The discordant data on Glaziou’s collections of Melastomataceae have been noted by Wurdack (1970) for other collections of Melastomataceae, such as *Clidemia*, *Leandra*, *Macairea*, *Miconia*, *Rhynchanthera*, *Tibouchina* and *Tococa*. This is the first report of dubious geographic information for a species of *Microlicia*. We found similar contradictory information on labels of *Glaziou 14746* which is discussed in the comments under *M.pithyoides*.

#### Specimens examined.

**Brazil. Minas Gerais**: Barão de Cocais Municipality, Serra do Garimpo, Hensold 778 (SPF, US), Semir et al. 28809 (UEC), Souza 1606 (BHCB); Belo Horizonte Municipality, Serra do Taquaril – Serra do Curral, Barreto 6769 (SP), Ducke s.n. (RB [241970]), Ferreira 5544 (HUFU), Roth s.n. (BHCB, CESJ, CTES, ESA, R, RB, SP, UB, UPCB), Morro do Chapéu, Brandão 28574 (HUFU), Brumadinho Municipality, Serra do Rola-Moça, Carmo 4819 (BHCB), Serra da Calçada, Martens 7 (SPF), Martens 383 (SPF), Retiro das Pedras, Carvalho s.n. (HUFU [39992]), Stehmann & Morais 2650 (BHCB); Caeté Municipality, Serra do Gandarela, Damazio 1025 (RB); Catas Altas Municipality, Serra do Caraça, Vasconcelos s.n. (SPF [145870, 145871]); Conceição do Mato Dentro Municipality, Serra do Cipó, Macedo 3758 (S), Martinelli & Tavora 2583 (RB); Diamantina Municipality, “Diamantina Plateau”, Araújo et al. 323 (HUFU, RB, UEC), Brade 13735 (NY, RB, US), Franco et al. 1270 (HUFU), Hatschbach et al. 27400 (K, MBM, UPCB), Hatschbach et al. 68138 (MBM, UPCB), Leitão et al. 17281 (RB, UEC), Lima et al. 49 (SPF), Maguire et al. 49140 (NY), Marques et al. 274 (HUFU), Mello et al. 61 (HUFU), Vauthier 37 (P); Itabira Municipality, Martius 930 (GH, K, NY, P), Torres s.n. (RB [241965]); Itabirito Municipality, Serra do Itabirito, Irwin 19974 (NY), Irwin et al. 19974 (K, NY, US), Krieger 10641 (CESJ, ESA, HUFU, MBM), Lima et al. 1443 (RB), Teixeira s.n. (BHCB [21783], HUFU [19443]); Jaboticatubas Municipality, Serra do Cipó, Goldenberg & Silveira 1573 (UPCB); Mariana Municipality, Collo et al. s.n. (SPF [62806]), Messias et al. 1922 (OUPR, RB); Nova Lima Municipality, Serra do Curral, Nakajima & Romero 3040 (HUFU), Pereira & Pabst 3107 (RB), Sampaio 7194 (BHCB), Williams & Assis 6352 (GH, NY); Ouro Branco Municipality, Serra de Outro Branco, Almeda et al. 7726 (CAS, UEC), Almeda et al. 8395 (CAS, UEC), Alves & Almeida-Lafetá 5573 (R), Alves et al. 6925 (R), Araújo et al. 334 (ESA, RB), Arbo et al. 3906 (CTES, MBM, RB, SPF, UB), Delfini et al. 78 (ESA, HUFU, RB), Forzza et al. 993 (SPF), Nakajima et al. 4547 (HUFU), Paula et al. 136 (HUFU, VIC), Paula et al. 295 (HUFU, VIC), Paula et al. 8 (HUFU, VIC), Pereira & Pabst 2944 (RB), Pirani et al. CFCR11211 (SPF), Rocha et al. 602 (BHCB, NY), Saraiva et al. 85 (OUPR, RB); Ouro Preto Municipality, Barreto & Viégas s.n. (IAC [6389]), Damazio 1540 (NY, US), Ferreira et al. 433 (HUFU), Fontana et al. 2288 (RB), Forzza et al. 6344 (OUPR, RB, UPCB), Forzza et al. 6359 (NY, OUPR, RB, UPCB), Groppo & Ulwin 676 (SPF), Lima et al. 1296 (RB), Meireles et al 1362 (HUEM, UEC), Messias et al. 2151 (OUPR, RB), Rolim et al. 329 (HUFU, RB, VIC), Rolim et al. 386 (RB, VIC), Rolim et al. 61 (HUFU, VIC), Teixeira s.n. (SPF [114173]), Valente et al. 2574 (RB, VIC); Raposos Municipality, Tameirão Neto & Mansur 4874 (BHCB); Rio Acima Municipality [Gandarela], Emygdio 3314 (NY), Emygdio 3353 (NY); Sabará Municipality, Barreto 6771 (BHCB), Barreto 6772 (NY); Santa Bárbara Municipality, Serra do Caraça, Almeda et al. 7753 (CAS, UEC), Almeda et al. 8862 (CAS, UEC), Arbo et al. 4030 (SPF), Barreto 706 (NY, UEC), Fraga et al. 3333 (NY, RB), Fraga et al. 3341 (NY, RB), Marcondes-Ferreira et al. 281 (SPF), Pirani & Yano 696 (CAS, SPF), Pirani et al. 696 (SP, SPF), Rocha et al. 672 (BHCB, RB), Romero et al. 5307 (CAS, UEC), Tales et al. 17 (BHCB, HUFU), Temponi & Vasconcelos s.n. (BHCB [36249], HUFU [19313]), TSMG & Tales 81 (BHCB, HUFU), Valente et al. 1229 (HUFU, VIC), Valente et al. 1230 (HUFU, VIC), Valente et al. 535 (HUFU, VIC); Santana do Riacho Municipality, Serra do Cipó, Almeda et al. 8549 (CAS, UEC), Almeda et al. 9179 (CAS, UEC), Alves et al. 2109 (SPF), Andrade et al. 374 (BHZB, HUFU), Antar et al. 1662 (SPF), Borges et al. 153 (HUEFS, K, NY, SPF), Bruniera et al. 37 (HUFU, SPF), Castro et al. s.n. (HUEM [24842], HUFU [2254]), Ceccantini et al. 3914 (SPF), Chuckr et al. s.n. (HUEM [24776]), Cordeiro et al. CFSC10504 (SPF), Escaramai et al. 65 (SPF), Faria & Mazucato 105 (SPF), Farinaccio et al. 36 (MBM, RB), Fernandes et al. 1468 (BHZB, HUFU), Forero et al. 7700 (SPF), Forero et al. 7831 (SPF), Giulietti et al. CFSC12564 (UEC), Kral et al. 72997 (CEN, SP), Kubo et al. 109 (SPF), Kubo et al. 125 (SPF), Maguire et al. 49016 (NY), Mattos & Rizzini 107 (RB, US), Mattos & Rizzini 480 (RB), Monge et al. 384 (UEC), Ordones et al. 1856 (BHZB, HUFU), Pacifico 185 (HUEM, SPF), Pena & Viana 365 (SPF), Pereira & Pabst 152 (HUEM), Pirani et al. 5082 (NY, SPF), Pires & Braga s.n. (CESJ [21520]), Reginato et al. 1401 (NY, UPCB), Sakuragui & Souza 38 (ESA), Salatino et al. 14 (NY, SPF), Salatino et al. 18 (NY, SPF), Souza et al. 11565 (ESA, RB), Souza et al. 25181 (ESA), Stehmann & Morais 2354 (SPF), Verdi et al. 6501 (RB), Zappi et al. CFSC9353 (SPF); Santana do Riacho Municipality [“Conceição do Mato Dentro”], Serra do Cipó, Marquete et al. 3817 (RB); Santana do Riacho Municipality [“Jaboticatubas”], Serra do Cipó, Joly CFSC2405 (SPF), Joly CFSC82 (SPF), Mantovani 99 (SP, SPF), Semir CFSC2005 (SP, SPF), Semir CFSC4133 (SPF), Semir CFSC5001 (SPF), Semir CFSC5068 (SPF); Serro, Semir et al CFCR230 (SPF); Unknown municipality, Bunge s.n. (P [P005317069]), Claussen 1 (BR, CAS), Claussen 1645 (P), Claussen 332A (BR), Claussen 554 (P), Claussen 640 (BR), Claussen 923 (CAS), Claussen s.n. (K [K00957804, K00957810, K00957811, K00957816], NY [NY00941988], P [P005317066, P005317067, P005317071, P005317072]), Gardner 4601 (BM, G, GH, K, NY, P, R, US), Glaziou 11953 (K), Glaziou 14740 (K, P), Glaziou 14741 (K, P), Glaziou s.n. (P [P005317096]), Gounelle s.n. (P [P005317089]), Lund 135 (C), Martius 930 (BM, G, GH, L, M, P, S), Martius s.n. (P [P005317088]), Netto s.n. (BR [BR0000005520688]), Raben 428 (S), Riedel s.n. (K [K00957817], P [P005317084]), Saint-Hilaire 216 (P), Sellow 1446 (BR, US), Sellow 1727 (P [P00723419]), Sellow s.n. (lectotype: K [K00957812]; probable isolectotypes: K [K00957818], P [P005317063, P005317064, P005317070]), Vauthier s.n. (P [P005317061]).

#### Putative hybrid

**(*M.laniflora* × *M.pentagona*). Brazil. Minas Gerais**: Santana do Riacho Municipality [“Santa Luzia”], Serra do Cipó, Barreto 7026 (BHCB).

### ﻿6. Microlicia parviflora

Taxon classificationPlantaeMyrtalesMelastomataceae

(D.Don) Versiane & R.Romero, Bot. J. Linn. Soc. 197: 54. 2021.

E31D15E3-4DD3-5783-A3EE-73C70E785E54

Fig. 24

Meriania parviflora D.Don, Mem. Wern. Nat. Hist. Soc. 4: 323. 1823. **basionym**. Type: Brazil. *F. Sellow s.n.* (lectotype, designated here: G [G00396696]!; probable isolectotype: P [P05317745]!).
Trembleya heterostemon Mart. & Schrank ex DC., Prod. 3: 126. 1828. **syn. nov.** Type: Brazil. “In Brasiliae subalpinis ad fontes in prov. Minarum Generalium” [Minas Gerais], *C.F.P. Martius 961* (lectotype, designated here: M [M0165884]!; isolectotypes: G [G00310210]!, M [M0165883]!).
Trembleya triflora Mart. & Schrank ex DC., Prod. 3: 126. 1828. **syn. nov.** Type: Brazil. “In sylvis caeduis prope Villam-Riceam prov. Minarum generalium” [Minas Gerais, Ouro Preto], 1827, *C.F.P. Martius s.n.* (lectotype, designated here: M [M0165882]!; isolectotypes: G [G00310209]!, P [P00723390]!; image of isolectotype at P is available at http://coldb.mnhn.fr/catalognumber/mnhn/p/p00723390).
Trembleya paniculata Naudin, Ann. Sci. Nat., Bot. Sér. 3, 2: 154. 1844. Type: Brazil. “In campis circa Juruoca in prov. Minas Geraës” [Minas Gerais], 1816–1821, [catal. D, n° 462] *A. Saint-Hilaire s.n* (lectotype, first-step designated by Martin and Cremers (2007), second-step designated here: P [P00723414]!; isolectotype: P [P00723415]!; image of lectotype is available at http://coldb.mnhn.fr/catalognumber/mnhn/p/p00723414).
Trembleya parviflora (D.Don) Cogn. in Martius et al., Fl. Bras. 14(3): 127. 1883. Type: Based on Merianiaparviflora D.Don.
Trembleya parviflora subsp. heterostemon (Mart. & Schrank ex DC.) Cogn. in Martius et al., Fl. Bras. 14(3): 128. 1883. **syn. nov.** Type: Based on Trembleyaheterostemon Mart. & Schrank ex DC.
Trembleya parviflora subsp. triflora (Mart. & Schrank ex DC.) Cogn. in Martius et al., Fl. Bras. 14(3): 129. 1883. **syn. nov.** Type: Based on Trembleyatriflora Mart. & Schrank ex DC.
Trembleya parviflora var. angustifolia Cogn. in Martius et al., Fl. Bras. 14(3): 128. 1883. **syn. nov.** Type: Brazil. “In prov. Rio de Janeiro ad Serra dos Orgâos” [Rio de Janeiro], 1838, *G. Gardner 379* (lectotype, designated here: P [P005317041]!; isolectotypes: BR [BR0000005520404]!, NY [NY00245857-online image]!, US [US00623960]!; image of lectotype is available at http://coldb.mnhn.fr/catalognumber/mnhn/p/p05317041).
Trembleya parviflora var. denticulata Cogn. in Martius et al., Fl. Bras. 14(3): 129. 1883. **syn. nov.** Type: Brazil. “In prov. S. Paulo ad Paitura” [São Paulo], 1846, *Prates s.n.* (lectotype, designated here: P [P00723407]!; isolectotypes: P [P00723406]!, P [P00723408]!; image of lectotype is available at http://coldb.mnhn.fr/catalognumber/mnhn/p/p00723407).
Trembleya parviflora var. farinacea Cogn. in Martius et al., Fl. Bras. 14(3): 128. 1883. **syn. nov.** Type: Brazil. “In campo sicco apricot vel umbroso ad Caldas prov. Minas Geraës” [Minas Gerais, Caldas], *H. Mosen 1971* (lectotype, designated here: R [R000166846]!; isolectotypes: C [C10015104]!, P [P005317106]!; image of isolectotype at P is available at http://coldb.mnhn.fr/catalognumber/mnhn/p/p05317106).
Trembleya parviflora var. latifolia Cogn. in Martius et al., Fl. Bras. 14(3): 128. 1883. **syn. nov.** Type: Brazil. “In prov. Rio de Janeiro ad Serra dos Orgâos”, 1838, *G. Gardner 380* (lectotype, designated here: P [P00723401]!; isolectotypes: BR [BR0000005225798]!, F [F0064040F]!, G [G00359408]!, G [G00368014-online image]!, NY [NY00217747]!, NY [NY00245859]!, P [P00723402]!, S [S05-3227]]!, US [US00623963]!; image of lectotype is available at http://coldb.mnhn.fr/catalognumber/mnhn/p/p00723401).
Trembleya parviflora var. martii Cogn. in Martius et al., Fl. Bras. 14(3): 129. 1883. **syn. nov.** Type: Brazil. “In prov. Minas Geraës ad Serra do Ouro Preto” [Minas Gerais, Ouro Preto], *C.F.P. Martius 931* (lectotype, designated here: P [P005317103]!; isolectotypes: BM, G [G00368009]!, G [G00318575]!, M [M0165879]!, M [M0165880]!, MO [MO-2267366]!, P [P005317750]!, P [P005317751]!; image of lectotype is available at http://coldb.mnhn.fr/catalognumber/mnhn/p/p05317103).
Trembleya parviflora var. multiflora Cogn. in Martius et al., Fl. Bras. 14(3): 129. 1883. **syn. nov.** Type: Brazil. “Ayuruoca, Minas Gerais’”, 17 April 1878, *A.F.M. Glaziou 9454* (lectotype, designated here: R [R000009197]!; isolectotypes: BR [BR0000005226733]!, BR [BR0000005226405]!, BR [BR0000005227068]!, C [C10015105-online image]!, G [G00368008]!, G [G00318573-online image]!, S [S-0912956-online image]!, P [P005317099]!, P [P005317057]!; image of isolectotype at P is available at http://coldb.mnhn.fr/catalognumber/mnhn/p/p05317099).
Trembleya parviflora var. parvifolia Cogn. in Martius et al., Fl. Bras. 14(3): 129. 1883. **syn. nov.** Type: Brazil. “In prov. Minas Geraës ad Rio das Pedras” [Minas Gerais], *F. Sellow 1154* (lectotype, designated here: US [US00623966-online image]!; image of lectotype is available at http://n2t.net/ark:/65665/392812a8c-782b-431c-b2fc-3644dcfad16e).
Trembleya parviflora var. selloana Cogn. in Martius et al., Fl. Bras. 14(3): 128. 1883. **syn. nov.** Type: Brazil. “In prov. Minas Gerais” [Minas Gerais], *F. Sellow 5278* (lectotype, designated here: P [P00723410]!; image of lecotype is available at http://coldb.mnhn.fr/catalognumber/mnhn/p/p00723410).
Trembleya parviflora var. tomentosa Cogn. in Martius et al., Fl. Bras. 14(3): 128). **syn. nov.** Type: Brazil. “In prov. Rio de Janeiro ad Serra dos Orgâos” [Rio de Janeiro], *J.B.A. Guillemin 946* (lectotype, designated here: P [P005317767]!; isolectotypes: G [G00368013]!, P [P00723409]!; image of lectotype is available at http://coldb.mnhn.fr/catalognumber/mnhn/p/p05317767).
Trembleya parviflora var. triflora (Mart. & Schrank ex DC.) Cogn. in Martius et al., Fl. Bras. 14(3): 129. 1883. **syn. nov.** Type: Based on Trembleyatriflora Mart. & Schrank ex DC.
Trembleya parviflora var. valtheri Cogn. in Martius et al., Fl. Bras. 14(3): 128. 1883. **syn. nov.** Type: Brazil. “In prov. Minas Geraës” [Minas Gerais], 1833, *M. Vauthier 43* (lectotype, designated here: P [P00723403]!; isolectotypes: BR [BR0000005226375]!, G [G00368012]!, G [G00318569]!, P [P00723404]!, P [P00723405]!; image of lectotype is available at http://coldb.mnhn.fr/catalognumber/mnhn/p/p00723405).
Trembleya parviflora var. vulgaris Cogn. in Martius et al., Fl. Bras. 14(3): 128. 1883. **syn. nov.** Type: Brazil. “In prov. Minas Geraës” [Minas Gerais], 1840, *G. Gardner 4602* (lectotype, designated here: R [R000168427]!, isolectotypes: NY [NY00245860]!, NY [NY00245861]!, US; image of isolectotype at NY is available at http://sweetgum.nybg.org/science/vh/specimen_details.php?irn=535234).
Trembleya parviflora var. warmingii Cogn. in Martius et al., Fl. Bras. 14(3): 128. 1883. **syn. nov.** Type: Brazil. “In prov. Minas Gerais ad Lagoa Santa” [Minas Gerais, Lagoa Santa], *E. Warming s.n.* (lectotype, designated here: P [P005317116]!; isolectotypes: BR [BR0000005520732]!; C [C10015107-online image]!; image of lectotype is available at http://coldb.mnhn.fr/catalognumber/mnhn/p/p05317116).
Trembleya parviflora var. widgrenii Cogn. in Martius et al., Fl. Bras. 14(3): 128. 1883. **syn. nov.** Type: Brazil. “In prov. Rio de Janeiro” [Rio de Janeiro], *Widgren s.n.* (lectotype, designated here: P [P005317115]!; isolectotypes: BR [BR0000005227044]!, PH [PH00027744]!, S [S-053231]!; image of lectotype is available at http://coldb.mnhn.fr/catalognumber/mnhn/p/p05317115).
Trembleya parviflora var. heterophylla Cogn. in de Candolle & de Candolle [A.D.C. & C.DC.], Monogr. Phan. 7: 75. 1891. **syn. nov.** Type: Brazil. “In prov. Rio de Janeiro, Nova Friburgo” [Rio de Janeiro, Nova Friburgo], 31 July 1877, *A.F.M. Glaziou 16778* (lectotype, designated here: R [R000009196]!; isolectotypes: C [C10015106]!, G [G00368010]!, G [G00368011]!, L [L0056325]! P [P00723411]!, P [P00723412]!, P [P00723413]!; image of isolectotype at P is available at http://coldb.mnhn.fr/catalognumber/mnhn/p/p00723411).

#### Description.

Erect shrubs or treelets 0.7–4.0 m tall. Branchlets quadrangular, always glandular-punctate and usually pruinose-granulose, eventually sparsely to densely covered with gland-tipped trichomes 0.1–0.9 mm long, rarely covered with rigid hyaline eglandular trichomes 0.1–0.5 mm long, light green (when fresh). Internodes 0.6–3.8 cm long, angles unwinged. Petioles 1–15 mm long. Leaf blades 12–117 mm long, 3–38 mm wide, papyraceous (when dry), elliptic to narrowly elliptic or lanceolate, rarely obovate, adaxial surface green and partially covered by a thin layer of whitish indumentum, abaxial surface totally concealed by the white lanose indumentum (when fresh), adaxial surface blackened and abaxial surface pale green to pale brown (when dry), discoloured (when dry), base attenuate, apex acute to obtuse, margin flat or slightly revolute, entire to slightly serrulate and glabrescent, granulose or granular-punctate or ciliate with gland-tipped trichomes 0.1–0.9 mm long, 5-nerved from the base, one pair of acrodromous veins and one pair of tenuous veins close to the margin, tertiaries evident on the abaxial surface, nearly perpendicular to the mid-vein, reticulate and randomly branching, adaxial surface glandular-punctate, usually pruinose and becoming glabrescent with age, abaxial surface glandular-punctate and usually pruinose, eventually sparsely to densely covered with gland-tipped or eglanduar trichomes 0.1–0.9 mm long. Inflorescences simple or compound dichasia consisting of with biparous cymes, not congested. Bracts (including petioles) 0.7–1.6 cm long, 0.1–0.6 cm wide, 3-nerved, elliptic to narrowly elliptic, indumentum like that of the principal leaves. Bracteoles (at anthesis) with petioles 0.9–1.9 mm long, blades 2.2–3.8 mm long, 0.5–1.0 mm wide, narrowly elliptic to oblanceolate, base attenuate, apex acute to obtuse, margin entire, 1–3-nerved, indumentum like that of the principal leaves. Flowers 5(–6)-merous, pedicels (at anthesis) 1.1–2.0 mm long. Hypanthia (at anthesis) 1.9–2.8 mm long, 1.5–2.2 mm wide at the torus, campanulate to urceolate, light green, sometimes with reddish stains (when fresh), externally always glandular-punctate and usually pruinose, eventually sparsely to densely covered with gland-tipped trichomes 0.1–0.9 mm long, rarely covered with rigid hyaline eglandular trichomes 0.1–0.5 mm long. Calyx tubes 0.2–0.7 mm long. Calyx lobes (at anthesis) 0.7–2.5(–3.0) mm long, 0.9–1.5 mm wide at the base, triangular, apex acute, acuminate or apiculate, margin entire, (when fresh) light green or reddish, externally like the hypanthia. Petals 4.5–8.1 mm long, 3.0–4.9 mm wide, white, usually flushed with pink at the base and around the veins, rarely entirely pink, obovate, apex emarginate, rounded or acute, margin entire, glabrous or ciliate with eglandular or gland-tipped trichomes 0.1–0.4 mm long at the apical region, both surfaces glabrous. Stamens 10(–12), strongly dimorphic. Larger (antesepalous) stamens 5(–6), filaments 3.5–4.0 mm long, white or pink, pedoconnectives 3.0–4.0 mm long, white or pink, appendages 0.7–1.5 mm long, yellow, apex emarginate to bilobate, thecae (excluding rostra) 0.8–1.3 mm long, red to vinaceous, oblong, rostra 0.2–0.4 mm long, the circular pores 0.1–0.2 mm wide. Smaller (antepetalous) stamens 5(6), filaments 2.0–3.1 mm long, white or pink, pedoconnectives 0.2–0.5 mm long, white or pink, inconspicuous appendages ca. 0.1 mm long, yellow or pink, apex emarginate, thecae (excluding rostra) 0.8–1.3 mm long, yellow, oblong, rostra 0.2–0.4 mm long, the circular pores ca. 0.2 mm wide. Ovary 1.8–2.2 mm long, 1.6–1.8 mm wide, globose, 5-locular. Style 3.0–3.6 mm long, white or pink. Capsules (at maturity) 2.9–5.0 mm long, 2.5–4.0 mm wide, globose, initially enveloped by the hypanthium, torus constricted at the apex, fruiting calyx tubes 0.2–1.0 mm long, fruiting calyx lobes 1.2–2.9(–3.5) mm long, not thickened. Seeds 0.3–0.6 mm long, reniform.

**Figure 24.**
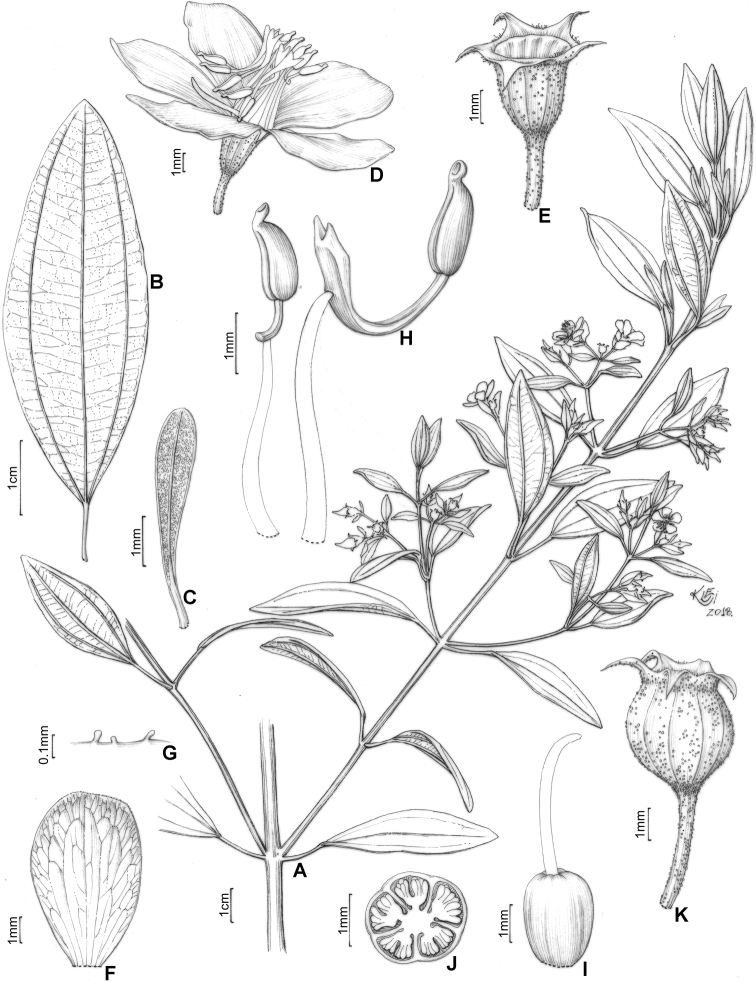
*Microliciaparviflora***A** habit **B** leaf abaxial surface **C** bracteole abaxial surface **D** flower in lateral view **E** flowering hypanthium and pedicel **F** petal adaxial surface **G** detail of indumentum on the apex of the petal **H** antepetalous (left) and antesepalous (right) stamens **I** gynoecium **J** ovary in cross-section **K** capsule enveloped by the hypathium. Drawn from Souza-Buturi 328 (UEC) and Matsumoto et al. 428 (UEC).

#### Representative specimens

**(one specimen selected for each municipality of occurrence). Bahia**: Abaíra, Ganev 879 (HUEFS, NY, SPF); Andaraí, Orlandi et al. 778 (MBM); Lençóis, Carvalho 1086 (CEPEC, RB, SPF); Miguel Calmon, Guedes et al. 12092 (ALCB); Morro do Chapéu, Hage et al. 2317 (CEPEC, MBM, RB); Mucugê, Roque 2869 (ALCB); Palmeiras, Bautista 1347 (CEPEC); Piatã, Ganev 965 (HUEFS, SPF); Ribeirão do Largo, Carvalho 6986 (CEPEC, NY); Rio de Contas, Carvalho et al. 6651 (CEPEC); Seabra, Irwin et al. 31141 (NY); Utinga, Samento & Bautista 859 (RB); Wenceslau Guimarães, Goldenberg & Michelangeli 2093 (UPCB). **Distrito Federal**: Brasília, Azevedo et al. 675 (CAS); Taguatinga, Irwin et al. 8149 (SP). **Espírito Santo**: Domingos Martins, Pereira 309 (CEPEC, SP); Dores do Rio Preto, Monge et al. 2595 (UEC); Fundão, Kollmann 231 (RB, UPCB); Itaguaçu, Brade et al. 18205 (RB); Iúna, Fontana et al. 7678 (UPCB); Marechal Floriano, Hatschbach et al. 74974 (FURB, HCF, MBM); Santa Rita de Jacutinga, Krieger 8857 (MBM); Santa Teresa, Martinelli et al. 10932 (RB). **Goiás**: Alto Paraiso de Goiás, Marquete et al. 2332 (RB, US); Cocalzinho de Goiás, Versiane et al. 305 (HUFU, RB); Corumbá de Goiás, Irwin et al. 34521 (NY); Cristalina, Monteiro 78 (RB, SPF, UPCB); Pirenópolis, Delprete 9205 (RB). **Minas Gerais**: Aiuruoca, Glaziou 9454 (P); Alagoa, Guiamarães 333 (RB); Alto Caparaó, Hatschbach & Guimarães 55455 (MBM); Baependi, Souza et al. 1013 (CESJ, MBM); Barão de Cocais, Fontana 2309 (RB, UPCB); Barbacena, Barreto 4621 (SP); Barroso, Assis et al. 520 (MBM); Belo Horizonte, Vidal s.n. (CEN [46402]); Boa Esperança, Silva s.n. (UPCB [52391]); Bom Jardim de Minas, Krieger et al. 24423 (SPF); Brumadinho, Martens 524 (SPF); Buenópolis, Davis et al. 2298 (UEC); Buritizeiro, Hatschbach et al. 75995 (MBM); Caeté, Paula & Grandi s.n. (BHCB [7956], MBM [178326]); Carandaí, Costa 451 (RB); Carangola, Leoni 1 (SPF); Carmésia, Stehmann 2532 (ESA); Carrancas, Sobral et al. 14085 (RB); Catas Altas, Oliveira et al. 508 (BHCB, RB); Conceição do Ibitipoca, Oliveira 25197 (CESJ, RB); Conceição do Mato Dentro, Guarçoni & Sartori 1367 (HUFU); Coromandel, Brandão 14509 (HUFU); Cristália, Hatschbach 41498 (MBM, US); Delfinópolis, Romero & Nakajima 3432 (HUFU); Diamantina, Hatschbach & Pelanda 28014 (MBM); Espera Feliz, Foster & Leoni 64 (ESA); Estrela do Sul, Costa et al. 60 (HUFU); Extrema, Yamamoto 1549 (UEC); Gouveia, Hatschbach 27277 (MBM); Grão Mogol, Cavalcanti CFCR8314 (SPF); Itabira, Faria et al. 1332 (HUFU); Itabirito, Brandão 22298 (HUFU); Itamonte, Batista & Naves 405 (UEC); João Pinheiro, Heringer 8544/736 (UB, US); Joaquim Felício, Cavalcanti et al. CFCR8035 (SPF, UEC); Lagoa Santa, Warming s.n. (BR [BR0000005520732], C [C10015107], P [P005317116]); Lavras, Avezum & Almeida 10 (SPF); Lima Duarte, Heluey & Castro 108 (RB); Manhuaçu, Hatschbach & Silva 49393 (HUEM, MBM); Mariana, Lombardi 4045 (BHCB, ESA); Moeda, Silva & Grandi 6627 (HUFU); Nova Lima, Williams & Assis 7274 (SP); Ouro Branco, Delfini et al. 97 (ESA, RB); Ouro Preto, Colletta 161 (SPF), Martius 931 (BM, G, M, MO, P); Paracatu, Bovini & Barros 3235 (RB); Passa Quatro, Meireles et al. 1766 (RB, UEC); Patrocínio, Farah et al. 580 (ESA, HUEM); Perdizes, Mendes & Araújo 970 (SPF); Piuhmhi, Emygdio 3613 (NY, R); Poços de Caldas, Oliveira 1013 (US); Prados, Sobral et al. 12797 (UPCB); Presidente Soares, Hatschbach et al. 55434 (MBM); Rio Acima [Gandarela], Emygdio 3312 (NY, R); Rio Pardo de Minas, Sevilha et al. 7075 (CEN); Rio Preto, Barros & Feteira 1625 (RB); Rio Vermelho, Mello-Silva et al. CFCR7836 (SPF); Rosário de Limeira, Marcolino 222 (RB); Sabará, Barreto 6759 (BHCB, SP); Sacramento, Romero et al. 2155 (MBM); Santa Bárbara, Barreto 6757 (BHCB, SP); Santa Bárbara do Monte Verde, Pivari 15 (MBM); Santana de Pirapama, Zappi et al. 2504 (SPF); Santana do Riacho, Pacifico 191 (HUEM); Santos Dumont, Mello-Silva et al. 1217 (SPF); São Gonçalo do Rio Preto, Foresto et al. 59 (SPF); São Gonçalo do Sapucaí, Hatschbach 26964 (MBM); São João Batista da Glória, Kinoshita et al. 43767 (HUEM); São João del Rei, Barreto 4654 (SP, US); São Roque de Minas, Pacifico 413 (CAS, HUEM); São Tomé das Letras, Valente & Azevedo 57 (RB); Serro, Almeda et al. 9076 (CAS, UEC); Tapira, Salgado 167 (RB); Tiradentes, Alves 600 (R); Tombos, Fraga & Saddi 1786 (CEPEC, RB); Uberlândia, Romero & Nakajima 3021 (HUFU); Unknown municipality in Minas Gerais State, Gardner 4602 (NY, R, US), Martius 961 (G, M), Mosen 1971 (C, P, R), Saint-Hilaire s.n. [D462] (P [P00723414], P [P00723415]), Sellow 1154 (US), Sellow 5278 (P), Vauthier 43 [part] (BR, G, P), Widgren 967 (BR). **Paraná**: Adrianópolis, Camargo et al. 109 (UPCB); Antonina, Hatschbach & Guimarães 56167 (MBM); Araponga, Caiafa & Umbelino 172 (UPCB, VIC); Arapoti, Hatschbach 6908 (MBM); Balsa Nova, Hatschbach et al. 42967 (MBM); Bocaiúva do Sul, Ribas et al. 6769 (MBM, UPCB); Campina Grande do Sul, Brotto & Vieira 1921 (MBM); Colombo, Hatschbach 647 (MBM, RB); Jaguariaíva, Ribas et al. 8528 (MBM); Ortigueira, Silva et al. 6478 (MBMB, UNOP); Palmeira, Hatschbach 2775 (MBM); Piraí do Sul, Goldenberg et al. 1652 (NY, UPCB); Ponta Grossa, Silva & Koch 7610 (MBM); Rio Branco do Sul, Silva & Abe 2310 (MBM); Sengés, Hatschbach 39954 (MBM); Tibagi, Kirizawa 3665 (SP); Tunas do Paraná, Brotto 2600 (MBM, RB); Ventania, Estevan et al. 617 (UPCB). **Rio de Janeiro**: Bom Jardim, Hottz et al. 302 (MBM, RB); Duque de Caxias, Lima et al. 8445 (RB); Itatiaia, Baumgratz et al. 1127 (MBM, SPF); Miguel Pereira, Wängler & Ferreira 1352 (RB); Nova Friburgo, Forzza et al. 3427 (RB); Petrópolis, Vieira & Yamamoto 26186 (FUEL, RB, UEC); Resende, Eiten & Eiten 7305 (NY, P, RB, SP, US); Rio Claro, Moutinho et al. 76 (RB); Santa Maria Madalena, Lima 400 (US); Teresópolis, Duarte & Brade 1155 (RB); Unknown municipality in Rio de Janeiro state, Gardner 379 (BR, NY, P, US), Gardner 380 (BR, F, G, NY, P, S, US), Guillemin 921 (P), Guillemin 946 (G, P), Widgren s.n. (BR [BR0000005227044], PH-27744, S-53231). **São Paulo**: Apiaí, Souza et al. 6098 (ESA, RB); Bananal, Martinelli 19464 (RB); Bom Sucesso de Itararé, Aguiar 102 (MBM); Brotas, Queiroz 2808 (CEPEC); Campos do Jordão, N. da Cruz 135 (CAS, MBM); Cunha, Mamede et al. 666 (RB, SP); Eldorado, Pastore et al. 685 (RB); Iporanga, Souza et al. 5934 (SPF); Itapeva, Baitello et al. 2136 (UPCB); Itirapina, Tannus 760 (HUFU); Lavrinhas, Caddah et al. 636 (UPCB); Mogi Mirim, Hoehne 20521 (NY, SP); Pedregulho, Polisel et al. 176 (UPCB); Pindamonhangaba, Nicolau et al. 2212 (SP); Piquete, Gonçalves et al. 172 (RB); Rio Claro, Loefgren 557 (BHCB, SP); Santo André, Kirizawa et al. 2132 (SP); São Bernardo do Campo, Kuhlmann 4381 (SP); São Carlos, Eiten et al. 3019 (SP, US); São José do Barreiro, Handro 790 (NY); São José dos Campos, Mimura 479 (SP, US); São Paulo, Beraldo & França 85 (SPF); Ubatuba, Souza et al. 1108 (UPCB); Unknown municipality in São Paulo State, Prates s.n. (P [P00723407], P [P00723406, P [P00723408]). **Unknown state**: Bunbury s.n. (BR [BR0000005520374]), Glaziou 12704 (BR), Glaziou 16679 (BR), Glaziou 16778 (C, L, P), Glaziou 2579 (BR, P), Glaziou 8680 (BR, P), Glaziou 9454 (BR, C, G, P, R, S), Martius 989 (BR), Raben 409 (BR), Riedel s.n. (BR [BR0000005520367]), Sellow s.n. (lectotype: G [G00396696], probable isolectotype: P [P05317745]).

#### Distribution, habitat and elevation range.

**Figure 25.**
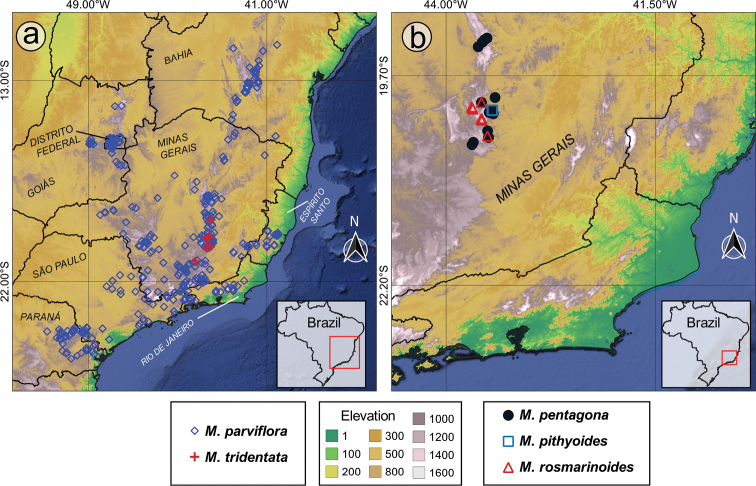
Geographic distribution of species of the *Trembleya* s.s. clade of *Microlicia***A** distributions of *M.parviflora* and *M.tridentata***B** distributions of *M.pentagona*, *M.pithyoides* and *M.rosmarinoides*.

Endemic to Brazil in the States of Bahia, Minas Gerais, Goiás, Distrito Federal, Espírito Santo, Rio de Janeiro, São Paulo and Paraná (Fig. 25A). *Trembleyaparviflora* is common in gallery forests surrounding campo rupestre, Cerrado, campo sujo, campo limpo. campos de altitude, Veredas (palm swamps), and margins of roads throughout Cerrado and Atlantic forest fragments, usually exposed to full sun, at elevations between 560 and 2223 m.

#### Ecology.

*Microliciaparviflora* is the most widely distributed species in the clade and the most studied from an ecological perspective. Giotto (2015) investigated plant occupation in palm swamps with very dense populations of *M.parviflora*, where seedlings of this species corresponded to 78% of the total seed bank. Silva (2003) reported a population density of 1.47 individuals per square metre in these areas. This elevated local dominance of *M.parviflora* negatively affects the overall plant species richness of these regions and is apparently favoured by reduction in humidity (Giotto 2015), although *M.parviflora* occurs with large populations in both wet and dry areas (Melo 2013). This species is strongly recommended for ecological restoration (Amaral et al. 2013) because it readily colonises degraded campo rupestre. Leaf extracts of *M.parviflora* proved to have allelopathic properties, inhibiting root and shoot growth of *Sesamumindicum* (Borghetti et al. 2005). According to Pereira (2011), leaves of *M.parviflora* develop increased asymmetry when damaged by insects.

#### Conservation.

This is the most abundant species in the *Trembleya* s.s. clade, with more than 1000 collections currently housed in herbaria. The EOO is 1,152,078.308 km^2^ and the AOO is 1,640 km^2^. Populations of *M.parviflora* are found in all conservation units that protect the remaining species of the clade. As it occurs in large and dense populations, some have claimed that *M.parviflora* has the potential to become an invasive species (e.g. Silva 2003). We are not aware of records of this species occupying regions outside its natural distributional range. Based on the IUCN (2019) recommendations and criteria, we recommend that a conservation status of Least Concern (LC) be assigned to this species.

#### Recognition and affinities.

*Microliciaparviflora* can be distinguished from its congeners by its leaves that are 5-nerved from the base, the abaxial surface glandular-punctate, sometimes covered with an indumentum of pedicellate trichomes (never dense enough to conceal the epidermis), three to many-flowered inflorescences, triangular calyx lobes 0.7–2.5(–3) mm long, petals white or pink and dimorphic stamens. *Microliciapentagona* and *M.trembleyiformis* are somewhat similar to *M.parviflora* in leaf shape and venation and stamen morphology. *Microliciaparviflora* differs from *M.pentagona* by its leaves with tertiaries evident (vs. little evident), moderately reticulate and randomly branching (vs. parallel and/or branching apically), inflorescences (vs. solitary flowers) and triangular calyx lobes 0.7–2.5(–3) mm long (vs. subulate and 6.2–8.5 mm long) that are tenuous in fruit (vs. thickened). In turn, *M.parviflora* differs from *M.trembleyiformis* by the unwinged branchlets (vs. with narrow wings ca. 0.2 mm wide), flowers disposed in inflorescences (vs. flowers solitary), triangular calyx lobes (vs. narrowly-triangular) and consistently 5-locular ovaries (vs. 3–5-locular). For a comparison between *M.parviflora* and *M.altoparaisensis*, see notes under the latter species.

#### Notes.

The morphological variation found in *M.parviflora* is mainly related to leaf blade shape (varying from elliptic to ovate and lanceolate), indumentum on branches and abaxial leaf surface (always glandular-punctate, usually pruinose, eventually sparsely to densely covered with gland-tipped or eglandular trichomes 0.1–0.9 mm long) and inflorescence development (three- to many-flowered). The petals are usually white flushed with pink at the base, although some populations have entirely white petals, for example, in Serra de Carrancas (Matsumoto and Martins 2005) and Serra Negra (Justino et al. 2016) in Minas Gerais. Based on descriptions of *M.parviflora* in local floras, magenta-flowered and white-flowered variants of this species occur together in Poços de Caldas and Paque Estadual do Ibitipoca, both in Minas Gerais. In the Serra da Canastra, Romero (2000: 283) recognised a variant of *M.parviflora* as a distinct entity (*Trembleya* sp.) characterised by tertiaries more evident, trichomes on the abaxial leaf surface with 3–4-lobed heads, inflorescence internodes 7–9 mm long and calyx lobes 2.5–3 mm long. Several other modal extremes were designated as types of infraspecific taxa by Cogniaux (1883–1888, 1891), for example, the specimen *Prates s.n.* (P [P00723407]; type of var.denticulata) has more conspicuous serrations on the leaf margin, whereas *Guillemin 946* (P; type of var.tomentosa) has a denser indumentum on the branchlets. However, none of the mentioned variations is correlated with other characteristics in a meaningful way.

The 15 varieties of *M.parviflora* proposed by Cogniaux (1883–1888, 1891) were based exclusively on differences in leaf blade size, shape and apex and degree of inflorescence development. Based on a comprehensive sampling and analysis of representative collections from throughout the range of the species, it is clear that Cogniaux’s infraspecific taxonomy is artificial and untenable. In fact, many of the specimens examined had leaf sizes that do not fit into any of the infraspecific taxa proposed by Cogniaux (1883–1888, 1891). For example, the specimen *Kinoshita et al. 10/19* (UEC) has leaves 1.5–8 cm long and could fit all varieties proposed, based on this feature. The features that Cogniaux (1883–1888) used to circumscribe the two subspecies are also imprecise and not diagnostic. After analysing several representative collections of *M.parviflora*, we did not find any specimen with clearly tetragonal and totally glabrous branches, both purported diagnostic features of M.parviflorasubsp.triflora. Thus, given the highly polymorphic nature of *M.parviflora*, we do not recognise any infraspecific taxa in this species.

Like *Microliciacataphracta* (Mart. & Schrank ex DC.) Versiane & R.Romero [= *Lavoisieraimbricata* (Thunb.) DC.], *M.parviflora* fits all the six criteria enumerated by Cronk (1998) to be considered as an ochlospecies (see Martins and Almeda 2017). Besides sharing outstanding levels of morphological variation, *M.cataphracta* and *M.parviflora* also have similar distributional ranges and usually occur in large populations. The reproductive biology, population characteristics, chemical composition and other ecological aspects of *M.parviflora* are summarised in other sections of this paper.

Martin and Cremers (2007) examined a supposed holotype of *Trembleyapaniculata* at P. However, we found two duplicates of the same collection used by Naudin (1844) to describe *Trembleyapaniculata* (*Saint-Hilaire* s.n., P [P00723414], P [P00723415]). Thus, we designated one of these sheets as the lectotype (P [P00723414]) and the other as an isolectotype (P [P00723415]) for *Trembleyapaniculata*.

### ﻿7. Microlicia pentagona

Taxon classificationPlantaeMyrtalesMelastomataceae

(Naudin) Versiane & R.Romero, Bot. J. Linn. Soc. 197: 54. 2021.

B5773F41-A9E8-5ED2-AB07-45232617028E

Fig. 26

Trembleya pentagona Naudin, Ann. Sci. Nat., Bot. Sér. 3, 2: 154. 1844. **basionym**. Type: Brazil. “In montibus vulgo Serra d’Ouro Branco, provincia Minas-Geraes” [Minas Gerais, Ouro Branco], *A. Saint-Hilaire s.n.* (lectotype, first-step designated by Martin and Cremers (2007), second-step designated here: P [P00723399]!; isolectotype: P [P00723398]!; image of lectotype is available at http://coldb.mnhn.fr/catalognumber/mnhn/p/p00723399).

#### Description.

Erect shrubs or treelets 0.5–1.5 m tall. Branchlets quadrangular, appearing glabrous, vernicose and minutely granulose, vinaceous (when fresh). Internodes 0.4–2.5 cm long, angles unwinged. Petioles 1.8–2.4 mm long. Leaf blades 12–40 mm long, 3–15 mm wide, chartaceous to coriaceous (when dry), elliptic, ovate, narrowly elliptic or linear, both surfaces green (when fresh), adaxial surface blackened and abaxial surface pale green to pale brown (when dry), discoloured (when dry), base cuneate to attenuate, apex rounded to acute, margin flat, entire along the basal half, serrulate on the upper half and minutely granulose and becoming glabrescent with age, 5-nerved from the base, one pair of acrodromous veins and one pair of tenuous veins close to the margin, tertiaries little evident on the abaxial surface, nearly perpendicular to the mid-vein, parallel or little reticulate and branching apically, adaxial surface glabrous to minutely granulose, vernicose, abaxial surface glabrous to minutely granulose, vernicose. Inflorescences reduced to solitary flowers apically on the branches. Bracts absent. Bracteoles (at anthesis) with petioles 1.8–3.0 mm long, blades 3.0–6.5 mm long, 1.1–3.6 mm wide, elliptic, ovate or lanceolate, base attenuate to cuneate, apex acute to obtuse, margin entire, 3-nerved, indumentum like that of the principal leaves. Flowers (4–)5-merous, pedicels (at anthesis) 0.8–2.0 mm long. Hypanthia (at anthesis) 2.5–3.5 mm long, 2.7–3.0 mm wide at the torus, campanulate to urceolate, light green or reddish (when fresh), externally glabrous, minutely granulose, vernicose. Calyx tubes 0.2–0.4 mm long. Calyx lobes (at anthesis) 6.2–8.5 mm long, 1.8–2.2 mm wide at the base, subulate, apex acute, margin entire, (when fresh) light green or reddish, externally like the hypanthia. Petals 11.8–13.8 mm long, 7.1–9.5 mm wide, magenta, obovate, apex shortly acuminate, margin entire and glabrous, both surfaces glabrous. Stamens (8)10, strongly dimorphic. Larger (antesepalous) stamens (4)5, filaments 4.0–5.0 mm long, pink, pedoconnectives 5.3–5.8 mm long, pink, appendages 1.2–1.6 mm long, yellow, apex emarginate to bilobate, thecae (excluding rostra) 1.2–2.0 mm long, vinaceous, oblong, rostra 0.5–0.7 mm long, the circular pores ca. 0.2 mm wide. Smaller (antepetalous) stamens (4)5, filaments 3.9–4.4 mm long, pink, pedoconnectives 1.0–1.6 mm long, yellow, inconspicuous appendages ca. 0.1 mm long, yellow, apex emarginate, thecae (excluding rostra) 1.6–2.0 mm long, yellow, oblong, rostra 0.5–0.6 mm long, the circular pores ca. 0.2 mm wide. Ovary 2.5–2.7 mm long, 2.4–2.6 mm wide, globose, 5-locular. Style 6.0–6.6 mm long, magenta. Capsules (at maturity) 4.5–5.5 mm long, 5.0–6.0 mm wide, globose, initially enveloped by the hypanthium, torus constricted at the apex, fruiting calyx tubes 0.2–0.4 mm long, fruiting calyx lobes 6.5–11.0 mm long, stout, thickened. Seeds 0.5–0.7 mm long, reniform.

**Figure 26.**
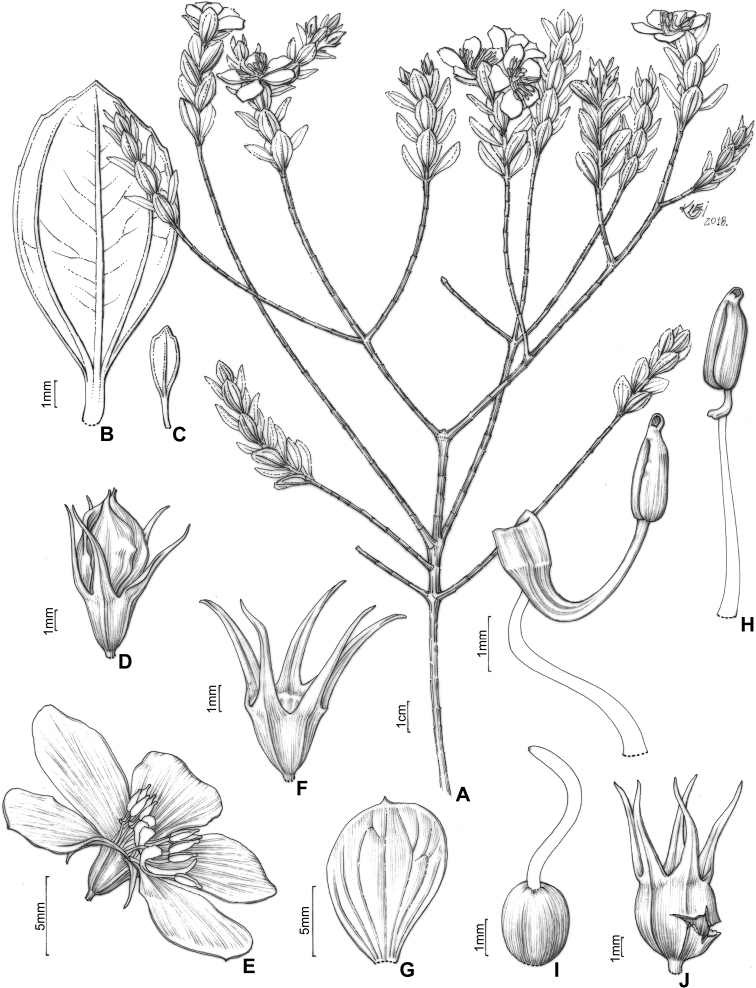
*Microliciapentagona***A** habit **B** leaf abaxial surface **C** bracteole abaxial surface **D** floral bud **E** flower in lateral view **F** flowering hypanthium **G** petal adaxial surface **H** antesepalous (left) and antepetalous (right) stamens **I** gynoecium **J** capsule enveloped by the hypanthium. Drawn from Sazima CFSC4259 (UEC) and Almeda et al. 9203 (UEC).

#### Distribution, habitat and elevation range.

Endemic to Minas Gerais State (Fig. 25B) in Serra do Caraça, Serra do Cipó, Serra de Ouro Preto, Serra de Ouro Branco, Serra do Itabirito, Mariana and Barão de Cocais. It occurs on quartzitic and ferruginous campo rupestre, exposed to full sun, at elevations of about 1000–1830 m.

#### Conservation.

*Microliciapentagona* is known from about 70 collections. The EOO is 2,667.623 km^2^ and the AOO is 56 km^2^. Several populations are protected within the following conservation units: Parque Estadual do Itacolomi, Parque Estadual Serra do Intendente, Parque Estadual Serra do Ouro Branco, Parque Nacional da Serra do Cipó and RPPN Serra do Caraça (Natural Heritage Private Reserve). Following the IUCN (2019) recommendations and criteria, we recommend an Endangered (EN): B1ab(iii) status for this species.

#### Recognition and affinities.

*Microliciapentagona* can be recognised by its branches, leaves and hypanthia that are glandular-punctate and vernicose, leaves 5-nerved from the base with one pair of acrodromous veins and one pair of tenuous veins close to the margin, inflorescences reduced to solitary terminal flowers and calyx lobes 6.2–8.5 mm long that become thickened in fruit. The elongate calyx lobes of *M.pentagona* are comparable in length only to those of *M.laniflora* (7.9–9.7 mm long) and are consistently longer than those of all remaining congeners. In fruit, the thickened calyx lobes of *M.pentagona* are unique in the clade. Overall, *M.pentagona* is morphologically similar to *M.calycina*, *M.chamissoana*, *M.laniflora* and *M.parviflora*. For comparisons, see comments under these species.

#### Notes.

*Microliciapentagona* is remarkably variable in leaf shape and size. The leaf blades vary from almost linear (e.g. *Irwin et al. 20537*, *Barreto 10734*) to narrowly elliptic (e.g. *Barreto 7025*, *Joly et al. CFSC 3196*) and elliptic (e.g. *Silva 1043*). Examples of variation in leaf size are *Almeda et al. 9203* (1.7–2.1 cm long) and *Silva 1043* (2.3–3.1 cm long). Some modal extremes were informally recognised as distinct taxa by Mello Barreto on herbaria labels. As no character varies in a meaningful way, we agree with Martins (1997) and treat all these extremes within the variation here attributed to *M.pentagona*.

#### Specimens examined.

**Brazil. Minas Gerais**: Barão de Cocais Municipality, Brandão 20838 (HUFU); Catas Altas Municipality, Serra do Caraça, Hatschbach et al. s.n. (HUFU [25601]), Irwin et al. 29213 (NY), Oliveira et al. 478 (BHCB), Oliveira et al. 480 (BHCB, RB), Oliveira et al. 518 (BHCB), Silva et al. 1043 (HUFU), Sobral et al. 14508 (HUFSJ, RB); Santa Bárbara Municipality [“Capanema”], Claussen 296 (W), Vainio 33501 (US); Jaboticatubas Municipality [“Caeté”], Serra do Cipó, Magalhães 2278 (BHCB, US); Mariana Municipality, Pivari et al. 2512 (BHCB); Ouro Branco Municipality, Saint-Hilaire s.n. (lectotype: P [P00723399]; isolectotype: P [P00723398]); Ouro Preto Municipality, Antônio-Silva et al. 315 (HUFU, OUPR), Bortoluzzi et al. 678 (RB, VIC), Damazio s.n. (RB [48352], NY [NY00942037]), Michelangeli et al. 1580 (NY, UPCB), Pacifico & Bressan 294 (HUEM, SPF), Peron 256 (RB), Peron 260 (RB), Rezende 507 (HUFU), Rolim et al. 353 (UPCB, VIC), Rolim et al. 361 (NY, RB, VIC), Rolim et al. 370 (HUFU, NY, RB, VIC), Schwacke 10814 (W), Schwacke 9325 (RB, W); Santa Bárbara Municipality, Serra do Caraça, Almeda et al. 7751 (CAS, HUFU, UEC), Damazio s.n. (RB [48392]), Ordones et al. 213 (BHZB, HUFU), Pirani & Yano 692 (RB, SP, SPF), Rapini et al. 296 (HUEM, SP, SPF), Romero et al. 5303 (CAS, UEC); Santana do Riacho Municipality, Serra do Cipó, Almeda et al. 8555 (CAS, UEC), Almeda et al. 8911 (CAS, UEC), Almeda et al. 9203 (CAS, HUEM, UEC), Barreto 1182 (RB), Contro & Marques 21 (HUEM, HUFU), Duarte 2004 (FLOR, MBM, RB), Fernandes s.n. (HUFU [56535]), Irwin et al. 20178 (CAS, MO, NY, UEC, US), Irwin et al. 20486 (CAS, MO, NY, US), Irwin et al. 20537 (CAS, NY, UEC, US), Pacifico & Bressan 531 (CAS, HUEM, RB). Romero & Nakajima 5998 (HUEM, HUFU, UEC), Semir CFSC5608 (SP, SPF), Semir & Sazima CFSC3395 (UEC); Santana do Riacho Municipality [“Conceição do Mato Dentro”], Serra do Cipó, Barreto 7025 (BHCB, HB, UEC); Santana do Riacho Municipality [“Jaboticatubas”], Serra do Cipó, Giulietti & Menezes 4017 (SP, SPF, UEC), Hatschbach et al. 28759 (MBM, SPF, US), Hatschbach et al. 29958 (MBM), Joly & Semir CFSC2957 (UEC), Joly & Semir CFSC3196 (SPF, UEC), Sazima CFSC4259 (RB, SP, UEC), Semir & Joly CFSC3743 (UEC); Santana do Riacho Municipality [“Santa Luzia”], Serra do Cipó, Barreto 10734 (HB, UEC), Barreto 7023 (BHCB), Barreto 7024 (UEC), Brade 14756 (R), Duarte 2692 (R, RB, US); Unknown municipality in Minas Gerais State, Casaretto s.n. (G [G00318565]), Claussen 1617 (P), Claussen 1637 (P), Claussen 22 (P, US), Claussen 350 (W), Claussen s.n. (P [P005317034], P [P005317035]), Glaziou 14742 (K, NY, P, R), Ule 2543 (US), Schüch s.n. (W [70510]).

### ﻿8. Microlicia pithyoides

Taxon classificationPlantaeMyrtalesMelastomataceae

(Cham.) Versiane & R.Romero, Bot. J. Linn. Soc. 197: 54. 2021.

E496D051-C3ED-5E9D-893D-D00C3FC7ADE7

Fig. 27

Trembleya pithyoides Cham., Linnaea 9(4): 428. 1835. **basionym**. Type: Brazil. “Caraça” [Minas Gerais, Serra do Caraça], 20 December 1830, *F. Sellow 1316* (lectotype, designated here: K [K00530665]!; isolectotypes: F-BN016638-photo]!, P [P00723396]!, P [P00723397]!; image of isolectotype at P is available at http://coldb.mnhn.fr/catalognumber/mnhn/p/p00723396).
Trembleya pithyoides var. major Cogn. in Martius et al., Fl. Bras. 14(4): 594. 1888. **syn. nov.** Type: Brazil. “Minas, Serra de Capanema” [Minas Gerais, Santa Bárbara], 21 February 1884, *A.F.M. Glaziou 14746* (lectotype, designated here: P [P00723395]!; isolectotypes: BR [BR0000005223930]!, BR [BR0000005520169]!, C [C10015114-online image]!, C [C10015113-online image]!, K [K00530666]!, P [P00723394]!, P [P00723393]!; image of lectotype is available at http://coldb.mnhn.fr/catalognumber/mnhn/p/p00723395).

#### Description.

Erect, densely-branched shrubs 0.3–0.6 m tall. Branchlets quadrangular, glandular-punctate, vinaceous (when fresh). Internodes 0.2–0.5 cm long, angles with narrow wings ca. 0.2 mm wide. Petioles 0.7–1.5 mm long. Leaf blades 4–15 mm long, 0.5–1.4 mm wide, chartaceous (when dry), linear, both surfaces green (when fresh), adaxial surface blackened and abaxial surface pale green to pale brown (when dry), discoloured (when dry), base cuneate, apex rounded to acute, margin flat, entire and glandular-punctate, 1-nerved from the base, tertiaries not evident on the abaxial surface, adaxial surface glandular-punctate, abaxial surface glandular-punctate. Inflorescences reduced to solitary flowers apically on the branches. Bracts absent. Bracteoles (at anthesis) with petioles 0.6–0.9 mm long, blades 3.0–3.9 mm long, 0.3–0.5 mm wide, lanceolate, base cuneate, apex acute to obtuse, margin entire, 1-nerved, indumentum like that of the principal leaves. Flowers 5-merous, pedicels (at anthesis) 0.3–0.6 mm long. Hypanthia (at anthesis) 1.7–2.0 mm long, 1.9–2.1 mm wide at the torus, campanulate, light green or reddish (when fresh), externally glandular-punctate. Calyx tubes 0.3–0.4 mm long. Calyx lobes (at anthesis) 2.5–3.0 mm long, 0.4–0.7 mm wide at the base, subulate, apex acute, margin entire, (when fresh) light green or reddish externally like the hypanthia. Petals 5.0–5.7 mm long, 3.7–4.2 mm wide, magenta, obovate, apex acuminate, margin entire and glabrous, adaxial surface glabrous or sparsely glandular-punctate, abaxial surface glabrous. Stamens 10, strongly dimorphic. Larger (antesepalous) stamens 5, filaments 2.6–2.9 mm long, pink, pedoconnectives 2.9–3.2 mm long, pink, appendages 0.7–0.9 mm long, yellow, apex emarginate to bilobate, thecae (excluding rostra) 1.8–2.0 mm long, vinaceous, oblong, rostra 0.2–0.4 mm long, the circular pores ca. 0.2 mm wide. Smaller (antepetalous) stamens 5, filaments 1.9–2.1 mm long, pink, pedoconnectives 0.6–0.9 mm long, yellow, inconspicuous appendages ca. 0.1 mm long, yellow, apex emarginate, thecae (excluding rostra) 1.4–1.6 mm long, yellow, oblong, rostra 0.2–0.3 mm long, the circular pores ca. 0.2 mm wide. Ovary 1.3–1.5 mm long, 1.0–1.2 mm wide, globose, (4–)5-locular. Style 4.0–4.5 mm long, pink. Capsules (at maturity) 2.7–3.1 mm long, 2.9–3.3 mm wide, globose, initially enveloped by the hypanthium, torus constricted at the apex, fruiting calyx tubes 0.4–0.6 mm long, fruiting calyx lobes 3.2–3.7 mm long, not thickened. Seeds 0.7–0.8 mm long, reniform.

**Figure 27.**
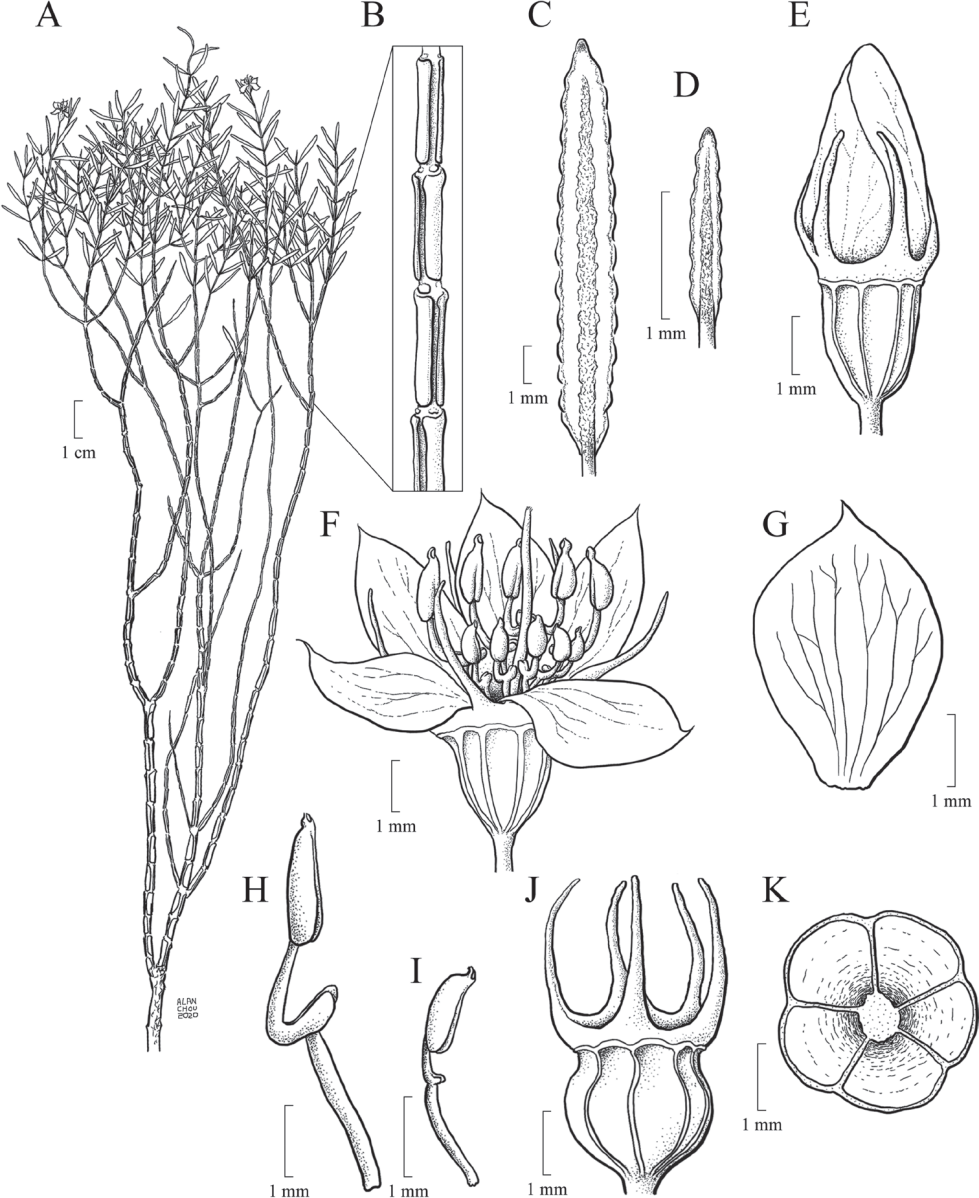
*Microliciapithyoides***A** habit **B** detail of nodes and internodes **C** leaf abaxial surface **D** bracteole abaxial surface **E** floral bud **F** flower in lateral view **G** petal adaxial surface **H** antesepalous stamen **I** antepetalous stamen **J** capsule enveloped by the hypanthium **K** capsule in cross section. Drawn from Pacifico 295 (CAS).

#### Specimens examined.

**Brazil. Minas Gerais**: Catas Altas Municipality, Serra do Caraça, Oliveira 382 (BHCB, RB), Pacifico 295 (CAS, HUEM, SPF); Unknown municipality in Minas Gerais State, Serra do Caraça. Glaziou 14746 (BR, C, K, P, R), Glaziou 19239 (K, P), Sellow 1316 (lectotype: K [K00530665; isolectotypes: F-BN016638-photo, P [P00723396], P [P00723397]), Sellow s.n. (K [K00957781, K [K00957783]), Weddell s.n. (P [P005317975]).

#### Distribution, habitat and elevation range.

Known only from Minas Gerais State (Fig. 25B), where it is probably endemic to the Serra do Caraça. *Microliciapithyoides* grows on quartzitic campo rupestre exposed to full sun at elevations between 1827 and 2072 m. It is the only species in the clade that reaches the highest peak in Cadeia do Espinhaço, the Pico do Sol in Catas Altas Municipality (elev. 2,072 m) (personal observation by R. Pacifico).

#### Conservation.

*Microliciapithyoides* species is the least collected species of the *Trembleya* s.s. clade and apparently has the narrowest distribution. Less than 10 collections of this species are housed in herbaria. It had not been collected for more than a hundred years until it was re-discovered in 2009 (*Oliveira 382*). As all coordinates available for *M.pithyoides* refer to the same population, we were unable to calculate its EOO. The AOO is 4 km^2^. The only population known of this species occurs inside a private protected area, the RPPN Serra do Caraça (Natural Heritage Private Reserve). The type material was probably collected at the Serra de Capanema in Santa Bárbara Municipality. Currently, this locality is also part of a private property. We are not aware of recent collections of *M.pithyoides* from the Serra de Capanema. Overall, the vegetation of Serra do Caraça is largely intact and affords a good measure of protection for the populations of *M.pithyoides*. This species is considered Critically Endangered by the Brazilian Government (Brasília 2014). Based on criterion B of the IUCN (2019), we concur with this assessment: (CR): B1ab(iii).

#### Recognition and affinities.

*Microliciapithyoides* can be recognised by its narrow leaves that are 0.5–1.4 mm wide, 1-nerved from the base, inflorescences reduced to solitary flowers, magenta petals and stamens with bicoloured anthers. In morphology, it is most like *M.rosmarinoides*, the only congener with leaves that are 1-nerved from the base. *Microliciarosmarinoides* also shares with *M.pithyoides* the solitary flowers with subulate calyx lobes and the overall shape of its stamens. *Microliciapithyoides* differs from *M.rosmarinoides* by the leaves with the mid-vein thickened (vs. not thickened), magenta petals (vs. yellow) and stamens with anthers vinaceous and yellow (vs. all anthers yellow to orange). Both species occur in central Minas Gerais State, but their distributions do not overlap; *M.rosmarinoides* has never been collected on the Serra do Caraça.

*Microliciacalycina* is also morphologically similar. It shares with *M.pithyoides* the narrow leaves, solitary flowers (sometimes simple dichasia only in *M.calycina*), magenta petals and stamens with bicoloured anthers. *Microliciapithyoides* may be differentiated by its leaf blades (oblong to lanceolate) 0.5–1.4 mm wide (vs. 2–9 mm wide), 1-nerved from the base (vs. 3-nerved) with tertiaries not evident (vs. evident) and shorter calyx lobes 2.5–3.0 mm long (vs. 3.5–4.2 mm long). Both *M.calycina* and *M.pithyoides* occur sympatrically on the Serra do Caraça, but only *M.pithyoides* occurs on the highest peak in that mountain range. In fact, *M.pithyoides* apparently prefers slightly higher elevations since it has only been collected between 1827 and 2072 m (vs. 1692–1920 m for *M.calycina*).

#### Notes.

Recent collections of *M.pithyoides* (*Oliveira 382*, *Pacifico 295*) have leaves with blades that are 8–12 mm long. These measurements bridge those given in the protologues of varieties *pithyoides* (8–10 mm long) and *major* (12–20 mm long). We consider these size differences to represent a continuum and here relegate var.major to synonymy.

### ﻿9. Microlicia rosmarinoides

Taxon classificationPlantaeMyrtalesMelastomataceae

(Mart. & Schrank ex DC.) Versiane & R.Romero, Bot. J. Linn. Soc. 197: 54. 2021.

B8F04F4B-99D2-5765-9869-CAEDD9BA3A78

Fig. 28

Trembleya rosmarinoides Mart. & Schrank ex DC., Prodr. 3: 125. 1828. **basionym**. Type: Brazil. ”Habitat in summo Monte de V. Rica et in Itacolumi 5000 ped. alt. Provinciae Min. Gen.” [Minas Gerais, Ouro Preto], *C.F.P. Martius 808* (lectotype, designated here: M [M0165886]!; isolectotype: G [G00310213-online image]!).

#### Description.

Erect, densely branched shrubs 0.3–0.6 m tall. Branchlets quadrangular, glandular-punctate, light green (when fresh). Internodes 0.1–0.4 cm long, angles with narrow wings 0.2–0.3 mm wide. Petioles 0.5–1.2 mm long. Leaf blades 4–10 mm long, 1.0–1.9 mm wide, chartaceous (when dry), narrowly-lanceolate to narrowly-elliptic, both surfaces green (when fresh), adaxial surface blackened and abaxial surface pale green to pale brown (when dry), discoloured (when dry), base cuneate, apex rounded to acute, margin flat, entire and glandular-punctate, 1-nerved from the base, tertiaries little evident on the abaxial surface, adaxial surface glandular-punctate, abaxial surface glandular-punctate. Inflorescences reduced to solitary flowers on apical branches. Bracts absent. Bracteoles (at anthesis) with petioles 0.5–1.0 mm long, blades 2.5–3.3 mm long, 0.5–0.9 mm wide, lanceolate, base cuneate, apex acute to obtuse, margin entire, 1-nerved, indumentum like that of the principal leaves. Flowers 5-merous, pedicels (at anthesis) 0.4–0.6 mm long. Hypanthia (at anthesis) 2.2–3.5 mm long, 1.5–2.0 mm wide at the torus, campanulate to urceolate, light green (when fresh), externally glandular-punctate. Calyx tubes 0.3–0.5 mm long. Calyx lobes (at anthesis) 2.2–2.8 mm long, 0.5–0.8 mm wide at the base, subulate, apex acute, margin entire, (when fresh) light green, externally like the hypanthia. Petals 5.0–5.3 mm long, 2.4–3.0 mm wide, yellow, obovate, apex acute or rounded, margin entire and glabrous, both surfaces glabrous. Stamens 10, strongly dimorphic. Larger (antesepalous) stamens 5, filaments 2.2–3.0 mm long, yellow, pedoconnectives 3.2–3.8 mm long, yellow, appendages 0.8–1.0 mm long, yellow, apex emarginate to bilobate, thecae (excluding rostra) 1.4–1.7 mm long, yellow to orange, oblong, rostra 0.2–0.3 mm long, the circular pores ca. 0.2 mm wide. Smaller (antepetalous) stamens 5, filaments 1.5–2.0 mm long, yellow, pedoconnectives 0.8–1.0 mm long, yellow, inconspicuous appendages ca. 0.1 mm long, yellow, apex emarginate, thecae (excluding rostra) 1.4–1.6 mm long, yellow to orange, oblong, rostra 0.2–0.3 mm long, the circular pores ca. 0.2 mm wide. Ovary 0.9–1.1 mm long, 0.7–0.9 mm wide, ovoid, 5-locular. Style ca. 3 mm long, yellow. Capsules (at maturity) 2.5–3.5 mm long, 2.0–3.0 mm wide, globose, initially enveloped by the hypanthium, torus constricted at the apex, fruiting calyx tubes 0.3–0.6 mm long, fruiting calyx lobes 2.5–3.2 mm long, not thickened. Seeds 0.5–0.9 mm long, reniform.

**Figure 28.**
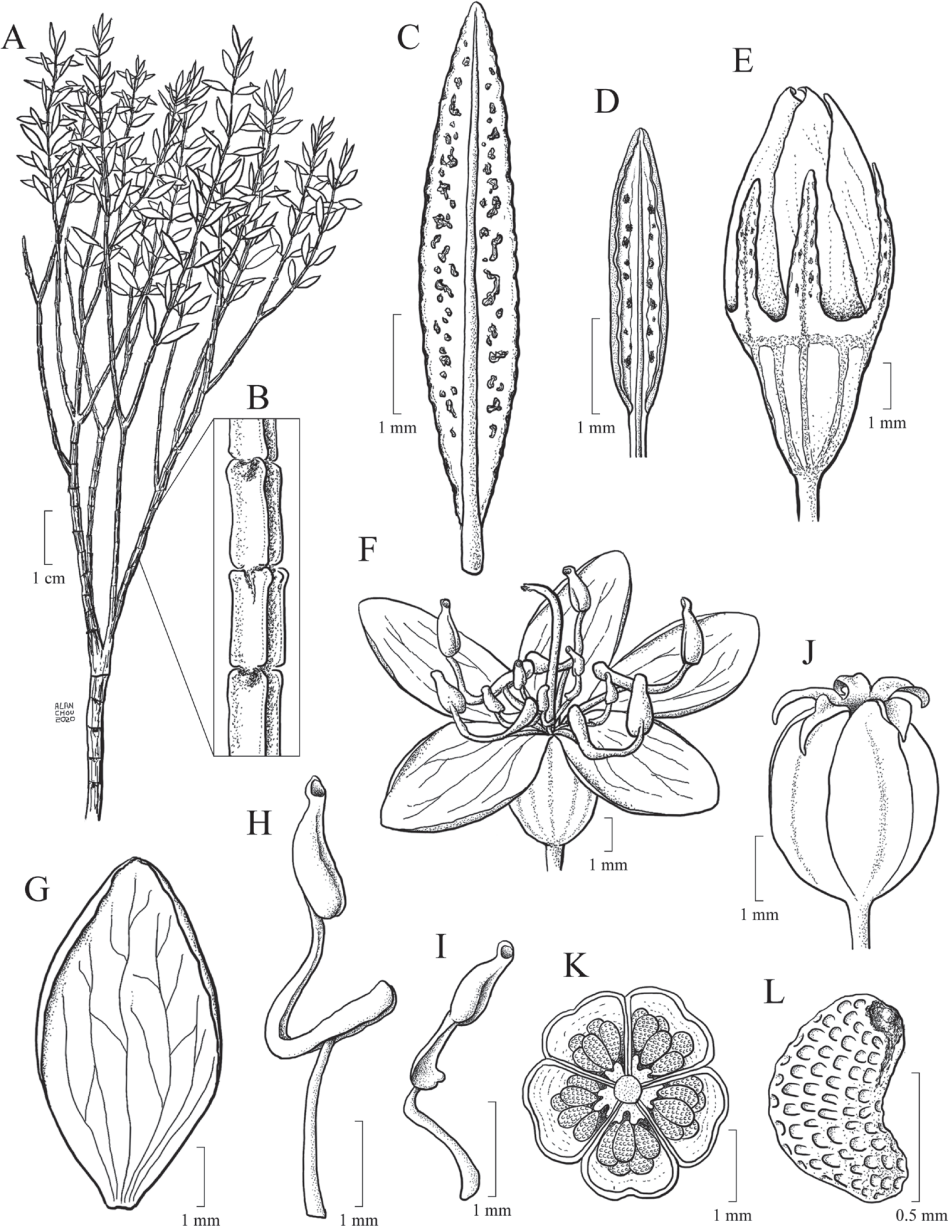
*Microliciarosmarinoides***A** habit **B** detail of nodes and internodes **C** leaf abaxial surface **D** bracteole abaxial surface **E** floral bud **F** flower in lateral view **G** petal adaxial surface **H** antesepalous stamen **I** antepetalous stamen **J** capsule enveloped by the hypanthium **K** capsule in cross section **L** seed in lateral view. Drawn from Occhioni et al. s.n. (US [US001900109]).

#### Distribution, habitat, and elevation range.

Restricted to central Minas Gerais State (Fig. 25B), where it is known only from Serra do Gandarela, Serra de Capanema, Serra de Ouro Preto, Serra de Itabirito, Belo Vale and Barão de Cocais. *Microliciarosmarinoides* occurs in campo rupestre and canga, on ferruginous or quartzitic soils, exposed to full sun, at elevations between 1609 and 1807 m.

#### Conservation.

*Microliciarosmarinoides* is a little-collected species, known from about 15 specimens. The EOO is 298.172 km^2^ and the AOO is 28 km^2^. Populations of *M.rosmarinoides* are protected in the Parque Nacional da Serra do Gandarela and probably in Parque Estadual do Itacolomi. Following IUCN (2019) criteria, we recommend an assessment of Endangered (EN): B1ab(iii) for this species.

#### Recognition and affinities.

*Microliciarosmarinoides* can be readily recognised amongst congeners by its narrow leaves (1–1.9 mm wide) and flowers with yellow petals. The narrow leaves of *M.rosmarinoides* are more similar to those of the closely related *M.pithyoides*, while the yellow petals are shared only with the distantly related *M.flaviflora*. For comparisons, see notes under *M.flaviflora* and *M.pithyoides*.

#### Specimens examined.

**Brazil.** Minas Gerais: Barão de Cocais, Mina do Baú, Souza et al. 2584 (BHCB); Belo Vale, Occhioni et al. s.n. (RFA, US [US001900109]); Itabirito, Serra de Capanema, Carmo 192 (BHCB), Carmo 377 (BHCB); Ouro Preto municipality, Serra do Itacolomi, Martius 808 (lectotype: M [M0165886]; isolectotype: G [G00310213]), Pedrosa 110 (HUFU, OUPR), Schwacke 9184 (BHCB, RB, US, W); Rio Acima Municipality, Serra do Gandarela, Carmo 2262 (BHCB), Versiane & Castello 677 (HUFU, UEC), Vidal s.n. (BHCB [191426]); Santa Bárbara Municipality, Serra do Gandarela, Vidal s.n. (BHCB [181346]); Unknown municipality in Minas Gerais State, Glaziou 14747 (IAN, K, RB, US), Glaziou 19242 (K), Ule 2528 (US).

### ﻿10. Microlicia trembleyiformis

Taxon classificationPlantaeMyrtalesMelastomataceae

Naudin, Ann. Sci. Nat., Bot. Sér. 3, 3: 172. 1845.

B63009A7-DA81-5549-9E75-68CE994BB394

Fig. 29

#### Type.

Brazil. “Minas Geraes, in campis circa urbem Villa Ricca frequens” [Minas Gerais, Ouro Preto], 1816–1821, [catal. B1, n° 160] *A. Saint-Hilaire s.n.* (lectotype, first-step designated by Martin and Cremers (2007), second-step designated here: P [P002297746]!; isolectotype: F [F0360366]!; image of lectotype is available at http://coldb.mnhn.fr/catalognumber/mnhn/p/p02297746).

#### Description.

Erect shrubs 0.5–1.0 m tall. Branchlets quadrangular, glandular-punctate and sparsely covered with eglandular trichomes 0.1–0.5, light green (when fresh). Internodes 0.2–1.1 cm long, angles with narrow wings ca. 0.2 mm wide. Petioles 0.3–1.4 mm long. Leaf blades 4–25 mm long, 1.5–12 mm wide, papyraceous (when dry), ovate or elliptic, both surfaces green (when fresh), adaxial surface blackened and abaxial surface pale green to pale brown (when dry), discoloured (when dry), base rounded or attenuate, apex acute or obtuse, margin flat, slighly serrulate and ciliate with eglandular trichomes 0.1–0.4 mm long, 5-nerved from the base, one pair of acrodromous veins and one pair of tenuous veins close to the margin, tertiaries evident on the abaxial surface, nearly perpendicular to the mid-vein, little reticulate and branching apically, adaxial surface glandular-punctate, abaxial surface densely glandular-punctate and sparsely covered with eglandular trichomes 0.1–0.5 mm long around the veins. Inflorescences reduced to solitary flowers on the aplical region of the branches. Bracts absent. Bracteoles (at anthesis) with petioles 0.3–0.5 mm long, blades 2.4–6.1 mm long, 1.0–3.2 mm wide, ovate or elliptic, base cuneate, apex acute to obtuse, margin slightly serrulate and ciliate with eglandular trichomes 0.1–0.4 mm long, 3-nerved, indumentum like that of the principal leaves. Flowers 5-merous, pedicels (at anthesis) 0.7–1.9 mm long. Hypanthia (at anthesis) 2.2–2.6 mm long, 1.8–2.2 mm wide at the torus, campanulate to urceolate, light green (when fresh), externally glandular-punctate and sparsely covered with eglandular trichomes 0.1–0.5. Calyx tubes 0.2–0.4 mm long. Calyx lobes (at anthesis) 1.5–2.1 mm long, 0.5–0.7 mm wide at the base, narrowly triangular, apex acute, margin entire, (when fresh) light green, externally like the hypanthia. Petals 6.1–7.9 mm long, 3.0–3.5 mm wide, magenta, obovate, apex acute, margin entire and glabrous, both surfaces glabrous. Stamens 10, strongly dimorphic. Larger (antesepalous) stamens 5, filaments 1.5–3.0 mm long, pink, pedoconnectives 1.9–2.9 mm long, pink, appendages 1.0–1.5 mm long, yellow, apex bilobate, thecae (excluding rostra) 1.0–1.5 mm long, purple, oblong, rostra 0.2–0.3 mm long, the circular pores ca. 0.2 mm wide. Smaller (antepetalous) stamens 5, filaments 1.5–2.0 mm long, pink, pedoconnectives 0.8–1.2 mm long, yellow, inconspicuous appendages ca. 0.1 mm long, yellow, apex emarginate, thecae (excluding rostra) 0.7–1.1 mm long, yellow, oblong, rostra 0.2–0.3 mm long, the circular pores ca. 0.2 mm wide. Ovary 1.8–2.0 mm long, 1.9–2.1 mm wide, globose, 3–5-locular. Style 3.7–4.2 mm long, pink. Capsules (at maturity) 2.5–3.4 mm long, 2.4–3.0 mm wide, globose, initially enveloped by the hypanthium, torus constricted at the apex, fruiting calyx tubes 0.3–0.4 mm long, fruiting calyx lobes 2.4–2.9 mm long, not thickened. Seeds not seen.

**Figure 29.**
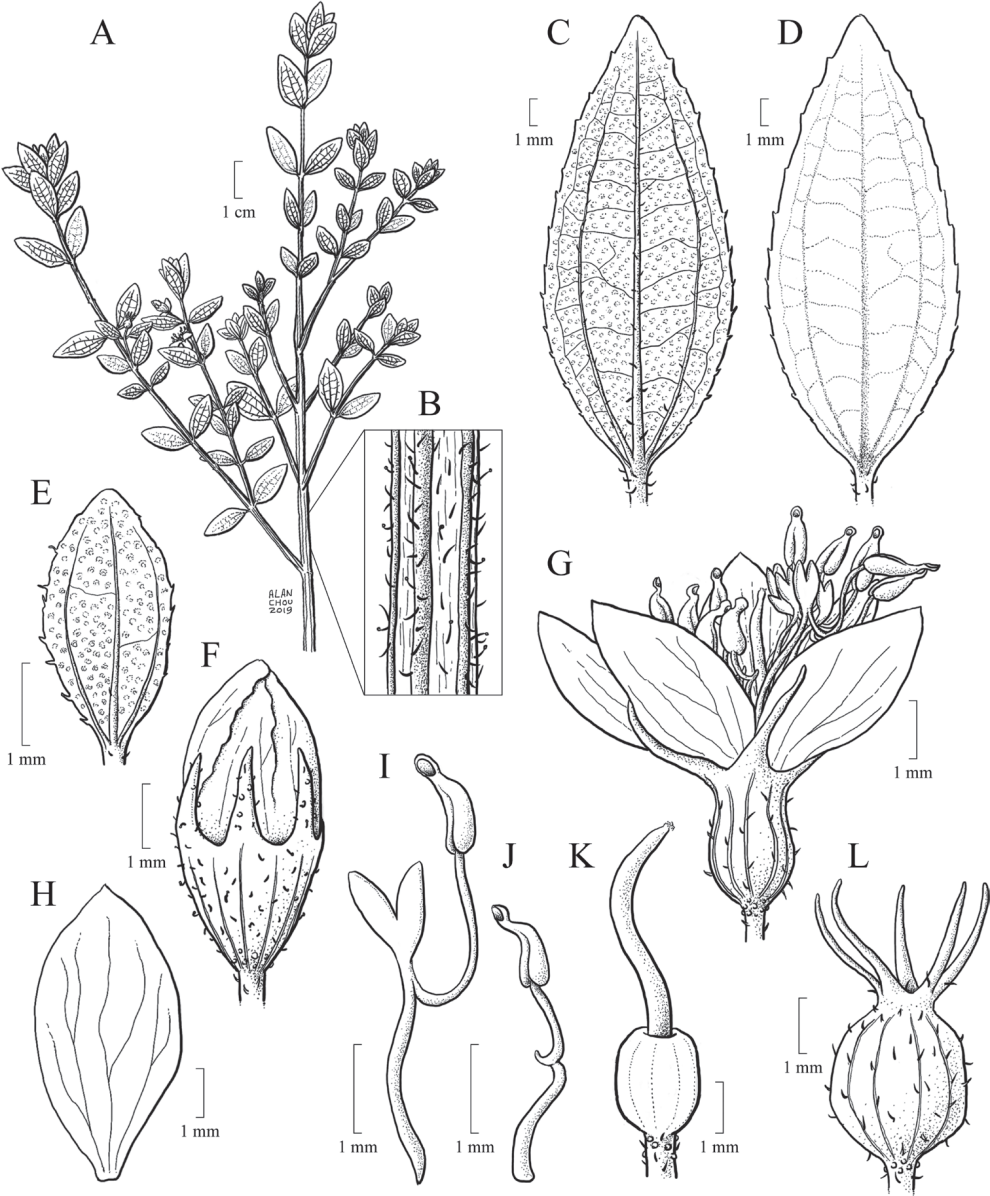
*Microliciatrembleyiformis***A** habit **B** detail of nodes and internodes **C** leaf abaxial surface **D** leaf adaxial surface **E** bracteole abaxial surface **F** floral bud **G** flower in lateral view **H** petal adaxial surface **I** antesepalous stamen **J** antepetalous stamen **K** gynoecium **L** capsule enveloped by the hypanthium. Drawn from Duarte 2767 (US).

#### Distribution, habitat and elevation range.

*Microliciatrembleyiformis* is known from quartzitic campo rupestre, Cerrado and veredas (palm swamps) at Ouro Preto, Serra da Canastra, Uberlândia and Patrocínio (in Minas Gerais State) and campos de altitude in Serra Negra (Itatiaia), Rio de Janeiro State (Fig. 19B). On Serra da Canastra, it was collected on sandy soils near streams, exposed to full sun, at elevations between 786 and 1300 m. Collections from Ouro Preto and Serra Negra lack additional habitat information.

#### Conservation.

This species is known from about 10 specimens. It is a little-collected species that, however, has a comparatively wide distributional range. The EOO is 92,214.048 km^2^, a value that would indicate a Least Concern conservation status if criterion B of IUCN (2019) was applied. However, the AOO of 24 km^2^ matches a status of Endangered in accordance with the same criterion. Both EOO and AOO requirements have to be fulfilled for the correct use of criterion B. Thus, we recommend a status of Least Concern (LC) for *M.trembleyiformis*. We suspect that this species occurs in low population densities. Some populations of *M.trembleyiformis* are protected in the Parque Nacional da Serra da Canastra.

#### Recognition and affinities.

*Microliciatrembleyiformis* can be recognised by its elliptic to ovate leaves that are 5-nerved from the base and solitary flowers with narrowly-triangular calyx lobes 1.5–2.1 mm long. In morphology, it is closest to *M.parviflora* and more distantly to *M.altoparaisensis* (see notes under these species for comparisons). *Microliciapentagona* is the only congener that shares with *M.trembleyiformis* both the leaves 5-nerved from the base and solitary flowers. *Microliciatrembleyiformis* differs in having leaves that are papyraceous when dry (vs. chartaceous to coriaceous in *M.pentagona*), the margin is ciliate with eglandular trichomes 0.1–0.4 mm long (vs. glabrous to minutely granulose) and the shorter calyx lobes are 1.5–2.1 mm long (vs. 6.2–8.5 mm long) and tenuous in fruit (vs. thickened). Amongst the compared species, only *M.parviflora* occurs in Ouro Preto, Serra da Canastra and Serra Negra, where sympatry with *M.trembleyiformis* is possible.

#### Notes.

According to Naudin (1845), this species was frequent in Ouro Preto Municipality, although we are not aware of any recent collections from that region. Naudin (1845) justified the placement of *M.trembleyiformis* in *Microlicia* because the type had 3-locular ovaries. Recent collections of *M.trembleyiformis* have 3–5-locular ovaries (e.g. *Porto 2834*). In the Parque Nacional da Serra da Canastra, *M.trembleyiformis* is known from only a few individuals that consistently have 3-locular ovaries (Romero 2000).

#### Specimens examined.

**Brazil. Minas Gerais**: Capitólio Municipality, estrada para Cachoeira Fecho da Serra, Romero et al. 7550 (HUFU, US); Ouro Preto Municipality, [catal. B1, n° 160] *Saint-Hilaire s.n.* (lectotype: P [P002297746]!; isolectotype: F [F0360366]); São Roque de Minas Municipality, Serra da Canastra, Nakajima et al. 1476 (HUFU, US), Romero & Nakajima 3593 (HUFU, K, UEC), Romero 4157 (BHCB, F, HUFU), Santos 411 (HUFU, K); Serra do Salitre Municipality, Serra de Catiara, Duarte 2767 (BHCB, US); Uberlândia Municipality, Clube Caça e Pesca Itororó, Romero et al. 8689 (HUFU), Romero et. al. 8694 (HUFU); unknown municipality in Minas Gerais State, Glaziou 19221 (K, P). **Rio de Janeiro**: Itatiaia Municipality, Serra Negra, Porto 2834 (NY, RB, US).

### ﻿11. Microlicia tridentata

Taxon classificationPlantaeMyrtalesMelastomataceae

(Naudin) Versiane & R.Romero, Bot. J. Linn. Soc. 197: 55. 2021.

87806F2F-6F9F-5A61-A181-469ADB02499A

Fig. 30

Trembleya tridentata Naudin, Ann. Sci. Nat., Bot. Sér. 3, 2: 154: 1844. **basionym**. Type: Brazil. “In montibus Serra de San Jose, provinciae Minas Geraes” [Minas Gerais, Serra de São José], 1816–1821, [catal. B2, n° 2397] *A. Saint-Hilaire s.n.* (lectotype, first-step designated by Martin and Cremers (2007), second-step designated here: P [P00723392]!; isolectotypes: P [P00723391]!, P [P00723506]!; image of lectotype is available at http://coldb.mnhn.fr/catalognumber/mnhn/p/p00723392).

#### Description.

Erect shrubs or treelets 1.0–1.8 m tall. Branchlets quadrangular, appearing glabrous, vernicose and minutely granulose, light green (when fresh). Internodes 0.3–1.5 cm long, angles with narrow wings 0.2–0.4 mm wide. Petioles 1.0–4.9 mm long. Leaf blades 20–49 mm long, 12–24 mm wide, coriaceous (when dry), elliptic, ovate, or rarely narrowly elliptic, both surfaces green (when fresh), adaxial surface blackened and abaxial surface pale green to pale brown (when dry), discoloured (when dry), base cuneate or attenuate, apex rounded to emarginate, margin flat, entire along the basal half, serrulate on the upper half and glandular-punctate, 5-nerved from the base, one pair of acrodromous veins and one pair of tenuous veins close to the margin, tertiaries evident on the abaxial surface, nearly perpendicular to the mid-vein, reticulate, randomly branching and surrounding stout depressions on the abaxial leaf surface, adaxial surface glandular-punctate, abaxial surface glandular-punctate. Inflorescences simple dichasia or reduced to solitary flowers, not congested. Bracts (including petioles) 1.0–1.6 cm long, 0.4–0.8 cm wide, 3- to inconspicuously 5-nerved, elliptic, indumentum like that of the principal leaves. Bracteoles (at anthesis) with petioles 2.5–5.0 mm long, blades 8.1–11.0 mm long, 4.0–5.5 mm wide, elliptic, base attenuate, apex rounded to obtuse, margin sparsely serrulate, 3-nerved, indumentum like that of the principal leaves. Flowers 5-merous, pedicels (at anthesis) 1.3–1.6 mm long. Hypanthia (at anthesis) 3.5–3.9 mm long, 2.3–2.7 mm wide at the torus, campanulate to urceolate, light green (when fresh), externally appearing glabrous, vernicose and minutely granulose. Calyx tubes 0.3–0.4 mm long. Calyx lobes (at anthesis) 4.1–4.9 mm long, 0.8–1.1 mm wide at the base, subulate, apex acute, margin entire, (when fresh) light green or reddish, externally like the hypanthia. Petals 11.5–13.0 mm long, 6.0–7.0 mm wide, magenta or rarely white, obovate, apex acute or rounded, margin entire and glabrous, both surfaces glabrous. Stamens 10, strongly dimorphic. Larger (antesepalous) stamens 5, filaments 4.9–5.6 mm long, pink or rarely white, pedoconnectives 6.0–6.5 mm long, pink or rarely white with the apical region flushed with pink, appendages 1.7–1.9 mm long, yellow, apex emarginate or truncate, thecae (excluding rostra) 1.7–1.9 mm long, purple, oblong, rostra 0.3–0.5 mm long, the circular pores ca. 0.2 mm wide. Smaller (antepetalous) stamens 5, filaments 4.0–4.4 mm long, pink or rarely white, pedoconnectives 1.5–1.9 mm long, pink or rarely white with the apical region flushed with pink, inconspicuous appendages ca. 0.2 mm long, yellow, apex emarginate or truncate, thecae (excluding rostra) 1.4–1.7 mm long, yellow, oblong, rostra 0.3–0.5 mm long, the circular pores ca. 0.2 mm wide. Ovary 2.2–2.4 mm long, 2.1–2.3 mm wide, globose, 5-locular. Style 6.1–6.5 mm long, pink or rarely white. Capsules (at maturity) 3.0–4.0 mm long, 2.7–3.6 mm wide, globose, initially enveloped by the hypanthium, torus constricted at the apex, fruiting calyx tubes 0.4–0.5 mm long, fruiting calyx lobes 4.5–5.4 mm long, not thickened. Seeds not seen.

**Figure 30.**
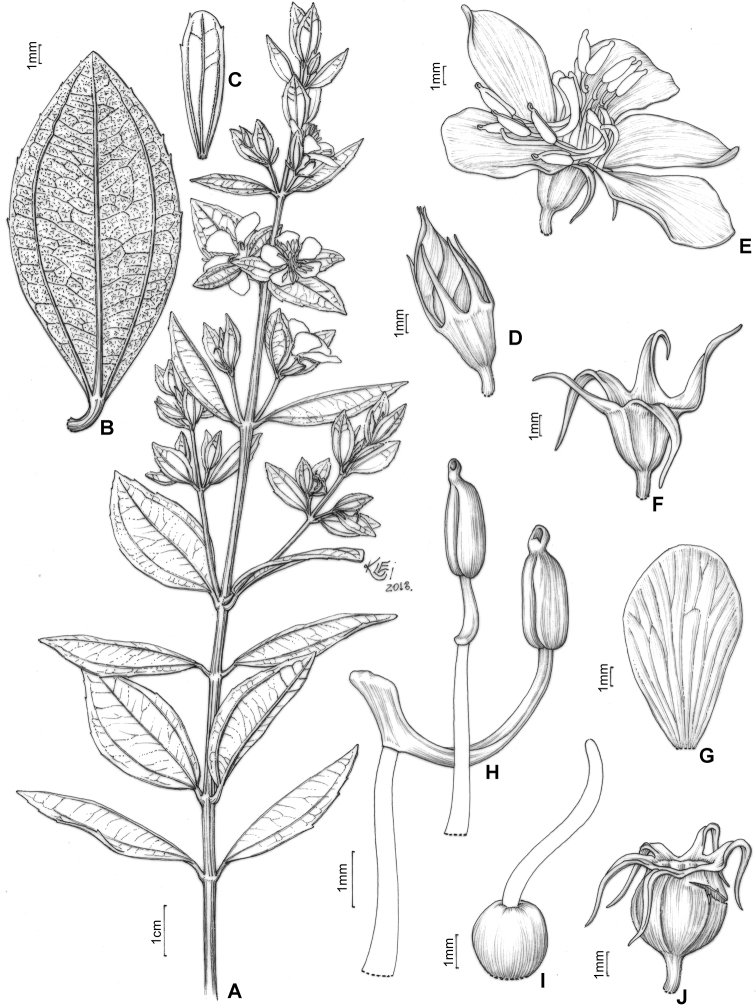
*Microliciatridentata***A** habit **B** leaf abaxial surface **C** bracteole abaxial surface **D** floral bud **E** flower in lateral view **F** flowering hypanthium **G** petal adaxial surface **H** antesepalous (behind) and antepetalous (in front) stamens **I** gynoecium **J** capsule enveloped by the hypanthium. Drawn from Hensold et al. CFCR2880 (UEC) and Souza et al. 8015 (UEC).

#### Distribution, habitat and elevation range.

*Microliciatridentata* is endemic to Minas Gerais State (Fig. 25A) at Serra de Ouro Branco, Serra de Ouro Preto, Serra de São José, Serra do Gandarela, Serra do Cipó, Serra do Lenheiro, Serra do Caraça and Barão de Cocais. It grows on quartzitic or ferruginous campo rupestre, exposed to full sun, at elevations between 1110 and 1614 m.

#### Conservation.

*Microliciatridentata* is known from about 40 collections. The EOO is 4,613.081 km^2^ and the AOO is 24 km^2^. Following IUCN (2019) recommendations, we recommend Endangered (EN): B1ab(iii) status for this species. Populations of *M.tridentata* are protected in Parque Estadual do Itacolomi, Parque Estadual Serra do Ouro Branco and RPPN Serra do Caraça (Natural Heritage Private Reserve).

#### Recognition and affinities.

*Microliciatridentata* can be recognised by its leaves that are serrulate along the upper half, 5-nerved from the base, abaxially conspicuously glandular-punctate, with tertiary veins reticulate and randomly branching and flowers with subulate calyx lobes 4.1–4.9 mm long. It shares with *M.pentagona* the shrubby to treelet habit and the serrulate leaves along the upper half that are 5-nerved from the base. *Microliciatridentata* is readily differentiated by the abaxial surfaces of the leaves that are more conspicuously glandular-punctate, with tertiares reticulate and randomly branching (vs. parallel or little reticulate and branching apically) and shorter calyx lobes 4.1–4.9 mm long (vs. 6.2–8.5 mm long) that are tenuous in fruit (vs. thickened). Another close relative is *M.parviflora*, from which *M.tridentata* differs by the leaves that are not pruinose (vs. usually pruinose), inflorescences composed of simple dichasia or reduced to solitary flowers (vs. compound or simple dichasia), bracteoles longer 8.1–11.0 mm long (vs. 2.2–3.8 mm long) and longer calyx lobes 4.1–4.9 mm long [vs. 0.7–2.5(–3.0)] that are subulate (vs. triangular). The distributions of *M.parviflora* and *M.pentagona* overlap with *M.tridentata* on several mountain ranges in Minas Gerais (e.g. Serra de Ouro Branco, Serra de Ouro Preto, Serra do Cipó).

#### Notes.

Major variation in *M.tridentata* involves inflorescence development (simple dichasia or solitary flowers) and the petals typically magenta (Fig. 8H), rarely white (Fig. 9H), which may be rounded or acute at the apex.

#### Specimens examined.

**Brazil. Minas Gerais**: Barão de Cocais Municipality, Irwin et al. 29029 (IAN, NY, US), Irwin et al. 29079 (K, MO, NY, P, RB); Catas Altas Municipality, Serra do Caraça, Castro et al. 281 (HUFU); Ouro Branco Municipality, Serra de Ouro Branco, Pacifico & Bressan 290 (CAS, HUEM), Souza et al. 8015 (ESA, SPF, UEC); Ouro Preto Municipality, Serra do Itacolomi, Badini & Ferreira 9774 (HUFU), Ferreira & Helena 7844 (ESA, HUFU), Giulietti et al. CFCR13780 (K, MO, SPF), Longhi-Wagner et al. CFCR9184 (SPF, UEC, US), Magalhães 1160 (IAN, US), Michelangeli et al. 1586 (NY, RB, UPCB), Michelangeli et al. 1595 (NY, UPCB), Pedrosa 120 (OUPR), Rolim et al. 326 (HUFU, VIC), Rolim et al. 327 (UPCB, VIC), Rolim et al. 328 (HUFU, VIC), Rolim et al. 394 (HUFU, NY, RB, VIC), Roschel et al. s.n. (OUPR [1506], RB [RB01301048]), Souza et al. 8047 (ESA, SORO, SPF), Souza et al. 8061 (ESA, RB, SPF, UPCB); Rio Acima Municipality, Serra do Gandarela, Pacifico & Bressan 303 (CAS, HUEM), Versiane & Devides 682 (UEC); Santa Bárbara Municipality, Serra do Caraça, Ordones et al. 84 (BHZB, HUFU), Pereira & Pabst 2619 (RB, US); Santana do Riacho Municipality, Serra do Cipó, Duarte 8157 (G, LE, RB, US); São João del-Rei Municipality, Schwacke 10135 (BHCB, RB, W), Glaziou 17513 (P, R, US), Serra do Lenheiro, Glaziou 16781 (K, NY, P, US); Tiradentes Municipality, Serra de São José, Alves 595 (US), Alves 7359 (R), Rutter 128 (R), Rutter 150 (R), Rutter 161 (R), Rutter 186 (R), Rutter 191 (R); Unknown municipality in Minas Gerais State, Claussen 1631 (P), Glaziou 14743 (K, P, US), Glaziou 17573 (K), Mendonça 557 (US), Riedel s.n. (K [K009597799, W-18800001419, W-18890019740]), *Saint-Hilaire s.n.* [catal. B2, n° 2397] (lectotype: P [P00723392]; isolectotypes: P [P00723391], P [P00723506]), Sellow s.n. (US [US00292635]); Serra da Conceição (Serra B. Vista), Hensold et al. CFCR2880 (SPF, US).

##### ﻿A nomenclatural review on the remaining taxa previously treated in *Trembleya*

**1. *Trembleyaacuminata* R.B.Pacifico & Fidanza, Phytotaxa 238(2): 164. 2015.** Type: Brazil. Minas Gerais, Joaquim Felício, Serra do Cabral, estrada Joaquim Felício–Várzea da Palma, ca. 24 km de Joaquim Felício, 10 July 2001, *V.C. Souza et al. 25654* (holotype: MBM!; isotypes: BHCB!, ESA!, SPF!).

**Replaced with**: *Microliciaacuminifolia* Versiane & R.Romero.

**2. *Trembleyaagrestis* Mart. & Schrank ex DC., Prodr. 3: 126. 1828.** Type: Brazil. “In prov. Minas Geraes” [Minas Gerais], *C.F.P. Martius s.n.* (holotype: M [M0165682]!, isotype [fragment]: BR [BR0000005521203]!).

**Basionym of**: *Microliciaagrestis* (Mart. & Schrank ex DC.) Cogn.

**3. *Trembleyabotaensis* R.B.Pacifico & Fidanza, Phytotaxa 238(2): 167. 2015.** Type: Brazil. Minas Gerais, Guaraciama, Cadeia do Espinhaço, Serra do Bota, 19 March 2005, *E. Guarçoni 903 & M. A. Sartori* (holotype: MBM!).

**Synonym of**: *Microliciacurralensis* Brade.

**4. *Trembleyaelegans* (Cogn.) Almeda & A.B.Martins, Novon 11(1): 6. 2001.** Type: Based on *Lavoisieraelegans* Cogn.

**Replaced with**: *Microliciaspeciosa* Versiane & R.Romero.

**5. *Trembleyainversa* Fidanza, A.B.Martins & Almeda, Brittonia 65(3): 281. 2013.** Type: Brazil. Minas Gerais, Joaquim Felício, Serra do Cabral, Armazém da Laje, 9 September 2003, *K. Fidanza & R. Belinello 12* (holotype: UEC!; isotype: CAS!).

**Basionym of**: *Microliciainversa* (Fidanza, A.B.Martins & Almeda) Versiane & R.Romero.

**6. *Trembleyaneopyrenaica* Naudin, Ann. Sci. Nat., Bot. Sér. 3, 2: 154. 1844.** Type: Brazil. “In montibus Pyreneos prov. Goyaz” [Goiás, Serra dos Pireneus], 1816, *A. Saint-Hilaire C1-694* (lectotype, first-step designated by Martin and Cremers (2007), second-step designated here: P [P00723416]!; isolectotypes: P [P00723417]!, P [P00723418]!, F [fragment] [F0064039F-online image]!; image of lectotype is available at http://coldb.mnhn.fr/catalognumber/mnhn/p/p00723416).

**Basionym of**: *Microlicianeopyrenaica* (Naudin) Versiane & R.Romero.

**7. *Trembleyaphlogiformis* Mart. & Schrank ex DC., Prodr. 3: 126. 1828.** Type: Brazil. “In Brasiliae campis prov. S.-Pauli” [São Paulo State], *Martius s.n.* (holotype: M [M0165885]!, probable isotype: G [G00310212]!).

**Basionym of**: *Microliciaphlogiformis* (Mart. & Schrank ex DC.) Versiane & R.Romero.

*Melastomapumilum* Vell., Fl. Flumin. 179. 1829. Type: Brazil. “Habitat campis apricis mediterraneis prope Predium Boavista inter gramina” (lectotype, designated here: Original illustration published in Vellozo, Fl. Flumin. Icones 3: t. 151. 1831; the original parchment is in the manuscript section of the Biblioteca Nacional, Rio de Janeiro; a copy of the illustration is deposited at G [G00368052]!).

Trembleyaphlogiformisvar.glabra Cham., Linnaea 9: 429. 1835. **syn. nov.** Type: Brazil. “In Brasilia australi loco haud indicato”, *Sellow 2387* (lectotype, designated here: S [S09-12942-online image]!; probable isolectotype: K [K000530643]!).

*Trembleyastachyoides* Naudin, Ann. Sci. Nat., Bot. Sér. 3, 2: 154. 1844. **syn. nov.** Type: Brazil. “In Brasilia australi, praecipue circa Tocoropa”, *Laruotte s.n.* (lectotype, first-step designated by Martin and Cremers (2007), second-step designated here: P [P00723388]!; isolectotype: P [P00723389]!; image of lectotype is available at http://coldb.mnhn.fr/catalognumber/mnhn/p/p00723388).

Trembleyaphlogiformisvar.cuneifolia Cogn. in Martius et al., Fl. Bras. 14(3): 132. 1883. **syn. nov.** Type: Brazil. “In prov. Minas Geraës ad Congonhas do Campo” [Minas Gerais], *Stephan s.n.* (lectotype, designated here: BR [BR0000005224203]!).

Trembleyaphlogiformisvar.genuina Cogn. in Martius et al., Fl. Bras. 14(3): 132. 1883. **syn. nov.** Type: Based on *Trembleyaphlogiformis* Mart. & Schrank ex DC.

Trembleyaphlogiformisvar.latifolia Cogn. in Martius et al., Fl. Bras. 14(3): 132. 1883. **syn. nov.** Type: Brazil. “In Brasilia loco haud indicato”, *Raben 781* (lectotype, designated here: BR [BR0000005520855]!; isolectotype: BR [BR0000005520190]!).

Trembleyaphlogiformisvar.microlicioides Cogn. in Martius et al., Fl. Bras. 14(4): 594. 1888. **syn. nov.** Type: Brazil. In prov. Rio de Janeiro, *Glaziou 16046* (lectotype, designated here: R [R000009186]!; isolectotypes: BR [BR0000005223596]!, BR [BR0000005520503]!, BR [BR0000005520176]!, C [C10015111-online image]!, G [G00368051]!, P [P00723400]!; image of isolectotype at P is available at http://coldb.mnhn.fr/catalognumber/mnhn/p/p00723400).

Trembleyaphlogiformisvar.parvifolia Cogn. in Martius et al., Fl. Bras. 14(3): 132. 1883. **syn. nov.** Type: Brazil. “In paludibus prope Lorena prov. S. Paulo”, *Riedel 1418* (lectotype, designated here: P [P05317991]!; isolectotypes: K [K000530649]!, P [P05317995]!; image of lectotype is available at http://coldb.mnhn.fr/catalognumber/mnhn/p/p05317991).

Trembleyaphlogiformisvar.quinquenervia Cogn. in Martius et al., Fl. Bras. 14(3): 132. 1883. **syn. nov.** Type: Brazil. “In prov. Goyaz” [Goiás], *Gardner 4147* (lectotype, designated here: BM [BM000516951]!; isolectotypes P [P05317981]!, P [P05317982]!; image of lectotype is available at http://plants.jstor.org and http://data.nhm.ac.uk/object/255f17a9-b371-4dd3-8eb0-9f0eafbd2e76).

Trembleyaphlogiformisvar.ramosissima Cogn. in Martius et al., Fl. Bras. 14(3): 132. 1883. **syn. nov.** Type: Brazil. “In prov. Minas Geraës”, *Regnell I. 152 part* (lectotype, designated here: R [R000166805]!; isolectotypes: P [P05318036]!, S [S09-12952-online image]!; images of the lectotype and an isolectotype are available at http://plants.jstor.org and http://coldb.mnhn.fr/catalognumber/mnhn/p/p05318036).

Trembleyaphlogiformisvar.stachyoides (Naudin) Cogn. in Martius et al., Fl. Bras. 14(3): 132. 1883. **syn. nov.** Type: Based on *Trembleyastachyoides* Naudin.

Trembleyaphlogiformisvar.villosa Cogn. in Martius et al., Fl. Bras. 14(3): 132. 1883. **syn. nov.** Type: Brazil. “In campis siccis ad Registo Velho”, *Pohl & Schüch 230* (lectotype, designated here: BR [BR0000005223541]!).

*Trembleyaselloana* Cogn. in Martius et al., Fl. Bras. 14(3): 133. 1883. **syn. nov.** Type: Based on Trembleyaphlogiformisvar.glabra Cham.

**8**. ***Trembleyapityrophylla* (Mart. ex DC.) Triana, Trans. Linn. Soc. London 28(1): 24. 1872.** Type: Based on *Osbeckiapityrophylla* Mart. ex DC.

**Synonym of**: *Cambessedesiapityrophylla* (Mart. ex DC.) A.B.Martins.

**9. *Trembleyapradosiana* Netto, Ann. Sci. Nat., Bot. Sér. 5, 3: 378. 1865.** Type: Brazil. “Habitat in campis ad flumen *Rio das Velhas*, prope vivum Trahiras, in parte centrali provinciae Minas Geraes” [Minas Gerais], May 1862, *Netto s.n.* (holotype: P [P00723509]!; image of holotype is available at http://coldb.mnhn.fr/catalognumber/mnhn/p/p00723509).

**Basionym of**: *Microliciapradosiana* (Netto) Versiane & R.Romero.

*Trembleyarubra* Fidanza, A.B.Martins & Almeda, Brittonia 65: 286. 2013. **syn. nov.** Type: Brazil. Minas Gerais, Joaquim Felício, Serra do Cabral, ca. 5 km S do Armazém da Laje, 4 December 2003, *K. Fidanza & R. Belinello 112* (holotype: UEC!; isotype: CAS!).

**10. *Trembleyapurpurascens* Fidanza, A.B.Martins & Almeda, Brittonia 65(3): 284. 2013.** Type: Brazil. Minas Gerais, Joaquim Felício, Serra do Cabral, estrada Joaquim Felício-Armazém, 4 May 2003, *K. Fidanza & R. Belinello 56* (holotype: UEC!; isotype: CAS!).

**Synonym of**: *Microliciacurralensis* Brade.

**11. *Trembleyaserrulata* Fidanza, A.B.Martins & Almeda, Brittonia 65(3): 288. 2013.** Type: Brazil. Minas Gerais, Joaquim Felício, Serra do Cabral, Pedreira, 7 December 2003, *K. Fidanza 108* (holotype: UEC!; isotype: CAS!).

**Replaced with**: *Microliciaserratifolia* Versiane & R.Romero.

**12. *Trembleyathomazii* R.B.Pacifico & Fidanza, Phytotaxa 238(2): 171. 2015.** Type: Brazil. Minas Gerais, Guaraciama, Cadeia do Espinhaço, Serra do Bota, 20 March 2005, *E. Guarçoni 929 & M. A. Sartori* (holotype: MBM!)

**Basionym of**: *Microliciathomazii* (R.B.Pacifico & Fidanza) Versiane & R.Romero.

**13. *Trembleyawarmingii* Cogn. in Martius et al., Fl. Bras. 14(3): 133. 1883.** Type: Brazil. “Ad Lagoa Santa” [Minas Gerais, Lagoa Santa], 9 February 1866, *E. Warming 2306* (holotype: C!).

**Basionym of**: *Poterantherawarmingii* (Cogn.) Almeda & R.B.Pacifico.

##### ﻿Excluded and/or dubious names

1. *Trembleyacapitata* Cogn. [*nom. inval.*]

2. *Trembleyacanescens* Schnizl. [probably = *Pleroma* D.Don]

3. *Trembleyadebilis* Glaz. [*nom. inval.*]

4. *Trembleyasantae-luziae* Glaz. [*nom. inval.*]

5. *Trembleyarhynanthera* Griff. [probably = *Melastoma* L.]

6. *Trembleyarosea* Sessé & Moc. [probably = *Coreopsisgrandiflora* Hogg ex Sweet (Asteraceae)]

## Supplementary Material

XML Treatment for Trembleya s.s. clade of Microlicia

XML Treatment for Microlicia altoparaisensis

XML Treatment for Microlicia calycina

XML Treatment for Microlicia chamissoana

XML Treatment for Microlicia flaviflora

XML Treatment for Microlicia laniflora

XML Treatment for Microlicia parviflora

XML Treatment for Microlicia pentagona

XML Treatment for Microlicia pithyoides

XML Treatment for Microlicia rosmarinoides

XML Treatment for Microlicia trembleyiformis

XML Treatment for Microlicia tridentata

## ﻿Appendix 1

**List of species**

1. *Microliciaaltoparaisensis* (R.B.Pacifico, Almeda & Fidanza) Versiane & R.Romero

2. *Microliciacalycina* (Cham.) Versiane & R.Romero

3. *Microliciachamissoana* (Naudin) Versiane & R.Romero

4. *Microliciaflaviflora* Versiane & R.Romero

5. *Microlicialaniflora* (D.Don) Baill.

6. *Microliciaparviflora* (D.Don) Versiane & R.Romero

7. *Microliciapentagona* (Naudin) Versiane & R.Romero

8. *Microliciapithyoides* (Cham.) Versiane & R.Romero

9. *Microliciarosmarinoides* (Mart. & Schrank ex DC.) Versiane & R.Romero

10. *Microliciatrembleyiformis* Naudin

11. *Microliciatridentata* (Naudin) Versiane & R.Romero

**Specimens examined**

Aguiar 102 (6). Almeda et al. 7726 (5); 7751 (7); 7753 (5); 8395 (5); 8549 (5); 8555 (7); 8580 (3); 8862 (5); 8911 (7); 9076 (6); 9179 (5); 9203 (7). Alves 595 (11); 600 (6); 7359 (11). Alves & Almeida-Lafetá 5573 (5). Alves et al. 2109 (5); 6925 (5). Andrade et al. 374 (5). Antar et al. 1662 (5). Antônio-Silva et al. 315 (7). Araújo et al. 2043 (4); 323 (5); 334 (5). Arbo et al. 3906 (5); 4030 (5). Assis et al. 520 (6). Avezum 6017 & Almeida 10 (6). Azevedo et al. 675 (6). Badini & Ferreira 9774 (11). Baitello et al. 2136 (6). Barreto 10734 (7); 1182 (7); 4621 (6); 4654 (6); 6745 (3); 6757 (6); 6759 (6); 6769 (5); 6771 (5); 6772 (5); 7023 (7); 7024 (7); 7025 (7); 7026 (putative hybrid *M.laniflora* × *M.pentagona*); 706 (5); 9019 (2). Barreto & Viégas s.n. (5). Barros & Feteira 1625 (6). Batista & Naves 405 (6). Baumgratz et al. 1127 (6). Bautista 1347 (6). Beraldo & França 85 (6). Bidá et al. CFCR11951 (4). Borges et al. 153 (5). Bortoluzzi et al. 678 (7). Bovini & Barros 3235 (6). Brade 13735 (5); 14756 (7). Brade et al. 18205 (6). Brandão 14509 (6); 20838 (7); 22298 (6); 28574 (5). Brotto 2600 (6). Brotto & Vieira 1921 (6). Bruniera et al. 37 (5). Bunbury s.n. (6). Bunge s.n. (5). Caddah et al. 636 (6). Caiafa & Umbelino 172 (6). Camargo et al. 109 (6). Carmo 192 (9); 2262 (9); 377 (9); 4819 (5). Carvalho 1086 (6); 6986 (6); s.n. (5). Carvalho et al. 6651 (6). Casaretto s.n. (7). Castro et al. 281 (11). Castro et al. 283 (2); s.n. (5). Cavalcanti CFCR8314 (6). Cavalcanti et al. CFCR8035 (6). Ceccantini et al. 3914 (5). Cerati et al. 246 (4). Chuckr et al. s.n. (5). Claussen 1 (5); 1617 (7); 1631 (11); 1637 (7); 1645 (5); 22 (7); 296 (7); 332A (5); 350 (7); 554 (5); 640 (5); 923 (5); s.n. (5,7). Claussen 10 (2); 368 (2). Colletta 161 (6). Collo et al. s.n. (5). Contro & Marques 21 (7). Cordeiro et al. CFSC10504 (5). Costa 451 (6). Costa et al. 60 (6). Damazio 1025 (5); 1540 (5); 2026 (3); s.n. (277). Davis et al. 2298 (6). Delfini et al. 78 (5); 97 (6). Delprete 9205 (6). Drummond et al. 321 (1). Duarte 2004 (7); 2692 (7); 2767 (10); 8157 (11). Duarte & Brade 1155 (6). Ducke s.n. (5). Eiten & Eiten 7305 (6). Eiten et al. 3019 (6). Emygdio 3312 (6); 3314 (5); 3353 (5); 3613 (6). Escaramai et al. 52 (3); 65 (5). Estevan et al. 617 (6). Farah et al. 580 (6). Faria & Mazucato 105 (5). Faria et al. 1332 (6). Farinaccio et al. 36 (5). Fernandes s.n. (7). Fernandes et al. 1468 (5). Ferreira 5544 (5). Ferreira & Helena 7844 (11). Ferreira et al. 433 (5). Fontana 2309 (6). Fontana et al. 2288 (5); 7678 (6). Forero et al. 7700 (5); 7831 (5). Foresto et al. 59 (6). Forzza et al. 3427 (6); 4897 (4); 6344 (5); 6359 (5); 993 (5). Foster & Leoni 64 (6). Fraga & Saddi 1786 (6). Fraga et al. 3333 (5); 3341 (5). Franco et al. 1270 (5). Furlan et al. CFCR771 (4). Ganev 879 (6); 965 (6). Gardner 379 (6); 380 (6); 4601 (5); 4602 (6). Giulietti & Menezes 4017 (7). Giulietti et al. CFCR13780 (11); CFSC12492 (3); CFSC12560 (3); CFSC12564 (5). Glaziou 11953 (5); 12704 (6); 14740 (5); 14741 (5); 14742 (7); 14743 (11); 14745 (2); 14746 (8); 14747 (9); 16679 (6); 16778 (6); 16781 (11); 17513 (11); 17573 (11); 18232 (2); 19221 (10); 19239 (8); 19242 (9); 21300 (1); 2579 (6); 8680 (6); 9454 (6); s.n. (5). Goldenberg & Michelangeli 2093 (6). Goldenberg & Silveira 1573 (5). Goldenberg et al. 1652 (6). Gonçalves et al. 172 (6). Gounelle s.n. (5). Grandi et al. 6593 (2). Groppo & Ulwin 676 (5). Guarçoni & Sartori 1367 (6). Guedes et al. 12092 (6). Guiamarães 333 (6). Guillemin 921 (6); 946 (6). Hage et al. 2317 (6). Handro 790 (6). Hatschbach 27277 (6); 2775 (6); 39954 (6); 41337 (4); 647 (6); 6908 (6). Hatschbach 26964 (6); 41498 (6). Hatschbach & Guimarães 55455 (6); 56167 (6). Hatschbach & Hatschbach 52005 (4). Hatschbach & Pelanda 28014 (6). Hatschbach & Silva 49393 (6). Hatschbach et al. 27400 (5); 28759 (7); 29958 (7); 42967 (6); 54239 (4); 55434 (6); 68067 (4); 68138 (5); 74974 (6); 75995 (6); s.n. (7). Heluey & Castro 108 (6). Hensold 778 (5). Hensold et al. CFCR2880 (11); CFCR3525 (4). Heringer 8544/736 (6). Hoehne 20521 (66). Hottz et al. 302 (6). Irwin 19974 (5). Irwin et al. 19974 (5); 20178 (7); 20486 (7); 20537 (7); 29029 (11); 29079 (11); 29213 (7); 31141 (6); 34521 (6); 8149 (6). Joly CFSC2405 (5); CFSC82 (5). Joly & Semir CFSC2957 (7); CFSC3196 (7). Kameyama et al. CFSC10403 (3). Kinoshita et al. 43767 (6). Kirizawa 3665 (6). Kirizawa et al. 2132 (6). Klein et al. 2465 (1). Kollmann 231 (6). Kral et al. 72723 (4). Kral et al. 72997 (5). Krieger 10641 (5); 8857 (6). Krieger et al. 24423 (6). Kubo et al. 109 (5); 125 (5). Kuhlmann 4381 (6). Leitão et al. 17281 (5). Leitão Filho et al. 7893 (4). Leoni 1 (6). Lima 400 (6). Lima et al. 1296 (5); 1443 (5); 49 (5); 8445 (6). Loefgren 557 (6). Lombardi 4045 (6). Longhi-Wagner et al. CFCR9184 (11). Lund 135 (5). Macedo 3758 (5). Machado et al. 153 (1). Magalhães 1160 (11); 2278 (7). Maguire et al. 49016 (5); 49140 (5). Mamede et al. 666 (6). Mantovani 99 (5). Marcolino 222 (6). Marcondes-Ferreira et al. 281 (5). Marques et al. 274 (5). Marquete et al. 2332 (6); 3817 (5). Martens 383 (5); 524 (6); 7 (5). Martinelli 19464 (6). Martinelli & Tavora 2583 (5). Martinelli et al. 10932 (6). Martius 930 (5); 931 (6); 961 (6); 989 (6); s.n. (59). Mattos & Rizzini 107 (5); 480 (5). Meireles et al 1362 (5). Meireles et al. 1124 (4); 1766 (6). Mello-Silva et al. 1217 (6); 509 (4); CFCR7836 (6). Mello et al. 61 (5). Mendes & Araújo 970 (6). Mendonça 557 (11). Messias et al. 1922 (5); 2151 (5). Meyer 1171 (1). Michelangeli et al. 1580 (7); 1586 (11); 1595 (11). Mimura 479 (6). Monge et al. 2595 (6); 384 (5). Monteiro 78 (6). Mosen 1971 (6). Moutinho et al. 76 (6). N. da Cruz 135 (6). Nakajima & Romero 3040 (5). Nakajima et al. 1476 (10); 4547 (5); 4764 (4). Netto s.n. (5). Nicolau et al. 2212 (6). Occhioni et al. s.n. (9). Oliveira 1013 (6); 25197 (6); 382 (8). Oliveira & Giacomin 47 (2); 84 (2). Oliveira et al. 478 (7); 480 (2); 508 (6); 518 (7); CFCR12997 (4). Ordones et al. 1856 (5); 213 (7); 84 (11). Orlandi et al. 778 (6). Pacifico 185 (5); 191 (6); 295 (8); 413 (6); 565 (4). Pacifico & Bressan 290 (11); 291 (2); 294 (7); 296 (2); 303 (11); 380 (1); 531 (7); 565 (4). Pacifico & Carmo 154 (3). Pacifico & Simoes 353 (4). Pastore et al. 685 (6). Paula & Grandi s.n. (6). Paula et al. 136 (5); 295 (5); 8 (5). Pedrosa 110 (9); 120 (11). Pena & Viana 365 (5); 417 (3). Pereira 309 (6). Pereira & Pabst 152 (5); 2944 (5); 3107 (5). Pereira & Pabstii 2619 (11). Peron 220 (2); 256 (7); 260 (7); 268 (2); 269 (2). Pirani & Mello-Silva CFCR10814 (4). Pirani & Yano 692 (7); 696 (5). Pirani et al. 1663 (1); 1694 (1); 5082 (5); 696 (5); CFCR11211 (5). Pires & Braga s.n. (5). Pivari 15 (6). Pivari et al. 2512 (7). Polisel et al. 176 (6). Porto 2834 (10). Prates s.n. (6). Queiroz 2808 (6). Raben 409 (6); 428 (5). Rapini et al. 296 (7). Reginato et al. 1401 (5). Rezende 507 (7). Ribas et al. 6769 (6); 8528 (6). Riedel s.n. (255611). Rocha 694 (3). Rocha et al. 497 (4); 602 (5); 672 (5). Rolim 366 (2). Rolim et al. 326 (11); 327 (11); 328 (11); 329 (5); 353 (7); 361 (7); 370 (7); 386 (5); 394 (11); 61 (5). Romero 4157 (10). Romero & Nakajima 3021 (6); 3432 (6); 3593 (10); 5998 (7). Romero et al. 2155 (6); 5303 (7); 5307 (5); 7550 (10); 8627 (3); 8694 (10); 8689 (10). Roque 2869 (6). Roschel et al. s.n. (11). Roth s.n. (5). Rutter 128 (11); 150 (11); 161 (11); 186 (11); 191 (11). Saint-Hilaire 160 [catal. B] (10); 216 (5); 2399 [catal. B2] (11); s.n. (7). Saint-Hilaire s.n. [catal. D, n° 462] (6). Sakuragui & Souza 38 (5). Salatino et al. 14 (5); 18 (5). Salgado 167 (6). Samento & Bautista 859 (6). Sampaio 7194 (5). Santos 411 (10). Saraiva et al. 85 (5). Sazima CFSC4259 (7). Scatigna & Galvão 376 (4). Schüch s.n. (7). Schwacke 10135 (11); 10814 (7); 9184 (9); 9325 (7); 9368 (2). Sellow 1154 (6); 1316 (8); 1446 (5); 1727 (5); 260 (3); 5278 (6); s.n. (3, 5). Semir CFSC2005 (5); CFSC4133 (5); CFSC5001 (5); CFSC5068 (5); CFSC5607 (3). Semir CFSC5608 (7). Semir & Joly CFSC3743 (7). Semir & Sazima CFSC3395 (7). Semir et al CFCR230 (5). Semir et al. 28809 (5). Sena s.n. (3). Sevilha et al. 7075 (6). Sheperd & Kirzenzaft 10214 (3). Silva s.n. (6). Silva & Abe 2310 (6). Silva & Grandi 6627 (6). Silva & Koch 7610 (6). Silva et al. 1043 (7); 6478 (6). Silveira s.n. (3). Sobral et al. 12797 (6); 14085 (6); 14508 (7). Souza 1606 (5). Souza & Miranda 1639 (3). Souza et al. 1013 (6); 1108 (6); 11565 (5); 25181 (5); 2584 (9); 5934 (6); 6098 (6); 8015 (11); 8047 (11); 8061 (11). Stehmann 2532 (6). Stehmann & Morais 2354 (5); 2650 (5). Tales et al. 17 (5). Tameirao Neto & Mansur 4874 (5). Tannus 760 (6). Teixeira s.n. (5); s.n. (5). Temponi & Vasconcelos s.n. (5). Torres s.n. (5). TSMG & Tales 81 (5). Ule 2528 (9); 2543 (7). Vainio 33501 (7). Valente & Azevedo 57 (6). Valente et al. 1229 (5); 1230 (5); 2574 (5); 535 (5). Vasconcelos s.n. (55). Vauthier 37 (5); 43 [part] (6); s.n. (5). Verdi et al. 6501 (5). Versiane & Devides 677 (9); 682 (11). Versiane et al. 305 (6). Vidal s.n. (9). Vieira & Yamamoto 26186 (6). Wängler & Ferreira 1352 (6). Warming s.n. (6). Weddell s.n. (8). Widgren 967 (6); s.n. (6). Williams & Assis 6352 (5); 7274 (6). Yamamoto 1549 (6). Zappi et al. 2504 (6); CFCR9903 (4); CFSC9353 (5).

## Supplementary materials

Supplementary material 1 List of species sampled in this study, their respective Genbank accession codes (for nrITS and nrETS), and specimen vouchers Data type table (word document) Explanation note List of species sampled in this study, their respective Genbank accession codes (for nrITS and nrETS), and specimen vouchers. Codes highlighted in bold refer to sequences previously obtained from GenBank. https://binary.pensoft.net/file/784282This dataset is made available under the Open Database License (http://opendatacommons.org/licenses/odbl/1.0/). The Open Database License (ODbL) is a license agreement intended to allow users to freely share, modify, and use this Dataset while maintaining this same freedom for others, provided that the original source and author(s) are credited.Ricardo Pacifico, Frank Almeda, Darin S. Penneys, Karina Fidanza
