# Supplementary material for: ﻿Systematics of the Trembleya sensu stricto clade of Microlicia (Melastomataceae, Lavoisiereae)
Source: PhytoKeys. 2022 Dec 20;216:1–101. doi: 10.3897/phytokeys.216.91032 (PMC9836420; doi:10.3897/phytokeys.216.91032)
Supplement: Supplementary material 1 — List of species sampled in this study, their respective Genbank accession codes (for nrITS and nrETS), and specimen vouchers [file phytokeys-216-001_article-91032__-s001.doc]

**Supplementary material 1.** List of species sampled in this study, their respective Genbank accession codes (for nrITS and nrETS), and specimen vouchers. Codes highlighted in bold refer to sequences previously obtained from GenBank.

| **Species** | **nrITS** | **nrETS** | **Voucher** |
| --- | --- | --- | --- |
| *Acisanthera quadrata* Pers | OP627677 | OP573763 | D.S. Penneys 1703 (CAS) |
| *Blakea anomala* Donn. Sm | - | OP573764 | D.S. Penneys 1498 (CAS) |
| *Cambessedesia glaziovii* Cogn. ex A.B.Martins | OP627678 | OP573765 | F. Almeda 7859 (CAS) |
| *C. harleyi* Wurdack | OP627679 | OP573766 | F. Almeda 8248 (CAS) |
| *Graffenrieda cucullata* (D.Don) L.O.Williams | OP627688 | OP573775 | D.S. Penneys 1873 (CAS) |
| *Heterocentron muricatum* Gleason | OP627689 | OP573778 | D.S. Penneys 1949 (CAS) |
| *Microlicia acuminifolia* Versiane & R.Romero | OP627784 | OP573874 | K.F. Rodrigues 233 (UEC) |
| *M.* adamantium (Barreto ex Pedersoli) Versiane & R.Romero | OP627690 | OP573777 | F. Almeda 9605 (CAS) |
| *M. alba* (Mart. & Schrank ex DC.) Versiane & R.Romero | OP627691 | OP573780 | F. Almeda 8388 (CAS) |
| *M. albiflora* (Naudin) Versiane & R.Romero | OP627680 | OP573767 | F. Almeda 7713 (CAS) |
| *M. altoparaisensis* (R.B.Pacifico, Almeda & Fidanza) Versiane & R.Romero | - | OP573858 | R. Pacifico 380 & V.E. Bressan (HUEM) |
| *M. amblysepala* Ule | - | **AY553738** | F. Almeda 8308 (CAS) |
| *M. arachnoidea* (Almeda & A.B.Martins) Versiane & R.Romero | OP627692 | OP573779 | K.F. Rodrigues 572 (UEC) |
| *M. armata* (Spreng.) Versiane & R.Romero | OP627684 | OP573771 | F. Almeda 7760 (CAS) |
| *M. armata* (Spreng.) Versiane & R.Romero | OP627681 | OP573768 | F. Almeda 9723 (CAS) |
| *M. australis* (A.St-Hil. ex Naudin) Versiane & R.Romero | OP627709 | OP573796 | F. Almeda 9830 (CAS) |
| *M. baccharoides* Schrank & Mart. ex Naudin | OP627719 | - | F. Almeda 8282 (CAS) |
| *M. calycina* (Cham.) Versiane & R.Romero | OP627751 | OP573835 | R. Pacifico 296 (HUEM) |
| *M. canastrensis* Naudin | OP627777 | OP573867 | R. Pacifico 408 (HUEM) |
| *M. capitata* R.B.Pacifico, Almeda & Fidanza | OP627740 | OP573826 | R. Pacifico 250 (HUEM) |
| *M. caryophyllea* (Naudin) Versiane & R.Romero | OP627694 | OP573782 | F. Almeda 8567 (CAS) |
| *M. cataphracta* (Mart. & Schrank ex DC.) Versiane & R.Romero | OP627703 | OP573790 | F. Almeda 9601 (CAS) |
| *M. cataphracta* (Mart. & Schrank ex DC.) Versiane & R.Romero | OP627707 | OP573794 | R. Goldenberg 820 (UPCB) |
| *M. chamissoana* (Naudin) Versiane & R.Romero | OP627785 | OP573761 | R. Pacifico 154 (HUEM) |
| *M. chrysantha* Wurdack | OP627721 | OP573805 | F. Almeda 8309 (CAS) |
| *M. chrysoglandulosa* R.Romero et al. | - | OP573863 | R. Pacifico 386 (HUEM) |
| *M. ciliatoglandulosa* | - | OP573856 | Pacifico 372 (HUEM) |
| *M. clavillosa* Wurdack | OP627685 | OP573772 | F. Almeda 9440 (CAS) |
| *M. cogniauxiana* R. Romero | OP627758 | OP573840 | R. Pacifico 317 (HUEM) |
| *M. confertiflora* Naudin | OP627722 | OP573806 | K.F. Rodrigues 204 (UEC) |
| *M. congestiflora* Versiane & R.Romero | OP627696 | OP573784 | F. Almeda 8568 (CAS) |
| *M. cordata* (Spreng.) Cham | - | **AY553739** | F. Almeda 8402 (CAS) |
| *M. cordifolia* Versiane & R.Romero | OP627697 | OP573785 | F. Almeda 9696 (CAS) |
| *M. crassa* R. Romero | - | OP573849 | R. Pacifico 355 (HUEM) |
| *M. crassifolia* (Mart. & Schrank ex DC.) Versiane & R.Romero | OP627698 | OP573786 | F. Almeda 8442 (CAS) |
| *M. crebropunctata* Pilg | OP627774 | OP573861 | R. Pacifico 384 (HUEM) |
| *M. crenulata* (DC.) Mart | OP627748 | OP573832 | R. Pacifico 293 (HUEM) |
| *M. cryptandra* Naudin | OP627773 | OP573860 | R. Pacifico 383 (HUEM) |
| *M. cupressina* D.Don | OP627682 | OP573769 | F. Almeda 8367 (CAS) |
| *M. curralensis* Brade | OP627769 | OP573776 | R. Pacifico 363 (HUEM) |
| *M. curralensis* Brade | - | OP573853 | K.F. Rodrigues 56 (UEC) |
| *M. curtiana* Versiane & R.Romero | OP627693 | OP573781 | F. Almeda 9718 (CAS) |
| *M. daviesiana* (Almeda & A.B.Martins) Versiane & R.Romero | OP627766 | OP573850 | R. Pacifico 356 (HUEM) |
| *M. decipiens* Naudin | OP627761 | OP573843 | R. Pacifico 337 (HUEM) |
| *M. donii* Fidanza & R.B.Pacifico | OP627739 | OP573825 | R. Pacifico 249 (HUEM) |
| *M. euphorbioides* Mart | - | OP573866 | Pacifico 406 (HUEM) |
| *M. fasciculata* Mart. ex Naudin | - | OP573855 | R. Pacifico 378 (HUEM) |
| *M. fasciculata* Mart. ex Naudin | OP627770 | - | Pacifico 371 (CAS) |
| *M. flava* R.Romero | OP627743 | OP573828 | R. Pacifico 279 (HUEM) |
| *M. flaviflora* Versiane & R.Romero | OP627765 | OP573848 | R. Pacifico 353 (HUEM) |
| *M. flavipetala* Versiane & R.Romero | - | OP573859 | R. Pacifico 382 (HUEM) |
| *M. gentianoides* (Mart. & Schrank ex DC.) Versiane & R.Romero | OP627699 | OP573787 | F. Almeda 8314 (CAS) |
| *M. glandulifolia* Versiane & R.Romero | OP627700 | - | Almeda 7780 (CAS) |
| *M. glazioviana* Cogn. | OP627747 | OP573831 | R. Pacifico 292 (HUEM) |
| *M. glaziovii* (Cogn.) Versiane & R.Romero | OP627683 | OP573770 | Almeda 9803 (CAS) |
| *M. graveolens* Mart. & Schrank ex DC. | OP627724 | OP573808 | K.F. Rodrigues 720 (UEC) |
| *M. hilairei* Versiane & R.Romero | OP627695 | OP573783 | F. Almeda 9606 (CAS) |
| *M. hirticalyx* R.Romero & Woodgyer | OP627744 | - | R. Pacifico 283 (HUEM) |
| *M. inquinans* Naudin | OP627778 | OP573868 | R. Pacifico 410 (HUEM) |
| *M. intercalycina* Pataro & R.Romero | - | **AY553736** | F. Almeda 8280 (CAS) |
| *M. inversa* (Fidanza, A.B.Martins & Almeda) Versiane & R.Romero | OP627786 | OP573875 | K.F. Rodrigues 667 (UEC) |
| *M. isophylla* Schrank & Mart. ex DC. | OP627725 | OP573809 | F. Almeda 9827 (CAS) |
| *M. itambana* (Mart. & Schrank ex DC.) Versiane & R.Romero | OP627704 | OP573791 | F. Almeda 9663 (CAS) |
| *M. juniperina* A.St.-Hil | - | **AY553741** | F. Almeda 8547 (CAS) |
| *M. laniflora* (D.Don) Bail. | OP627787 | OP573876 | K.F. Rodrigues 179 (UEC) |
| *M. longicalycina* R. Romero | OP627760 | OP573842 | R. Pacifico 322 (HUEM) |
| *M. longipedicellata* (Cogn.) Almeda & A.B.Martins | OP627764 | OP573847 | R. Pacifico 349 (HUEM) |
| *M. macrantha* Versiane & R.Romero | OP627701 | OP573788 | F. Almeda 9459 (CAS) |
| *M. macrocarpa* (Naudin) Versiane & R.Romero | OP627705 | OP573792 | F. Almeda 8545 (CAS) |
| *M. macrophylla* Naudin | OP627735 | OP573821 | R. Pacifico 205 (HUEM) |
| *M. minima* Markgr. | OP627726 | OP573810 | F. Almeda 8323 (CAS) |
| *M. minor* Versiane & R.Romero | OP627702 | OP573789 | F. Almeda 9626 (CAS) |
| *M. mucorifera* (Mart. & Schrank ex DC.) Versiane & R.Romero | OP627706 | OP573793 | F. Almeda 9558 (CAS) |
| *M. multicaulis* Mart. ex Naudin | OP627753 | - | R. Pacifico 298 (HUEM) |
| *M. mutabilis* R.B.Pacifico, Almeda & Fidanza | OP627771 | OP573857 | R. Pacifico 379 (HUEM) |
| *M. naudiniana* R.Romero | OP627759 | OP573841 | R. Pacifico 321 (HUEM) |
| *M. neopyrenaica* (Naudin) Versiane & R.Romero | OP627788 | OP573877 | F. Almeda 7870 (CAS) |
| *M. nortecipoana* R.B. Pacifico, Fidanza & Almeda | OP627736 | OP573822 | R. Pacifico 207 (HUEM) |
| *M. obtusifolia* Cogn. ex Romero | - | OP573864 | R. Pacifico 400 (HUEM) |
| *M. oligochaeta* Wurdack | - | OP573762 | F. Almeda 8275 (CAS) |
| *M. pabstii* Brade | OP627756 | - | R. Pacifico 308 (HUEM) |
| *M. parviflora* (D.Don) Versiane & R.Romero | OP627789 | OP573878 | F. Almeda 8483 (CAS) |
| *M. pentagona* (Naudin) Versiane & R.Romero | OP627790 | OP573879 | F. Almeda 8555 (CAS) |
| *M. pentagona* (Naudin) Versiane & R.Romero | OP627749 | OP573833 | R. Pacifico 294 (HUEM) |
| *M. phlogiformis* (Mart. & Schrank ex DC.) Versiane & R.Romero | OP627791 | OP573880 | F. Almeda 7748 (CAS) |
| *M. pilosa* Versiane & R.Romero | OP627715 | - | F. Almeda 9672 (CAS) |
| *M. pinheiroi* Wurdack | - | **AY553737** | F. Almeda 8301 (CAS) |
| *M. pithyoides* (Cham.) Versiane & R.Romero | OP627750 | OP573834 | R. Pacifico 295 (HUEM) |
| *M. pohliana* (O.Berg ex Triana) Versiane & R.Romero | OP627708 | OP573795 | F. Almeda 9423 (CAS) |
| *M. polychaeta* R.B.Pacifico, Almeda & Fidanza | - | OP573854 | R. Pacifico 370 (HUEM) |
| *M. psammophila* Wurdack | OP627775 | OP573862 | R. Pacifico 385 (HUEM) |
| *M. pulcherrima* (Mart. & Schrank ex DC.) Versiane & R.Romero | OP627710 | OP573797 | F. Almeda 9666 (CAS) |
| *M. punctata* (Mart. & Schrank ex DC.) Versiane & R.Romero | OP627711 | OP573798 | F. Almeda 9709 (CAS) |
| *M. repanda* R.B.Pacifico, Almeda & Fidanza | OP627792 | OP573881 | K.F. Rodrigues 189 (UEC) |
| *M. rigida* (Cogn.) Versiane & R.Romero | OP627712 | OP573799 | F. Almeda 9544 (CAS) |
| *M. rosmarinoides* (Schrank & Mart. ex DC.) Versiane & R.Romero | OP627793 | OP573882 | Vidal s.n. (BHCB-181346) |
| *M. rugosa* R.Romero & Versiane | OP627737 | OP573823 | R. Pacifico 213 (HUEM) |
| *M. rundeliana* (Almeda & A.B.Martins) Versiane & R.Romero | OP627713 | - | F. Almeda 9667 (CAS) |
| *M. sampaioana* (Barreto) Versiane & R.Romero | OP627714 | - | F. Almeda 9673 (CAS) |
| *M. scaberula* (Naudin) Versiane & R.Romero | OP627763 | OP573846 | R. Pacifico 346 (HUEM) |
| *M. selaginea* Naudin | OP627686 | OP573773 | F. Almeda 7854 (CAS) |
| *M. serratifolia* Versiane & R.Romero | OP627741 | OP573827 | R. Pacifico 260 (HUEM) |
| *M. serrulata* Cham | OP627757 | OP573839 | R. Pacifico 316 (HUEM) |
| *M. setosa* (Spreng.) DC. | OP627755 | OP573838 | R. Pacifico 304 (HUEM) |
| *M. sparsifolia* R.B.Pacifico, Almeda & Fidanza | OP627780 | OP573870 | Sakuragui CFCR15337 (K) |
| *M. speciosa* Versiane & R.Romero | OP627745 | OP573829 | R. Pacifico 287 (HUEM) |
| *M. stenocladon* Naudin | OP627687 | OP573774 | E. Salviani 2268 (UEC) |
| *M. suberosa* (Naudin) Versiane & R.Romero | OP627782 | OP573872 | F. Almeda 7861 (CAS) |
| *M. subulata* (Triana) Versiane & R.Romero | - | OP573800 | F. Almeda 8550 (CAS) |
| *M. tenuifolia* R.Romero | OP627738 | OP573824 | R. Pacifico 239 (HUEM) |
| *M. thomazii* (R.B.Pacifico & Fidanza) Versiane & R.Romero | OP627767 | OP573851 | R. Pacifico 359 (HUEM) |
| *M. tomentella* Naudin | OP627727 | OP573811 | K.F. Rodrigues 764 (UEC) |
| *M. torrendii* Brade | - | **AY553735** | F. Almeda 8250 (CAS) |
| *M. trembleyiformis* Naudin | - | OP573820 | R. Romero 4157 (HUFU) |
| *M. tridentata* (Naudin) Versiane & R.Romero | OP627746 | OP573830 | R. Pacifico 290 (HUEM) |
| *M. viminalis* (DC.) Triana | OP627728 | OP573812 | F. Almeda 8258 (CAS) |
| *M.* cf. a*mplexicaulis* Cogn. | OP627717 | OP573802 | K.F. Rodrigues 211 (UEC) |
| *M.* cf. *chrysoglandulosa* R.Romero et al. | OP627718 | OP573803 | K.F. Rodrigues 637 (UEC) |
| *M.* sp. 1 | OP627716 | OP573801 | Medeiros s.n. (FLOR-0029497) |
| *M.* sp. 2 | OP627781 | OP573871 | K.F. Rodrigues 334 (UEC) |
| *M.* sp. 3 | OP627754 | OP573837 | R. Pacifico 299 (HUEM) |
| *M.* sp. 4 | OP627762 | OP573844 | R. Pacifico 338 (HUEM) |
| *M.* sp. 5 | OP627779 | OP573869 | R. Pacifico 411 (HUEM) |
| *M.* sp. 6 | OP627776 | OP573865 | R. Pacifico 404 (HUEM) |
| *M.* sp. 7 | OP627720 | OP573804 | K.F. Rodrigues 632 (UEC) |
| *M.* sp. 8 | OP627723 | OP573807 | K.F. Rodrigues 217 (UEC) |
| *M.* sp. 9 | OP627768 | OP573852 | R. Pacifico 362 (HUEM) |
| *M.* sp. 10 | - | OP573845 | R. Pacifico 340 (HUEM) |
| *M.* sp. 11 | OP627752 | OP573836 | R. Pacifico 297 (HUEM) |
| *M.* sp. 12 | OP627742 | - | R. Pacifico 277 (HUEM) |
| *Poteranthera* *pusilla* Bong. | **KU845167** | **KU845164** | M.J.R. Rocha 940 (BHCB) |
| *Rhexia* *salicifolia* Kral & Bostick | OP627729 | OP573813 | J.R.A. 14995 (CAS) |
| *R*. *virginica* L | OP627730 | OP573814 | Ionta 202 (FLAS) |
| *Rhynchanthera* *brachyrhyncha* Cham | OP627731 | OP573815 | F. Almeda 9883 (CAS) |
| *R*. *bracteata* Triana | OP627732 | OP573816 | F. Almeda 8801 (CAS) |
| *R*. *cordata* DC. | OP627733 | OP573817 | F. Almeda 7843 (CAS) |
| *R.* *grandiflora* (Aubl.) DC. | OP627734 | OP573818 | D.S. Penneys 1922 (CAS) |
| *R.* *serrulata* (L.C.Rich.) DC. | OP627794 | OP573819 | F.A. Michelangeli 828 (CAS) |
| *Tibouchina* *fothergillae* (Schrank & Mart. ex DC.) Cogn | OP627783 | OP573873 | F. Almeda 7714 (CAS) |
